# A systematic study of the interplay between guest molecule structure and intermolecular interactions in crystalline sponges

**DOI:** 10.1107/S2052252523005146

**Published:** 2023-07-01

**Authors:** Robert C. Carroll, David C. Harrowven, James E. Pearce, Simon J. Coles

**Affiliations:** a School of Chemistry, University of Southampton, University Road, Southampton, Hampshire SO17 1BJ, United Kingdom; Formby, Liverpool, United Kingdom

**Keywords:** crystalline sponge, systematic studies, metal–organic frameworks, single-crystal X-ray diffraction, intermolecular interactions, MOFs

## Abstract

Utilization of the crystalline sponge {[(ZnI_2_)_3_(tpt)_2_·*x*(solvent)]_*n*_} method has enabled characterization of a novel family of synthetic organic oils. The systematic structural differences and diversity of functional groups offered by 13 related molecular adsorbates provide a detailed quantitative understanding of the relationship between the guest structure, its conformation, and the type of intermolecular interactions adopted with neighbouring guests and the host framework. This analysis is extended to assess the connection of these factors to the resulting quality indicators for a particular molecular structure elucidation.

## Abstract 2

A systematic study of a related set of crystalline sponge adsorbate compounds has enabled the investigation of the interplay between guest molecular structure and intermolecular interactions, which furthers our fundamental understanding of the applicability of this characterization method.

## 1. Introduction

The crystalline sponge (CS) method, first published by Inokuma *et al.* (2013 ▸), increases the scope of crystal structure analysis by applying this characterization method to entirely new types and phases of compounds. Utilization of crystalline, porous, solvent-filled metal–organic frameworks (MOFs) allows for encapsulation and characterization of guest molecules by single-crystal X-ray diffraction (SCXRD). This is possible when guests adopt regular positions throughout the framework, thereby providing the long-range ordering necessary for Bragg scattering. The use of pre-grown crystalline hosts removes the bottleneck of sample crystallization associated with traditional crystallography. It can even be applied to liquids and oils and can be performed with very small amounts of the analyte (Kawahata *et al.*, 2016 ▸; Yoshioka *et al.*, 2016 ▸; Zigon *et al.*, 2017 ▸; Wada *et al.*, 2018 ▸; Habib *et al.*, 2020 ▸). A range of applications of the CS method are detailed in two comprehensive reviews (Zigon *et al.*, 2021 ▸; Du *et al.*, 2018 ▸).

The MOF most commonly associated with CS analysis is {[(ZnX_2_)_3_(tpt)_2_·*x*(solvent)]_*n*_}, where X = Cl or I, and tpt = tris­(4-pyridyl)-1,3,5-triazine. There are two key features of the host which allow it to effectively encapsulate and order guest molecules. First, the solvent-filled pores enable thermodynamic exchange of target molecules with weakly interacting host solvent. The controlled diffusion of molecules through MOF cavities assists the action of the second feature: molecular recognition points. These points are regions of the framework that are favourable for interaction and hence are commonly adopted by guest molecules. In particular, the tpt linkers provide highly aromatic, electron-deficient, planar surfaces for interaction. These hydro­phobic regions can adopt a variety of contacts with guests via the pyridine and triazine rings, as well as hydrogen bonding with nitro­gen atoms in tpt ligands and negatively charged iodine from ZnI­_2_ units. The lack of specificity for interactions with the framework results in compatibility with a diverse range of compounds and chemistries. It also offers a variety of sites within cavities which multiple molecules can concurrently occupy.

Although the method has significant potential for application to most areas of research, adoption of the technique has been primarily limited to the pharmaceutical industry (Rosenberger *et al.*, 2020 ▸, 2021 ▸) and academic work has rarely strayed outside of the original authors’ laboratories.^1^Although there are some publications that report a series of related compounds, they are focused on molecular structure elucidation (Wada *et al.*, 2021 ▸; Taniguchi *et al.*, 2022 ▸). Consequently, an area which has yet to be fully studied, understood and taken advantage of is the relationship between the molecular structure of the guest and the interactions it adopts within the CS host. This lack of fundamental understanding results in an inability to rationalize why related compounds behave differently, *e.g.* either requiring unique exchange conditions or being unable to fully elucidate the analyte structure. This problem highlights a more overarching theme in the CS area: an absence of systematic investigation. To date, only two studies by Carmalt and co-workers have sought to apply a systematic approach to understanding the nature of the pore positions adopted by seven or eight structurally related molecules, where a consideration of intermolecular interactions has been included (Hayes *et al.*, 2016 ▸, 2017 ▸).

Here we present 13 closely related bi­aryl analyte molecules, synthesized at the University of Southampton (Pearce, 2022 ▸), which have been characterized by the CS method. These molecules represent a selection from a wider compound library and have been chosen to investigate the fundamental interactions that enable the CS method to work. The size of the group and its structural diversity allow for analysis beyond an individual structure which aims to identify principles for crystal sponge–analyte affinity akin to those that define crystal engineering (Desiraju, 1989 ▸). This work also seeks to showcase the suitability of this technique to the diversity of chemistry studied in academia and thereby encourage further application beyond pharmaceutical-based compounds.

These analytes can be allocated across five groups designed to investigate specific structural influences. These are sterically demanding substituents in a variety of positions (Group 1), nitrile functionalities (Group 2), sterically and electronically demanding meth­oxy substituents (Group 3), multiple halogen types with varying position and number (Group 4), and sterically demanding and aromatic substituents (Group 5).

## 2. Methodology

### 2.1. Compound structures and atom-labelling scheme

**Figure 1:**
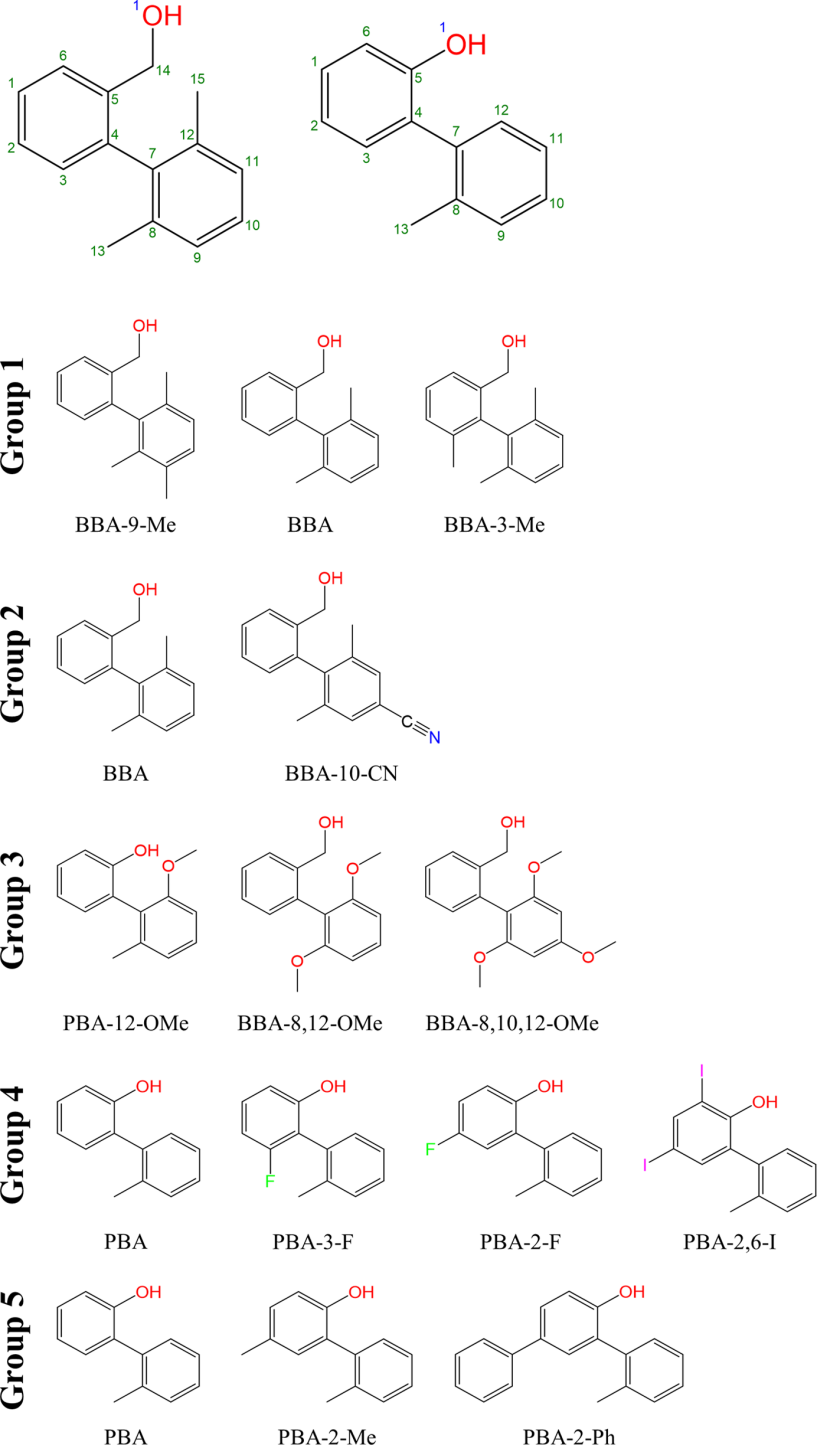
Exemplary guest numbering schemes of PBAs and BBAs (top) with group composition (bottom).

The five groups of molecules can be further characterized according to molecular similarity as measured by the Tanimoto coefficient (Tanimoto, 1957 ▸). There are two types of guest molecule analysed within the study: benzyl bi­aryl alcohols (BBAs) and phenol bi­aryl alcohols (PBAs). The numbering scheme and composition of the groups are represented in Fig. 1 ▸ and a full molecular similarity matrix is given in Table S4 of the supporting information. An illustrative numbering scheme for atoms in the host framework is available in Fig. S1 of the supporting information.

### 2.2. Guest occupancy determination

For consistent determination of guest occupancies and therefore reliable comparison between structures, in all cases non-hydrogen atoms were refined isotropically with molecule free-variable (FVAR) and restrained thermal parameters (*U*_iso_ = 0.08). The guest occupancies were then fixed before anisotropic refinement to prevent expansion of thermal ellipsoids and erroneous assignment of residual electron density within the porous framework. In some cases, low-occupancy guests and disordered exchange sites prevented stable anisotropic refinement. Rather than applying severe thermal restraints, these molecules were modelled isotropically with restrained thermal parameters (*U*_iso_ = 0.08). Further details on the crystallographic refinement strategy can be found in Section S1 of the supporting information.

## 3. Results and discussion

### 3.1. Group 1

#### 3.1.1. Background and interaction overview

Group 1 contains BBA-9-Me, BBA and BBA-3-Me with molecular structures as shown in Fig. 1 ▸. The three molecules vary in structure by the number and location of methyl substituents. This substitution results in minimal changes in electronic character, owing to its limited potential for generating new intermolecular interactions, while allowing specific tuning of steric properties and 3D molecular shape.

**Figure 2:**
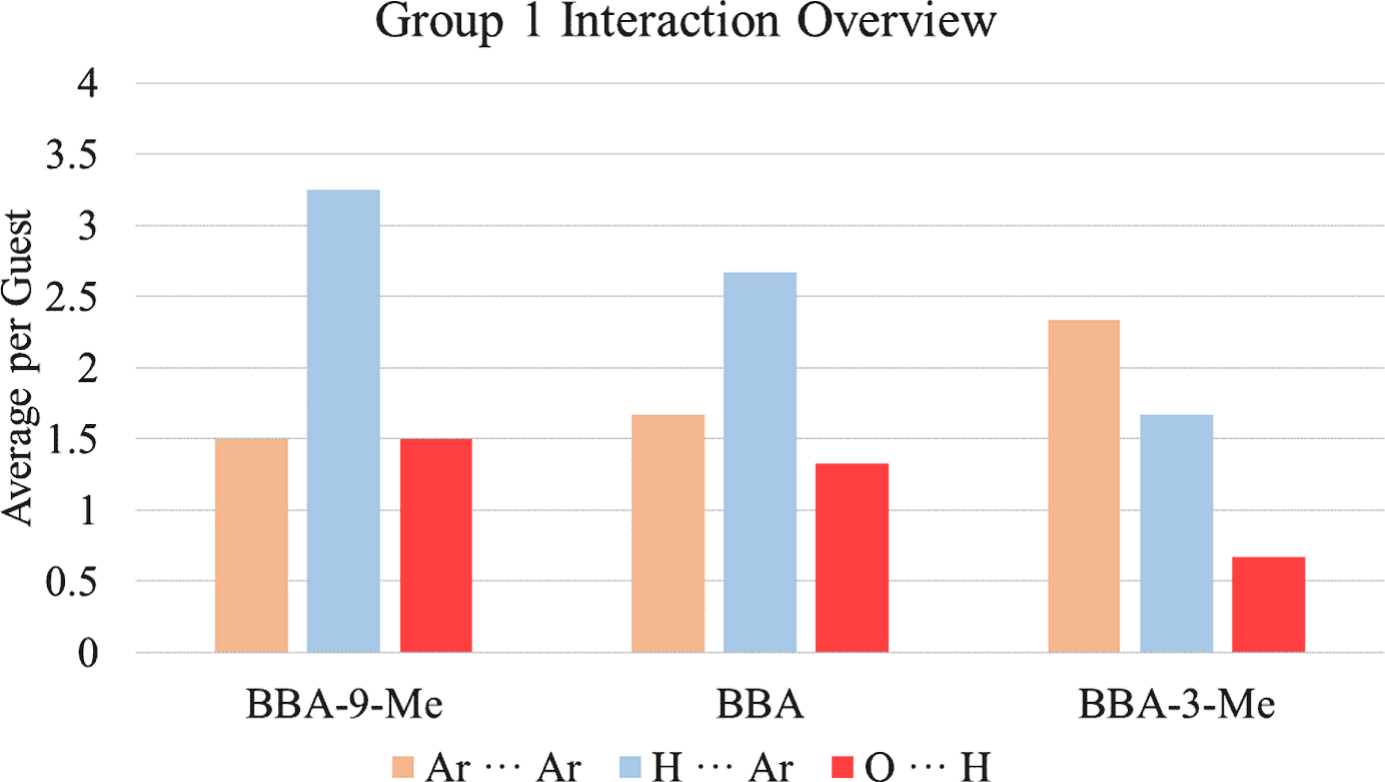
Average interactions per guest for BBA-9-Me, BBA and BBA-3-Me.

In Group 1, the interactions of interest are aromatic–aromatic (Ar⋯Ar), hydrogen–aromatic (H⋯Ar) and oxygen–hydrogen (O⋯H). The criteria and process employed for identification of interactions are detailed in Section S2 of the supporting information. The interactions are summarized in Fig. 2 ▸ and a full list of interactions and group summaries for Group 1, as well as all other groups, can be found in Sections S6 and S7 of the supporting information.

#### 3.1.2. Aromatic interaction motifs

**Figure 3:**
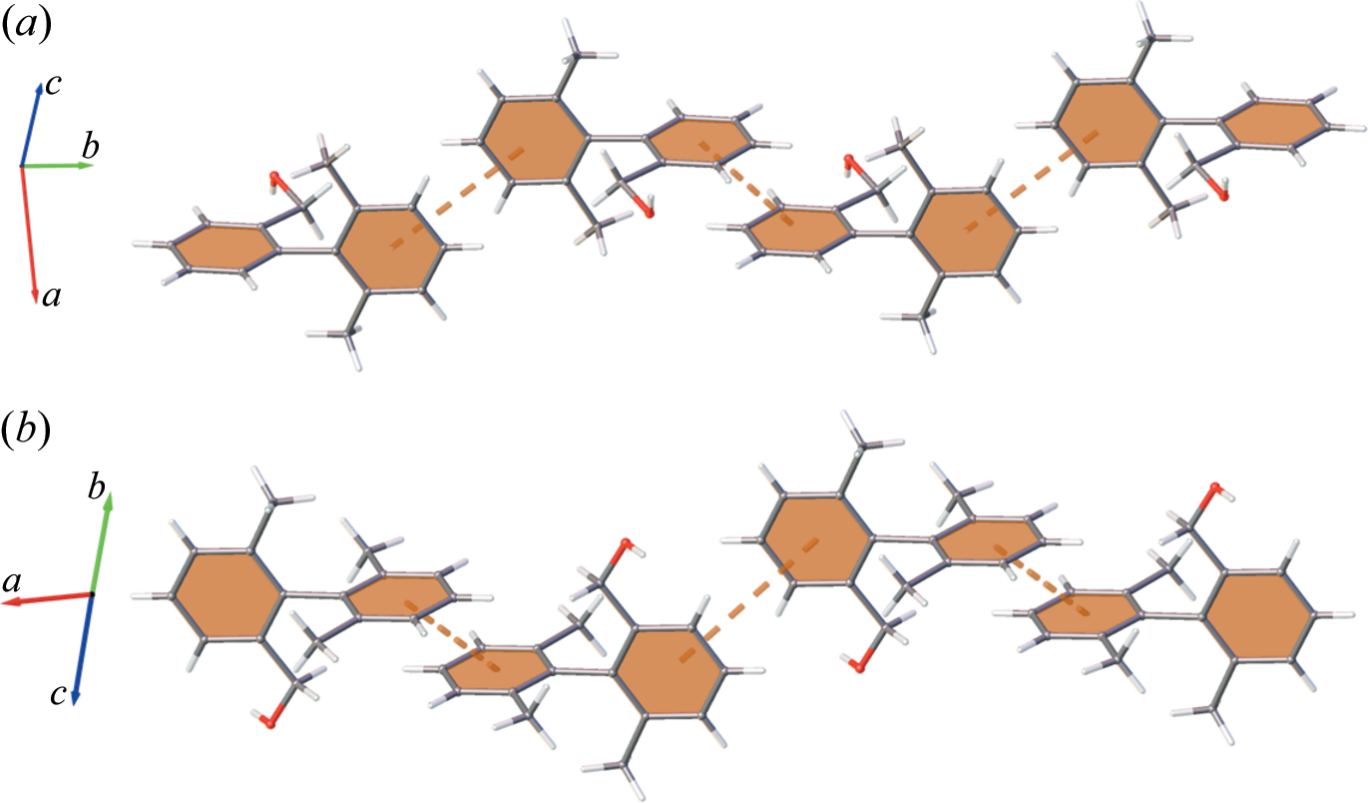
Complementary C1–C6 and C7–C12 Ar⋯Ar arrangements of (*a*) BBA and (*b*) BBA-3-Me, with symmetry-generated molecules, framework and additional guest sites omitted for clarity.

The first trend observed is a steady increase in Ar⋯Ar across the series. Further investigation reveals that these are predominantly host–guest interactions with the framework. However, it is the guest–guest interactions and the motifs adopted which provide insight into the Ar⋯Ar interaction trend and the steric influence of methyl substitution. BBA-9-Me can only form Ar⋯Ar contacts with its C1–C6 ring and relies on other interactions to accommodate the C7–C12 ring in the structure. In contrast, BBA and BBA-3-Me form complementary Ar⋯Ar interactions with both rings, as shown in Fig. 3 ▸.

This behaviour occurs because BBA and BBA-3-Me can adopt ‘tail-to-tail’ arrangements which allow Ar⋯Ar contact without steric clashing of methyl groups. In contrast, the additional methyl on the C7–C12 ring for BBA-9-Me would result in all possible arrangements possessing clashing, non-complementary methyl groups. Consequently, BBA-9-Me adopts the fewest Ar⋯Ar interactions in the series.

#### 3.1.3. Exchange site analysis

**Figure 4:**
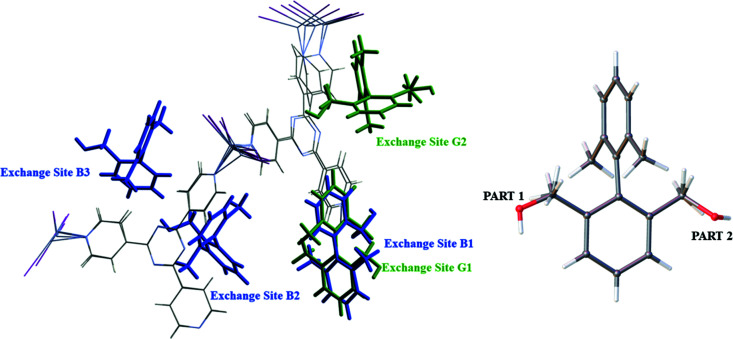
Overlay of asymmetric units for BBA (blue) and BBA-3-Me (green), alongside the BBA-3-Me exchange site G2 disorder.

To rationalize the increase in Ar⋯Ar interaction between BBA and BBA-3-Me, consideration of torsion angles between the aromatic rings is required. Both compounds have three guest molecules modelled in the asymmetric unit, but BBA-3-Me possesses a smaller torsion angle range (0.85° versus 3.52°). This small physical change is likely to be a consequence of the increased steric hindrance introduced by the additional methyl group at the C3 position, near the central aryl–aryl C–C bond. The restricted torsion angle results in the adoption of only two exchange sites for BBA-3-Me, compared with three exchange sites for BBA, visualized in Fig. 4 ▸.

This arrangement is notable because of the disorder observed at exchange site G2 for BBA-3-Me, where the aromatic ring positions are consistent but there is variation of the benzyl alcohol arm. It is suggested that the more restricted torsion angle of BBA-3-Me enables adoption of almost identical locations from multiple orientations, thereby maximizing Ar⋯Ar interaction. In contrast, the greater variation of the torsion angle for BBA results in adoption of three individual sites which cannot all utilize aromatic contacts to the same extent. This highlights the influential role that molecular shape and Ar⋯Ar contacts play in determining the guest location and orientation within the pore. Consideration of this behaviour, alongside the matching primary exchange sites (B1 and G1) for BBA and BBA-3-Me, provides further evidence for the reliability of guest positions described in previous CS studies (Hayes *et al.*, 2016 ▸). The disorder of molecules is also observed for other compounds in this study (discussed in Section S4 of the supporting information) and acts as an important reminder that the CS method characterizes ‘isolated’ molecules in cavities throughout the framework.

The second trend identified for Group 1 is a decrease in H⋯Ar interaction across the series. The prevalence of this contact for BBA-9-Me and BBA compensates for the reduced formation of Ar⋯Ar contacts, a hypothesis that is supported by two observations. The first is the involvement of BBA-9-Me aromatic ring centroids in 72% of all the measured H⋯Ar contacts. The second is the participation of the BBA C exchange site in 50% of the observed H⋯Ar interactions, while forming no Ar⋯Ar contacts. Therefore, BBA-9-Me must adopt the greatest number of H⋯Ar contacts to stabilize the structure, because it is aided the least by Ar⋯Ar contacts and the contribution from H⋯Ar is then required less across the series.

#### 3.1.4. Benzyl alcohol arm rotation

**Figure 5:**
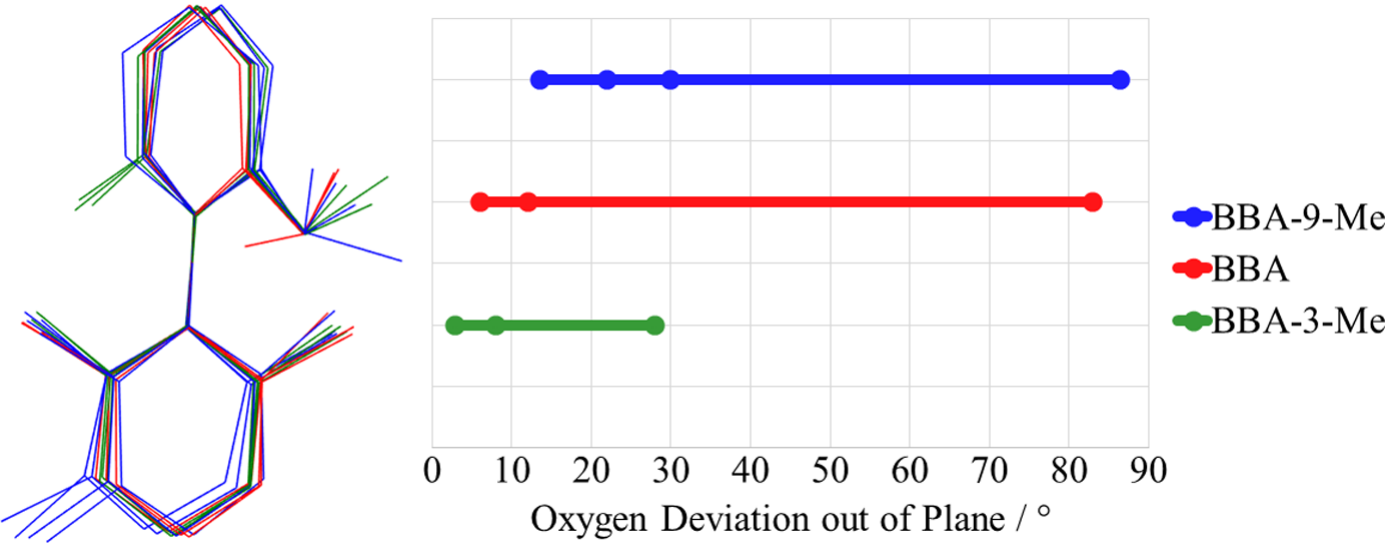
Comparison of benzyl alcohol arm conformational flexibility for Group 1.

Finally, the number of O⋯H contacts reduces across the series but with a significantly smaller range than H⋯Ar contacts. The two interactions play similar roles in supporting the stronger Ar⋯Ar interactions, but the greater consistency for O⋯H is likely to be a result of the rotational flexibility of the benzyl alcohol arm. To investigate this, measurement of the oxygen atom position with respect to the plane of the C1–C6 aromatic ring was undertaken and is shown in Fig. 5 ▸.

Though all three compounds show some deviation from the plane of the aromatic ring, BBA-9-Me and BBA are clearly greater. This is likely to be due to the greater degree of movement that is possible for molecules where the O⋯H contact plays a more influential role in the stabilization of the structure. This allows BBA-9-Me and BBA to adopt more complementary O⋯H interactions with H⋯Ar to compensate for the lack of Ar⋯Ar contacts.

#### 3.1.5. Structure quality assessment

The overall effect of these varying contacts and motifs is highlighted by the changes in the average occupancy of guest molecules. BBA-9-Me possesses the lowest average occupancy (25.3%), which is significantly lower than BBA and BBA-3-Me (averages of 44.3 and 44.2%, respectively). The main disparity is likely to be caused by the differences in Ar⋯Ar contacts between analytes, which highlight the consequences of altering aromatic motifs. The almost identical average occupancies of BBA and BBA-3-Me are particularly interesting because of the variation of contributions to their interactions. The influence of this is visible in the average number of interactions, where BBA requires 25% more interactions to achieve the same occupancy as BBA-3-Me. This suggests BBA-3-Me adopts stronger contacts within the pore and emphasizes the greater number of H⋯Ar and O⋯H contacts required, compared with a slight increase in Ar⋯Ar. It also shows that with fewer Ar⋯Ar contacts there is a much greater reliance on alternative interactions, as observed for BBA-9-Me.

#### 3.1.6. Group summary

In a wider context, this group of molecules illustrates the direct influence that small sterically hindering substituents can impose on the interactions adopted. For BBA-3-Me, the proximity of the methyl group to the central aryl–aryl bond restricted rotational flexibility and led to more consistent adoption of favourable exchange sites within the pore. By contrast, the more flexible structures of BBA-9-Me and BBA occupy a greater variety of locations. It is also shown that substitution of these groups may not directly influence molecular conformation but can cause significant disruption to otherwise favourable interactions. For BBA-9-Me, the loss of Ar⋯Ar resulted in greater reliance on H⋯Ar and O⋯H, which was accompanied by increased movement of the benzyl alcohol arm. This shows that adsorbing molecules without alternative functionalities or conformational flexibility could be severely impaired by this loss of interaction and this is likely to impede the long-range ordering required for CS analysis.

### 3.2. Group 2

Group 2 contains BBA and BBA-10-CN, providing a direct comparison to understand the electronic influence of nitrile functionalities. Although a common functional group in organic chemistry, at the time of writing only 9 of the 400+ CS structures deposited in the Cambridge Structural Database (CSD) contain nitrile groups.^2^ These structures all exhibit significant distortion from ideal geometry and many require imposing substantial restraints during refinement. Increased fundamental understanding of the influence of this functional group will improve future characterization of these compounds in a similar fashion to the work conducted on N-containing heterocycles and aliphatic amines (Sakurai *et al.*, 2017 ▸).

**Figure 6:**
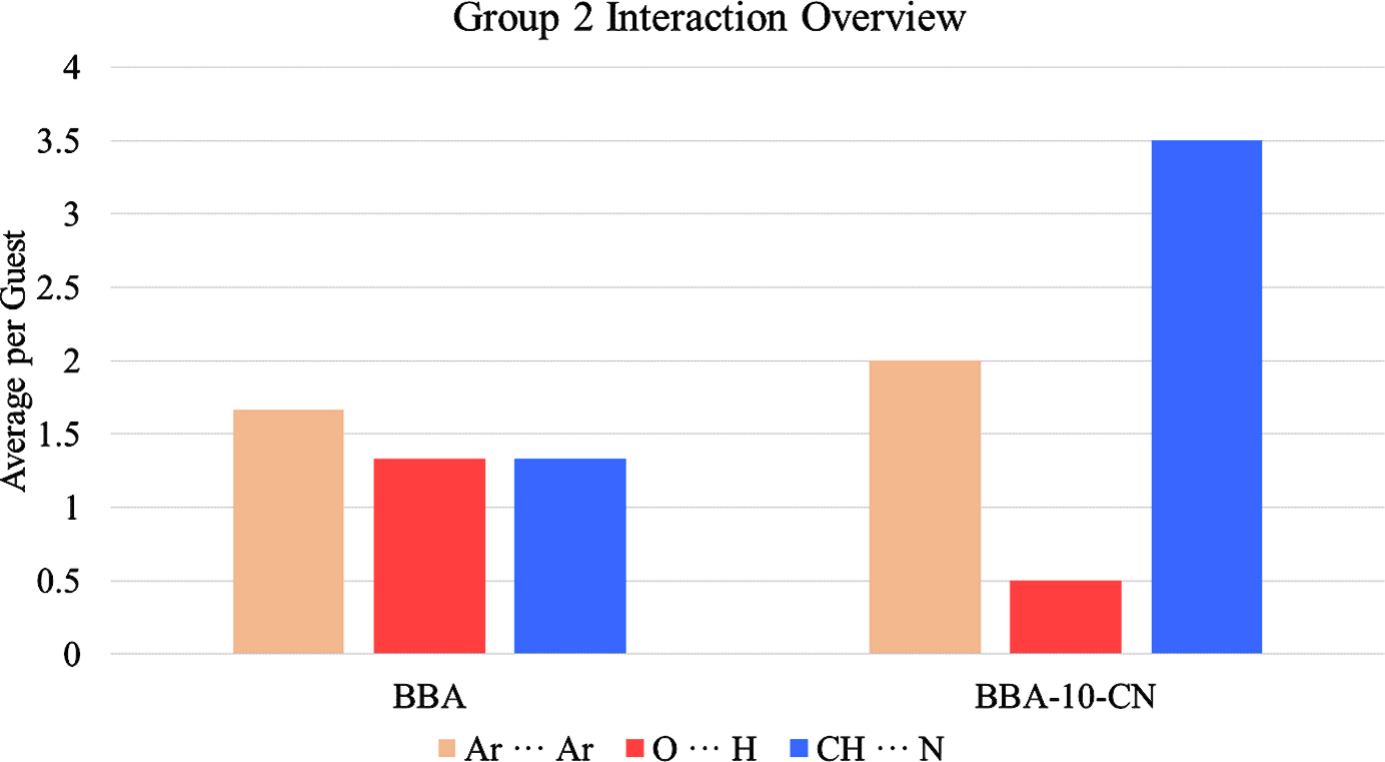
Average interactions per guest for BBA and BBA-10-CN.

To explore the effect of the nitrile group, the three main interactions investigated are Ar⋯Ar, O⋯H and CH⋯N. An overview of these contacts is illustrated in Fig. 6 ▸, and the accompanying group summary as well as a full list of interactions can be found in Sections S6 and S7 of the supporting information.

The most apparent difference is a significant increase in the number of CH⋯N interactions. The prevalence of this interaction for BBA-10-CN compared with any other molecule in the study would have been expected because it is the only compound that can act as both a donor and an acceptor. Consequently, BBA-10-CN participates in both host–guest and guest–guest CH⋯N interactions.

**Figure 7:**
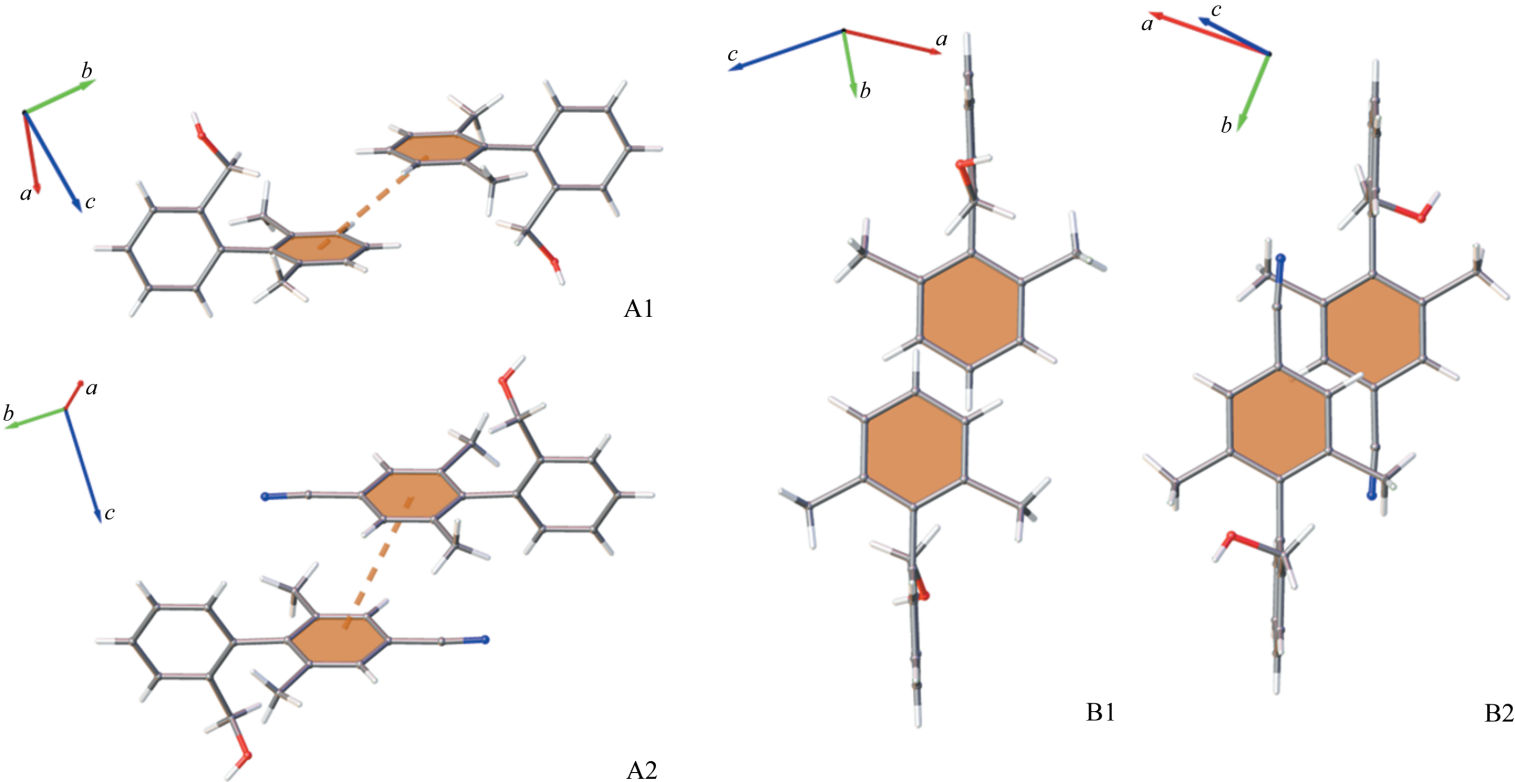
Orientation of BBA (A1) and BBA-10-CN (A2) rings and elongation/lateral shift of ring overlap for BBA (B2) and BBA-10-CN (B2) during the Ar⋯Ar interaction.

The increase in CH⋯N contacts is also accompanied by an increase in Ar⋯Ar interactions. This indicates that the nitrile group does not appear to limit adoption of Ar⋯Ar and therefore the electron-withdrawing resonance effects that reduce aromatic character do not initially appear to play a significant role. However, like in Group 1, the changes in the molecular structure result in considerably different aromatic motifs adopted by guest molecules, as shown in Fig. 7 ▸.

The major difference between guest–guest interactions observed for Group 2 compounds is the location of the benzyl alcohol arm relative to the Ar⋯Ar interaction of the C7–C12 rings. In BBA the substituent is directed downwards on the same side as the central interaction, whereas in BBA-10-CN the groups are oriented away. Two main factors drive this change in motif: steric repulsion between the benzyl alcohol arm/nitrile group and satisfying the electronic properties of the nitrile group. The impact of this difference in motifs is that BBA-10-CN can still adopt Ar⋯Ar interactions with both C1–C6 and C7–C12 rings rather than losing potential contacts, as observed for BBA-9-Me in Group 1.

Another difference between the two is the direction of ring slippage. For BBA this results in elongation of the interaction and is likely to be adopted to minimize steric repulsion. Conversely, BBA-10-CN has a more laterally shifted overlap but still results in similar centroid–centroid distances to BBA. This benefits the formation of guest–guest CH⋯N contacts for BBA-10-CN, while achieving similar ring separation to maximize aromatic interaction.

The final difference between Group 2 compounds is the decrease in the number of O⋯H interactions for BBA-10-CN. Investigation of the oxygen position away from the ring planes in BBA-10-CN shows a deviation of 84 (5)° to adopt the only O⋯H contact observed. This demonstrates that the molecule can utilize its conformational flexibility to gain more interactions, even when adopting the different Ar⋯Ar motif. Interestingly, unlike H⋯Ar and O⋯H in Group 1, the O⋯H and CH⋯N interactions observed in Group 2 do not appear to operate in a synergistic manner and instead compete. This further highlights the indirect effect imposed by the functionalization of aromatic rings on the interactions adopted.

Adopting this range of interactions results in a significant difference in average guest occupancy: BBA averages 44.3%, whereas BBA-10-CN averages 27.2%. The low occupancy of BBA-10-CN also necessitates the application of considerable restraints in modelling and is accompanied by a relatively high amount of residual electron density. The average number of interactions also provides insight into the overall strength of the contacts present, with both compounds relying on a similar average per guest, and BBA-10-CN is assumed to be much more weakly interacting.

Group 2 has provided another example of nitrile-containing compounds resulting in a poorly defined CS structure. It is thought that the electronic environment desired by the nitrile group, as well as its size and rigidity, play significant roles and affect the exchange locations and motifs adopted by the guests. In BBA-10-CN it is suggested that, although the local ordering with guest–guest interaction is satisfied, this can have detrimental effects on the regular long-range ordering.

### 3.3. Group 3

Group 3, and subsequently Group 4 and Group 5, provide an opportunity to compare PBA and BBA systems. The presence of a phenol group will introduce electron-withdrawing inductive (−I) and electron-donating mesomeric (+M) effects which influence the aromatic character of the C1–C6 ring. The consequence of this is not clear; the overall result is a more electron-rich π-system which will favour interaction with the electron-deficient framework, but it also increases the negative quadrupole associated with the aromatic ring which will disfavours Ar⋯Ar interaction (Hunter & Sanders, 1990 ▸). Additionally, the reduced conformational flexibility available for the O⋯H interaction will likely impact either the number of hydrogen contacts adopted or may induce a greater change in ring torsion angles.

**Figure 8:**
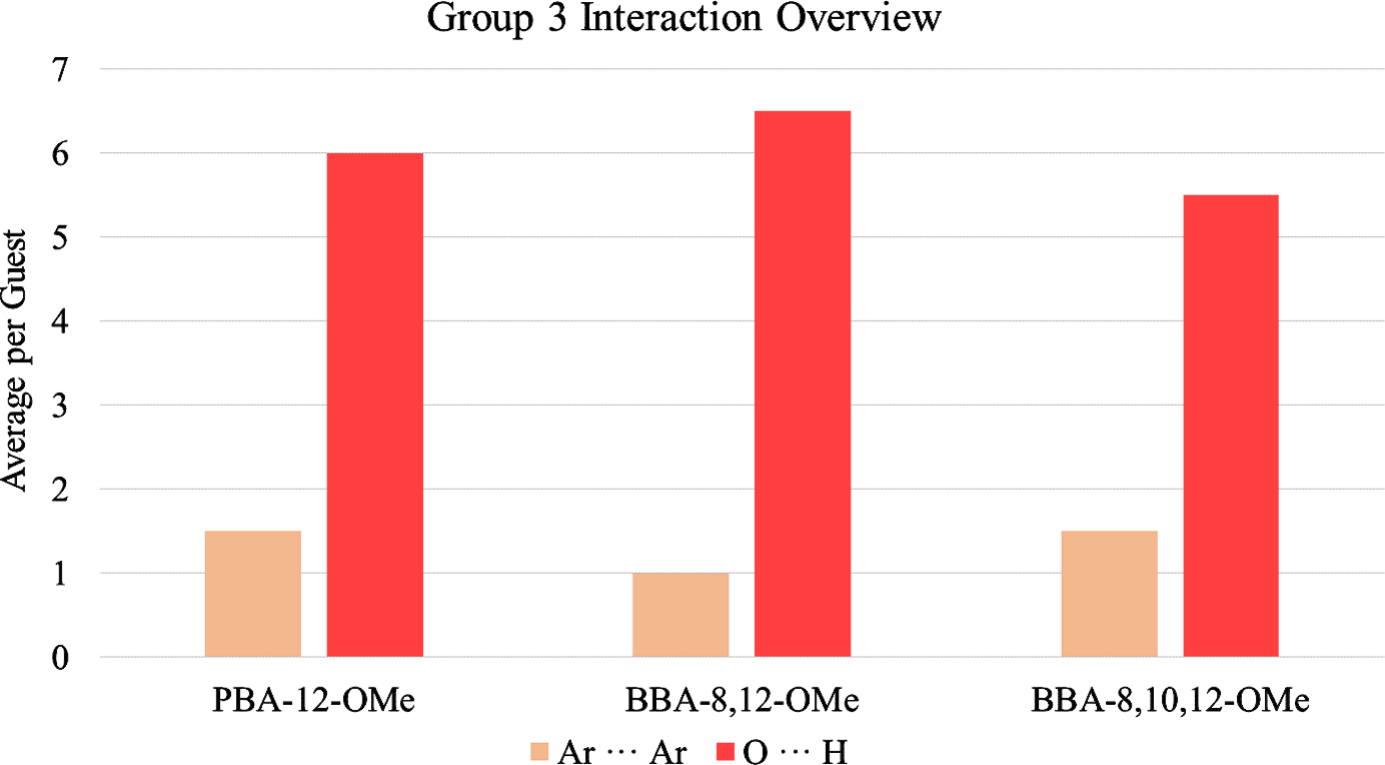
Average interactions per guest for PBA-12-OMe, BBA-8,12-OMe and BBA-8,10,12-OMe.

Group 3 contains PBA-12-OMe, BBA-8,12-OMe and BBA-8,10,12-OMe. Functionalization with meth­oxy groups allows exploration of steric and electronic properties. The molecules discussed here have variations in the number of substitutions and therefore provide insight into the interplay between these two factors. The two main interactions are Ar⋯Ar and O⋯H. The average number of interactions per analyte are illustrated in Fig. 8 ▸, while accompanying full lists of interactions and a detailed summary can be found in Sections S6 and S7 of the supporting information.

The dominant interaction in all three molecules is O⋯H, which is likely due to the presence of numerous oxygen atoms in each compound. However, there is not a direct relationship between the number of oxygen atoms in a molecule and the number of O⋯H contacts observed. The interaction contributions from hydroxyl or meth­oxy groups and whether they occur between host and guest or guest and guest is shown in Table 1 ▸. Full tabulation of O⋯H interactions for Group 3 can be found in Section S7.1 and Table S48 of the supporting information.

Interestingly, PBA-12-OMe has the greatest contribution from the hydroxyl oxygen, which suggests that the conformational flexibility of the benzyl alcohol arm for the BBA structures is not as influential as the presence of multiple meth­oxy groups. Notably, the additional meth­oxy functionalities of the BBAs do not lead to a significant percentage contribution, even when substituted at a position which is less sterically hindered for intermolecular interaction.

Evaluation of preference for host–guest or guest–guest interactions highlights that the BBAs rely on the host framework for the majority of O⋯H contacts. This is likely to be a consequence of greater steric bulk which prevents the BBAs from maximizing host–guest and guest–guest interactions simultaneously, whereas PBA-12-OMe can adopt an equal mix of concurrent interactions with neighbouring guests and the host framework.

**Figure 9:**
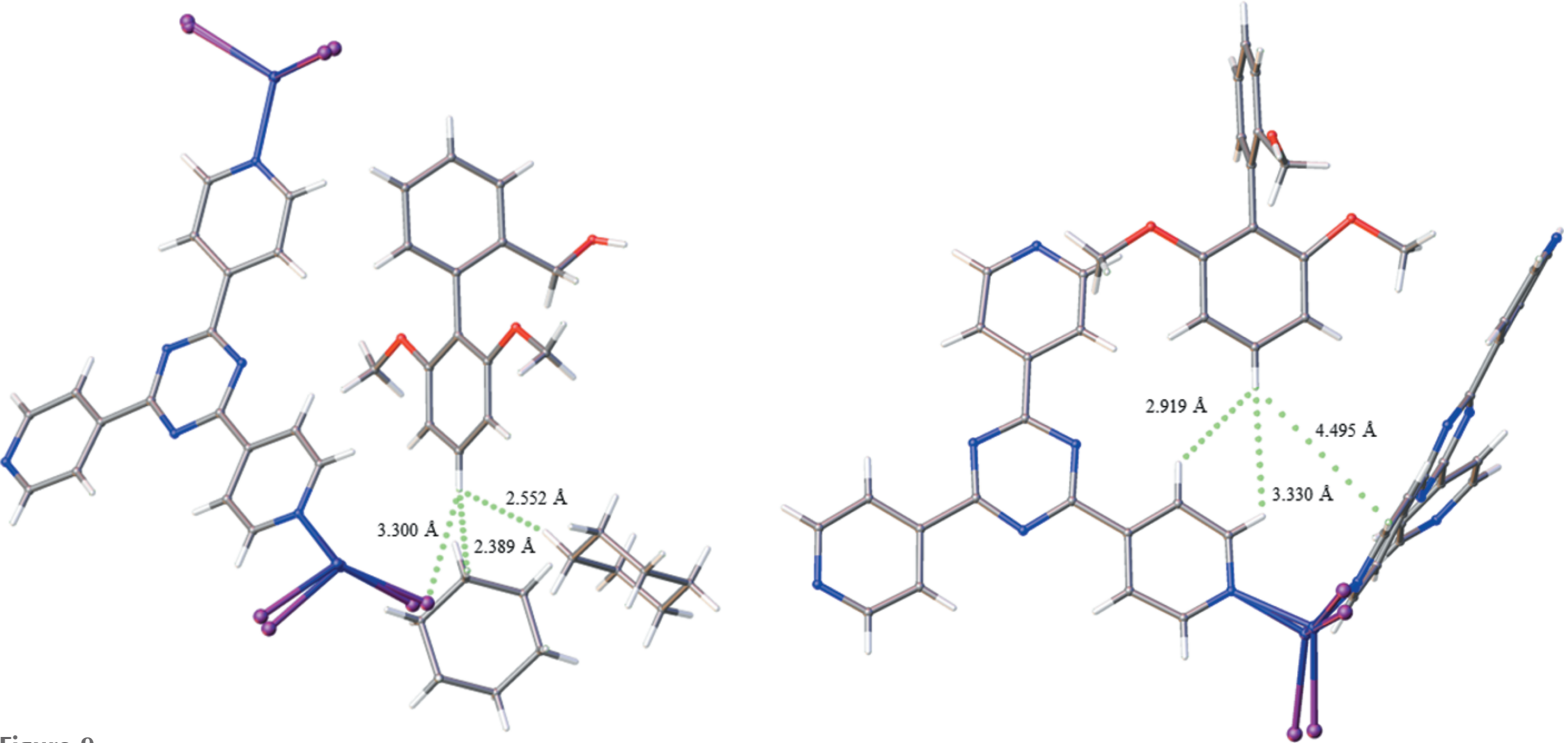
Close contacts for atom C10 of BBA-8,12-OMe for exchange sites A (left) and B (right).

This results in PBA-12-OMe adopting a similar number of O⋯H interactions to BBA-8,12-OMe, even though it has fewer oxygen atoms available. The preference of BBA-8,12-OMe for host–guest, instead of guest–guest, interactions is likely to be due to a more complementary molecular shape and electron-deficient aromatic rings. Further consideration of steric bulk enables rationalization of the decrease in O⋯H interactions from BBA-8,12-OMe to BBA-8,10,12-OMe. Close contacts within the pore for BBA-8,12-OMe are visualized in Fig. 9 ▸.

The proximity of framework atoms to the C10 position of BBA-8,12-OMe demonstrates the enclosed space, and hence the reduced volume for optimizing interaction, occupied by guests and therefore the preferred locations are adopted. For BBA-8,10,12-OMe this location would be unsuitable because of the steric repulsion introduced by an additional meth­oxy group. Instead, BBA-8,10,12-OMe must compromise and adopt a new position which is more complementary to its size. The consequence is twofold, with an overall decrease in O⋯H interactions and a greater reliance on guest–guest interactions.

In contrast to both the O⋯H contacts and the trends in Group 1 and Group 2, Ar⋯Ar interactions are not a significant interaction for Group 3. All three guests adopt a similar number of Ar⋯Ar interactions; however, it was previously posited that the phenol hydroxyl group may influence this with its contribution to a more electron-rich π-system. Although not prominent for Group 3, further investigation of the Ar⋯Ar motifs provides insight into the steric demands of the varying functionalization and accordingly the ring slippage for each Ar⋯Ar interaction is given in Table 2 ▸.

PBA-12-OMe exhibits the closest overlap compared with both BBAs. This is consistent with the hypothesis that smaller steric bulk enables closer packing both to the host and with other guests in the pore. Interestingly, the B exchange site of BBA-8,10,12-OMe possesses one interaction with much closer ring overlap than either PBA-12-OMe or BBA-8,12-OMe. This showcases the versatility of the guests to adopt different interactions and motifs, however it requires sacrifice of O⋯H contacts to achieve this. This is shown by the A exchange site, which possesses 7× more O⋯H interactions than the B site as a result of more distant aromatic interactions and demonstrates the incompatibility of the interactions for this group of molecules.

The influence of these interactions results in PBA-12-OMe possessing the lowest average occupancy (29.9%), whereas BBA-8,10,12-OMe has the second highest (41.8%) and BBA-8,12-OMe has the highest (47.5%). This suggests that the more diverse host–guest and guest–guest interactions of PBA-12-OMe lead to weaker contact. The average number of interactions follows an inverse trend to occupancy, which further suggests that PBA-12-OMe is more weakly interacting. For BBA-8,12-OMe the greatest average occupancy and fewest average interactions may be aided by the significant preference for host–guest interaction, which can offer more reliable support than neighbouring guests.

Group 3 highlights the interplay between steric and electronic properties that must be considered for bulky groups with a propensity for hydrogen bonding. This also emphasizes the ‘induced fit’ which guest molecules adopt when encapsulated within the host framework and the influence this has on the subsequent interactions adopted. The best example of this is shown by comparison of A and B exchange sites in BBA-8,10,12-OMe, which rely on O⋯H and Ar⋯Ar, respectively, but produce similar models. This showcases the versatile modes of interaction available for the same molecules and the equal level of stabilization that can be achieved.

### 3.4. Group 4

Group 4 contains PBA, PBA-3-F, PBA-2-F and PBA-2,6-I. This set of molecules examines the influence of halogen atoms on the aromatic and oxygen-based interactions that were dominant in previous groups. A comparable size to hydrogen, together with its strongly electron-withdrawing nature, makes fluorine an attractive and commonly used functionalization in organic chemistry. Therefore, for the widest possible adoption of the CS method, understanding the electronic effect of fluorine is crucial. Iodine is significantly less electron-withdrawing but is commonly found in aromatic intermediates to facilitate the addition of organic and heteroatom substituents. Further understanding its influence would assist in the characterization of such intermediates in a manner akin to the approach used for drug metabolites (Rosenberger *et al.*, 2020 ▸).

**Figure 10:**
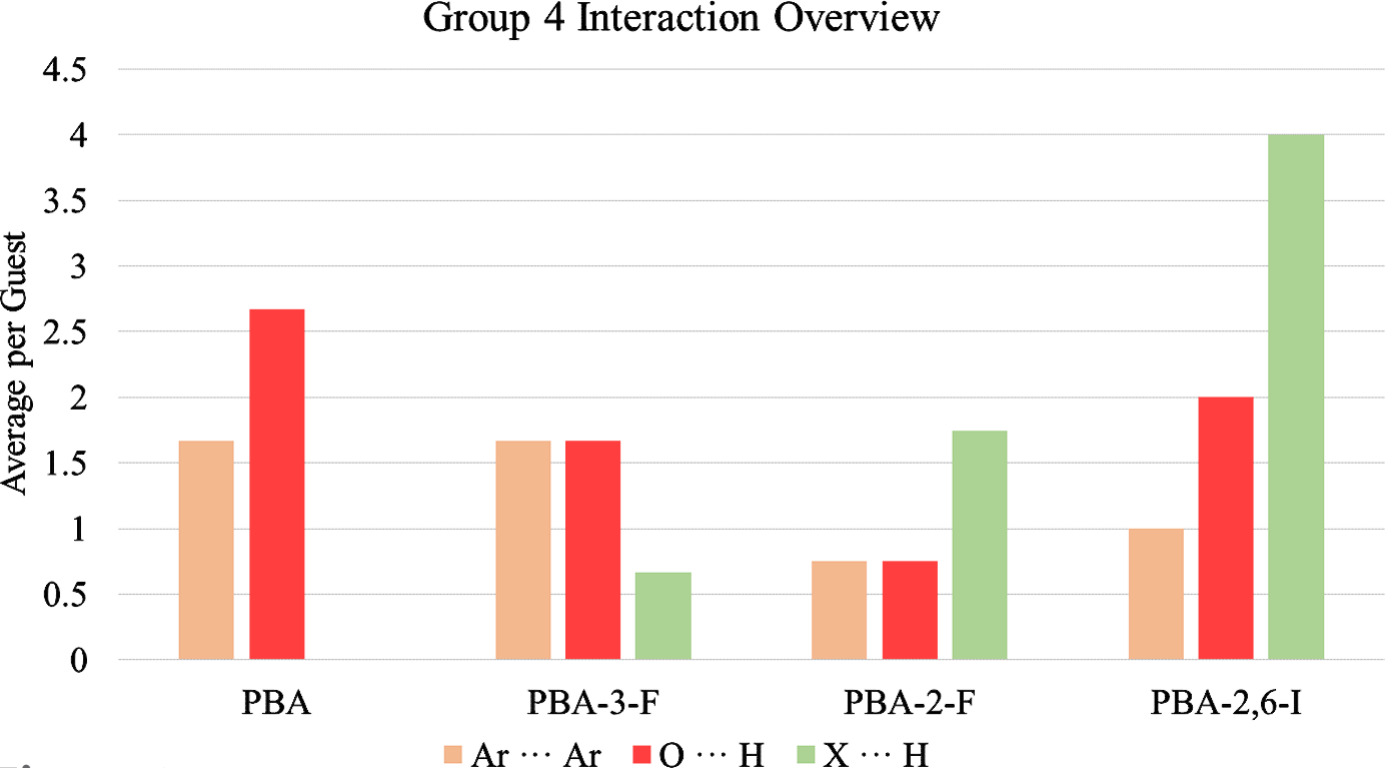
Average interactions per guest for PBA, PBA-3-F, PBA-2-F, PBA-2,6-I.

The main interactions investigated in this group are Ar⋯Ar, O⋯H and halogen⋯hydrogen (X–H). The average interaction summary for the series is illustrated in Fig. 10 ▸ and the accompanying full interaction tables and a group summary are given in Sections S6 and S7 of the supporting information.

**Figure 11:**
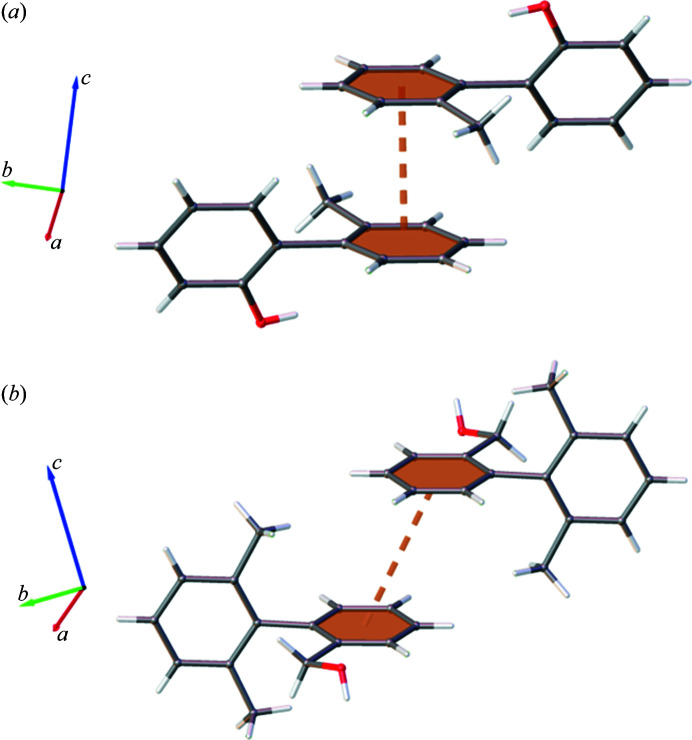
Example Ar⋯Ar interactions with aromatic ring preference for (*a*) PBA and (*b*) BBA.

Investigation of PBA enables comparison with the BBA compound discussed in Group 1 and Group 2. Both molecules represent the simplest, or least functionalized, structures of their respective groups. They possess similar averages for the number of Ar⋯Ar contacts, 1.50 and 1.67 per guest, respectively, but this similarity is not reflected in the extent to which a particular ring is involved in the interaction. PBA has a significant preference for the C7–C12 ring with it being involved in 77.8% of its Ar⋯Ar interactions, compared with 40.0% for BBA. Exemplar interactions are shown in Fig. 11 ▸. This suggests that the phenol group does not impair aromatic interaction overall but discourages contact with the more electron-rich C1–C6 ring.

In Group 4, Ar⋯Ar interactions do not exhibit a clear trend across the series. The −I nature of fluorine appears to have a varied effect, with no change in Ar⋯Ar contact between PBA and PBA-3-F but a significant decrease for PBA-2-F. PBA-2,6-I also experiences fewer aromatic interactions than PBA-3-F, even though the substituents are much less electronegative. The lack of distinguishable trends suggests a more complex influence of the halogens than simply altering the properties of the aromatic rings. These observations cannot currently be rationalized and require more attention in subsequent research.

Further investigation of the halogen influence requires consideration of O⋯H and X⋯H interactions. The steady decrease in O⋯H from PBA to PBA-2-F corresponds with an increase in X⋯H. Therefore, it can be inferred that there is competition for interaction between the two functionalities, as with the non-complementary O⋯H and CH⋯N interactions in Group 2. For PBA-3-F, which has symmetric substitution of the *ortho*-position, the hydroxyl group is expected to dominate over fluorine in the competition for interactions because it has a stronger hydrogen-bonding ability (Dunitz & Taylor, 1997 ▸). These groups possess an identical number of electrons which makes them almost impossible to differentiate for low-occupancy exchange sites within a framework with heavy atoms. This is discussed in greater detail in Section S6.2 of the supporting information as a procedure which provides greater confidence in atom assignment for CS structures.

The change of substitution position for PBA-2-F results in an increase in X⋯H interactions, which become more prevalent than the O⋯H interaction. This can be rationalized as the fluorine is now located further away from the sterically crowded aryl–aryl bond and thus is less inhibited for interaction compared with the hydroxyl group (interestingly, this is not observed in Group 3 which is ascribed to varying exchange locations caused by the size of the meth­oxy groups).

**Figure 12:**
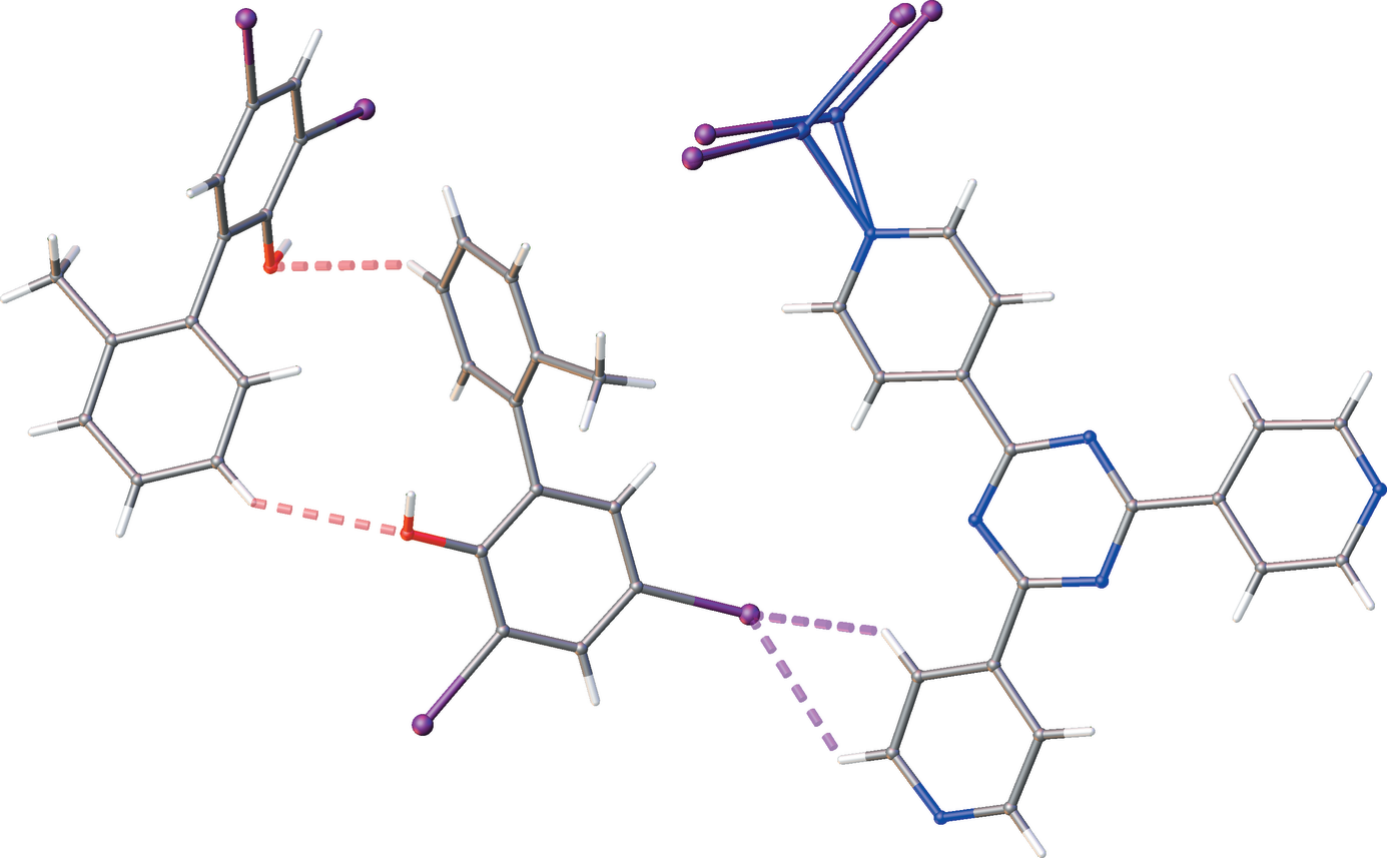
Co-operative oxygen- and iodine-based interactions for PBA-2,6-I.

Finally, there are twice as many X⋯H interactions for PBA-2,6-I compared with PBA-2-F, suggesting that the interaction may scale linearly with an increasing number of halogen substituents. This is also dissimilar to Group 3 which showcased a more complex trend when increasing the number of meth­oxy groups and is proposed to be caused by the introduction of steric bulk. The major contribution from X⋯H is also accompanied by a significant number of O⋯H interactions. It is proposed that the location of substitution and longer C—I bonds enable a more co-operative action between the two contacts, which then benefits the guest stability. These interactions are shown in Fig. 12 ▸.

The overall influence of the varying interactions on the molecular structure determination of PBA, PBA-3-F and PBA-2-F is minimal, as the three molecules achieve similar average guest occupancies of 26.8, 21.0 and 23.5% respectively. There is a slight decrease in the average number of interactions across this series, which suggests that PBA-2-F may adopt the strongest contacts. One of the clearest differences between these compounds is the number of exchange sites (6, 4 and 3, respectively). This hints that the halogen atoms cause intermolecular interactions to become more directional and specific. Therefore, although there are numerous locations which suit the size and shape of the molecules, a limited number have the requisite interactions available for regular ordering throughout the crystal. This hypothesis is supported by PBA-2,6-I which has two exchange sites and a significant increase in average occupancy to 74.7%. This large increase only requires a few more interactions for PBA-2,6-I than PBA which again suggests that the introduction of halogens can improve the strength of host–guest and guest–guest contacts.

This series of molecules demonstrates that halogen atoms can adopt interactions within the pores and therefore have a more direct influence than just altering the electronic properties of the corresponding aromatic rings. This highlights the relative freedom which guest molecules possess in the framework, as they can change orientation to satisfy these different functionalities. The influence of halogen interactions is greatly affected by three factors. First, the element type, where poorly hydrogen bonding fluorine is not as effective as larger halogens which have better orbital overlap for interaction. Second, substituent position, where substitution away from the crowded central aryl–aryl bond allows for better intermolecular contact. Finally, the number of atoms, where more halogen atoms improve the number of directions which can be utilized for interaction within the enclosed environment.

### 3.5. Group 5

Group 5 contains PBA, PBA-2-Me and PBA-2-Ph. This series explores functionalization with increasingly bulky substituents away from the central aryl–aryl bond. The additional aromatic ring in PBA-2-Ph also provides insight into whether molecules with more aromatic groups have greater stability in the CS pore.

**Figure 13:**
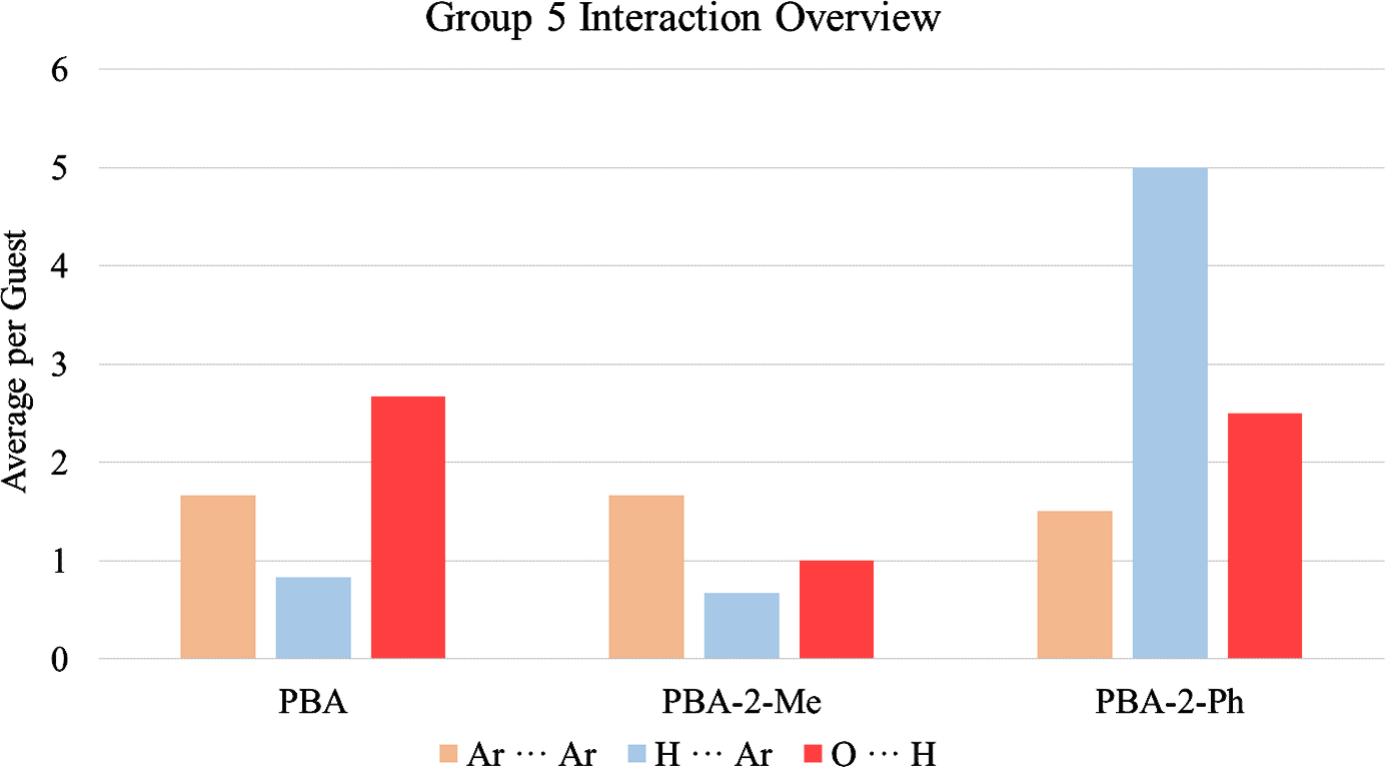
Average interactions per guest for PBA, PBA-2-Me and PBA-2-Ph.

To investigate the influence of this functionalization, Ar⋯Ar, H⋯Ar and O⋯H interactions are examined. The average interaction per guest across the series is illustrated in Fig. 13 ▸ and additional tabulation is provided in Sections S6 and S7 of the supporting information.

**Figure 14:**
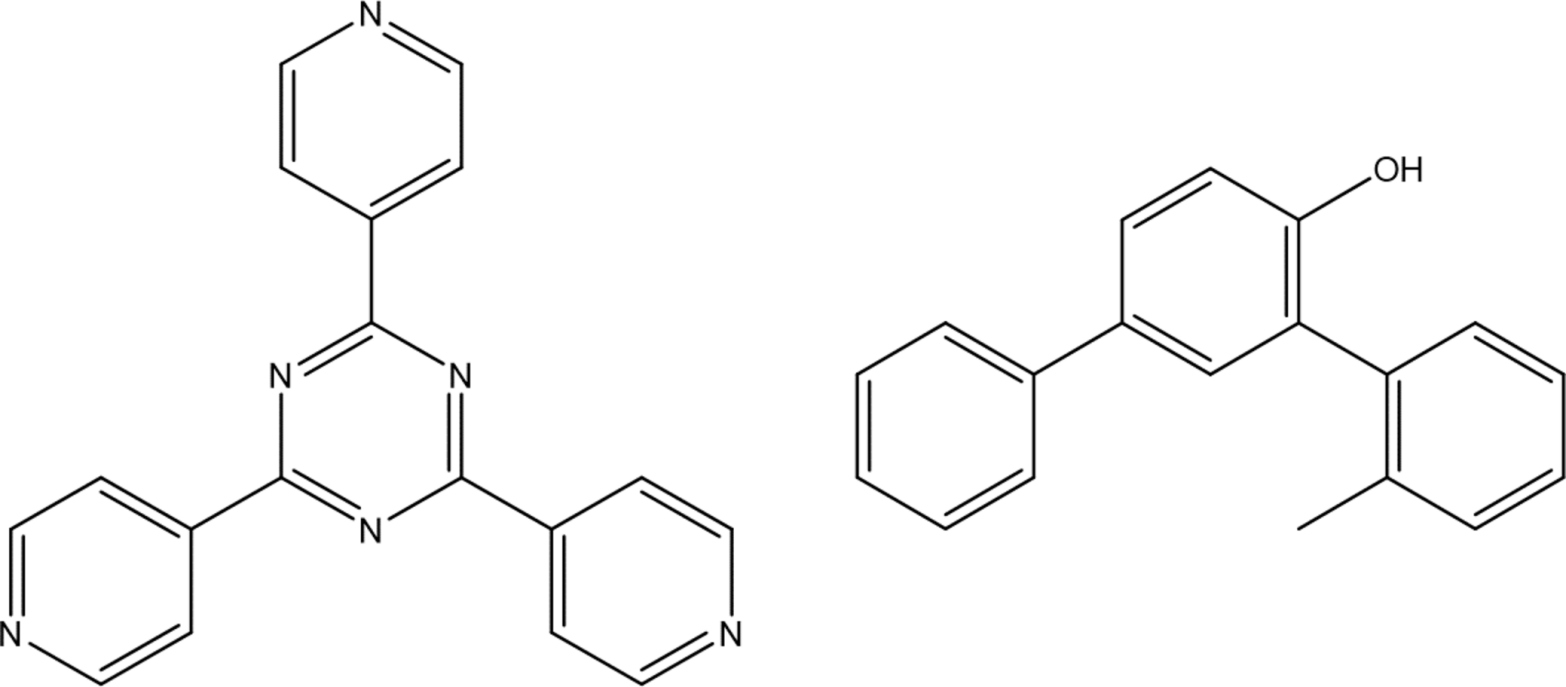
Complementary 2D representations of tpt and PBA-2-Ph.

There is minimal variance in Ar⋯Ar interactions across the series, with PBA and PBA-2-Me having very similar structures and no distinct electronic differences. However, the slight decrease observed for PBA-2-Ph is unexpected because of the significant role of the Ar⋯Ar contacts, as indicated throughout this study, and the apparent molecular shape complementarity with the tpt linker, as illustrated in Fig. 14 ▸.

**Figure 15:**
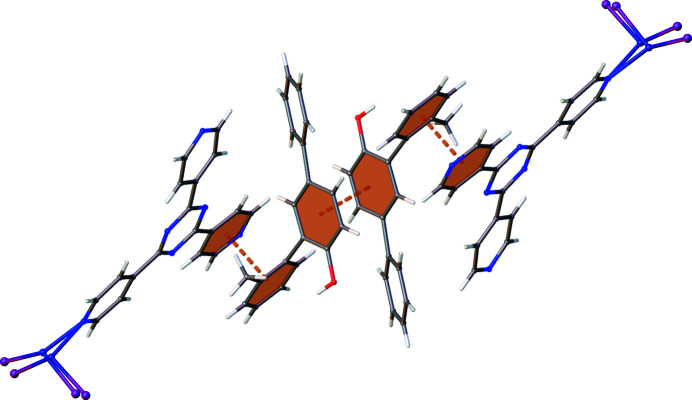
PBA-2-Ph clustered host–guest and guest–guest aromatic interactions

The lack of increase in Ar⋯Ar in PBA-2-Ph can be rationalized by assessing the 3D conformation of the compound in relation to the space available within the framework. Steric repulsion between hydroxyl and methyl groups prevents a co-planar orientation of C1–C6 and C7–C12 rings. This induced twist would minimize intramolecular steric repulsion, but results in an unfavourable alignment for interaction with the planar tpt ligand. Instead, the guest molecule can adopt some overlap with the framework linker, while also utilizing the freedom of the cavity, as shown in Fig. 15 ▸.

The resulting sandwich-like cluster enables contact between the electron-rich C7–C12 guest ring and the electron-deficient framework. Simultaneously, the C1–C6 ring sits in the middle of the pore, which allows interaction with a symmetry-related molecule. Notably, Ar⋯Ar interactions are not adopted by the C13–C18 ring of the A exchange site, or any rings of the B exchange site for PBA-2-Ph. Instead, these rely on H⋯Ar interaction for stability. This reliance is reflected in a significant contribution to interactions for PBA-2-Ph in comparison with PBA and PBA-2-Me. This highlights the versatility of interaction within the pore and the varied action of aromatic rings, which makes them well suited to the CS method. In contrast, PBA-2-Me possesses the fewest H⋯Ar interactions, albeit a minimal decrease from PBA, but this is an initial indicator of the disruption that can be caused through addition of sterically dominating groups.

**Figure 16:**
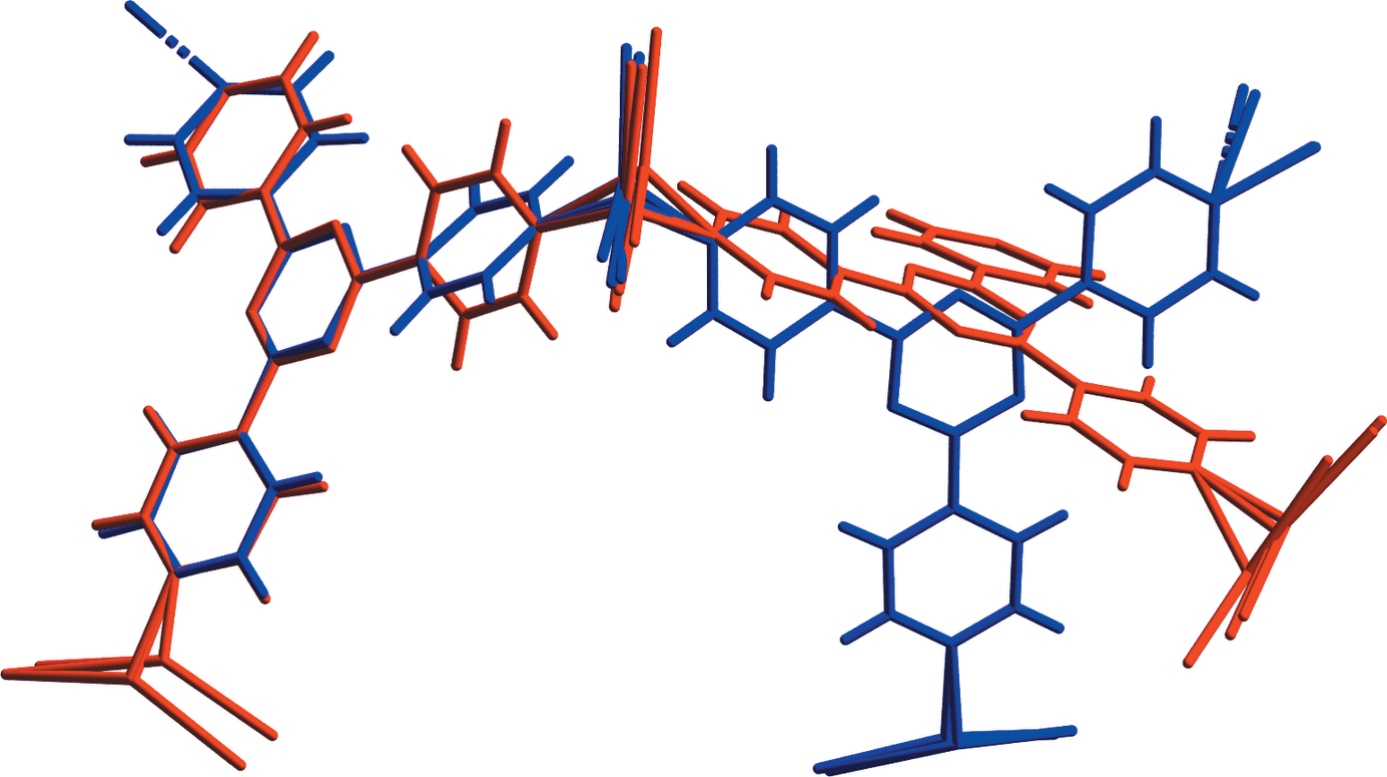
Comparison of the host framework conformation for PBA (blue) and PBA-2-Me (red). Guest molecules have been omitted for clarity.

This disruptive effect is revealed through investigation of O⋯H interactions and their reduction observed between PBA and PBA-2-Me. Investigation of H2 and H3 atoms of PBA and C15 of PBA-2-Me highlights the change in environments. There is an overall loss of three O⋯H interactions when comparing PBA with PBA-2-Me, which are replaced by a single H⋯Ar contact. This is likely related to a change in guest location because the additional methyl of PBA-2-Me would have considerable steric repulsion. Overlay of the two structures reveals a significant difference in host framework conformation, illustrated in Fig. 16 ▸.

Although this distortion prevents direct comparison of the exchange sites adopted, it clearly highlights the change of environment brought about by the additional methyl group of PBA-2-Me. Comparatively, PBA-2-Ph regains some O⋯H interactions but not to the same extent as PBA. This suggests that the location adopted by PBA-2-Ph achieves a better balance between the three principal interactions.

Variation of intermolecular interactions has a negligible influence on the average guest occupancy for PBA, PBA-2-Me and PBA-2-Ph in Group 5 (26.8, 24.9 and 26.7%, respectively). Although differences in O⋯H interactions were identified between PBA and PBA-2-Me, they both achieve comparable average guest occupancies with a similar number of interactions. This may be aided by the more restricted torsion angle observed for PBA-2-Me, which allows it to adopt two exchange sites which maximize the interaction, as previously noted for BBA-3-Me. In comparison, PBA-2-Ph experiences a significant increase in the average number of interactions because of the greater reliance on weaker H⋯Ar contacts. A comparison between PBA-2-Ph and BBA-8,10,12-OMe (Group 3) shows that the two exchange sites in the asymmetric unit can rely on different interactions but result in similar guest occupancies and accompanying restraints. This also highlights the cumulative effect of many weaker interactions which can be utilized to match the stronger contacts.

The series also demonstrates the impact that relatively small structural changes can impose on the location adopted by guests within a pore, although in this case the variation in guest exchange sites did not have a significant influence on the overall molecular structure elucidation. It has also been shown that additional aromatic rings do not have a direct correlation with the number of Ar⋯Ar contacts adopted. Instead, the substitution position, which is greatly influenced by the steric bulk of the molecule, and the electronic properties of the accompanying rings play an important role as to which interaction is the most favourable.

## 4. Conclusions and future work

Drawing from the summaries of each of the five groups analysed, it is shown that the location of the guest exchange site is determined by the steric requirements of the molecule. The subsequent orientation and molecular conformation of the guest are governed by the intermolecular interactions available at the exchange site. The interactions adopted will then define the ability of guests to achieve regular order and influence the quality of the resolved structure.

Accordingly, structurally related compounds can exhibit considerably different behaviours. In particular, the addition or rearrangement of functionalities which have been shown to aid guest stability may actually have a destabilizing effect if not considered in conjunction with the 3D shape and conformation of guest molecules.

It is also suggested that some functional groups favour localized interaction rather than regular long-range ordering, as introduced in the evaluation of nitrile functionalities in Group 2. This may manifest itself as guest–guest interactions which appear relatively strong, but the accompanying host–guest contacts are weak. The resulting CS structure may only be poorly resolved because of the lack of consistent stabilization throughout cavities in the framework.

Follow-on investigations from this work will use crystallographic databases to identify functional group propensity to form strong intermolecular interactions with similar groups. This will provide insight into which functionalities are more likely to form strong guest–guest interactions and therefore may be more successfully analysed with other CS variants or different advanced crystallization methods (Metherall *et al.*, 2023 ▸). This work will also consider the influence of host distortion which has already been shown to vary greatly depending on the guest exchanged within the framework.

Finally, further systematic studies will be undertaken in conjunction with computational simulation and solution state analysis to improve our understanding of conformational restraints imposed by encapsulation within the framework. The PBA and BBA structures provide the ideal platform for this work due to their structural simplicity and readily quantifiable molecular shape.

## 5. Related literature

The following references are cited in the supporting information: Zigon *et al.* (2015 ▸); Sheldrick (2015 ▸); Spek (2003 ▸); Ramadhar *et al.* (2015 ▸); Groom *et al.* (2016 ▸); Biradha *et al* (2002 ▸); Dolomanov *et al.* (2009 ▸).

## Supplementary Material

Crystal structure: contains datablock(s) BBA-9-Methyl_CrystallineSponge, BBA_CrystallineSponge, BBA-3-Methyl_CrystallineSponge, BBA-10-Nitrile_CrystallineSponge, PBA-12-Methoxy_CrystallineSponge, BBA-8_12-Methoxy_CrystallineSponge, BBA-8_10_12-Methoxy_CrystallineSponge, PBA_CrystallineSponge, PBA-3-Fluorine_CrystallineSponge, PBA-2-Fluorine_CrystallineSponge, PBA-2_6-Iodine_CrystallineSponge, PBA-2-Methyl_CrystallineSponge, PBA-2-Phenyl_CrystallineSponge. DOI: 10.1107/S2052252523005146/lt5059sup1.cif

Structure factors: contains datablock(s) BBA_CrystallineSponge. DOI: 10.1107/S2052252523005146/lt5059BBA_CrystallineSpongesup2.hkl

Structure factors: contains datablock(s) BBA-3-Methyl_CrystallineSponge. DOI: 10.1107/S2052252523005146/lt5059BBA-3-Methyl_CrystallineSpongesup3.hkl

Structure factors: contains datablock(s) BBA-8_10_12-Methoxy_CrystallineSponge. DOI: 10.1107/S2052252523005146/lt5059BBA-8_10_12-Methoxy_CrystallineSpongesup4.hkl

Structure factors: contains datablock(s) BBA-8_12-Methoxy_CrystallineSponge. DOI: 10.1107/S2052252523005146/lt5059BBA-8_12-Methoxy_CrystallineSpongesup5.hkl

Structure factors: contains datablock(s) BBA-9-Methyl_CrystallineSponge. DOI: 10.1107/S2052252523005146/lt5059BBA-9-Methyl_CrystallineSpongesup6.hkl

Structure factors: contains datablock(s) BBA-10-Nitrile_CrystallineSponge. DOI: 10.1107/S2052252523005146/lt5059BBA-10-Nitrile_CrystallineSpongesup7.hkl

Structure factors: contains datablock(s) PBA_CrystallineSponge. DOI: 10.1107/S2052252523005146/lt5059PBA_CrystallineSpongesup8.hkl

Structure factors: contains datablock(s) PBA-2_6-Iodine_CrystallineSponge. DOI: 10.1107/S2052252523005146/lt5059PBA-2_6-Iodine_CrystallineSpongesup9.hkl

Structure factors: contains datablock(s) PBA-2-Fluorine_CrystallineSponge. DOI: 10.1107/S2052252523005146/lt5059PBA-2-Fluorine_CrystallineSpongesup10.hkl

Structure factors: contains datablock(s) PBA-2-Methyl_CrystallineSponge. DOI: 10.1107/S2052252523005146/lt5059PBA-2-Methyl_CrystallineSpongesup11.hkl

Structure factors: contains datablock(s) PBA-2-Phenyl_CrystallineSponge. DOI: 10.1107/S2052252523005146/lt5059PBA-2-Phenyl_CrystallineSpongesup12.hkl

Structure factors: contains datablock(s) PBA-3-Fluorine_CrystallineSponge. DOI: 10.1107/S2052252523005146/lt5059PBA-3-Fluorine_CrystallineSpongesup13.hkl

Structure factors: contains datablock(s) PBA-12-Methoxy_CrystallineSponge. DOI: 10.1107/S2052252523005146/lt5059PBA-12-Methoxy_CrystallineSpongesup14.hkl

Supporting figures and tables. DOI: 10.1107/S2052252523005146/lt5059sup15.pdf

CCDC references: 2254202, 2254203, 2254204, 2254205, 2254206, 2254207, 2254208, 2254209, 2254210, 2254211, 2254212, 2254213, 2254214

## Figures and Tables

**Table 1:** Summary of Group 3 O⋯H interactions

	O⋯H (hydroxyl) (%)	O⋯H (meth­oxy) (%)	O⋯H (host–guest) (%)	O⋯H (guest–guest) (%)
PBA-12-OMe	50.00	50.00	53.85	46.15
BBA-8,12-OMe	38.46	61.54	92.31	7.69
BBA-8,10,12-OMe	45.45	54.55	72.73	27.27

**Table 2:** Comparison of ring slippage for Group 3 guests which adopt an Ar⋯Ar interaction

Compound	Ring I	Ring J	Distance between Cg(I) and perpendicular projection of Cg(J) on ring I (Å)
PBA-12-OMe	C1A–C6A	C333–C336	1.340
	C7A–C12A	C306–C308	2.740
	C7A–C12A	C7B–C12B	1.682
BBA-8,12-OMe	C1A–C6A	C1A–C6A	4.360
	C7A–C12A	C7A–C12A	3.457
BBA-8,10,12-OMe	C7B–C12B	C301–C305	3.335
	C7B–C12B	C306–C308	0.999
	C7B–C12B	C314–C314	4.424

Cg(I)/Cg(J) refer to the centre of gravity for rings I and J.

## Footnotes

1 Demonstrated by work within our group, which generated a CSD subset for CS structures, akin to that of the MOF subset available from the CCDC (Moghadam *et al.*, 2017 ▸).

2 CSD refcodes: LUDVEL, LUDVIP, RIYBAE, RIYBIM, UDAXIJ, ULOKAJ, ULOMAL, VAMLAZ, ZUBFAG, ZUCXON.
