# Supplementary material for: A systematic study of the interplay between guest molecule structure and intermolecular interactions in crystalline sponges
Source: IUCrJ. 2023 Jul 1;10(Pt 4):497–508. doi: 10.1107/S2052252523005146 (PMC10324488; doi:10.1107/S2052252523005146)
Supplement: Supplementary file 15 [file m-10-00497-sup15.pdf]

IUCrJ

Volume 10 (2023)

Supporting information for article:

A systematic study of the interplay between guest molecule structure and intermolecular interactions in crystalline sponges

Robert C. Carroll, David C. Harrowven, James E. Pearce and Simon J. Coles

Table of Contents

S1. Reagents, equipment, data collection, and refinement strategies 2

S2. Interaction criteria and identification 3

S3. Experimental conditions 5

S4. Molecular similarity table 6

S5. Crystallographic tables, notes, and models 7

S6. Full lists of intermolecular interactions 33

S7. Group interaction summaries and additional analysis 54

S7.1. Tabulation of Group 3 O···H interactions 58

S7.2. Measurements for oxygen deviation of Group 1 59

S7.3. Intermolecular interactions as an aid for atom assignment 59

S8. References 62

S1. Reagents, equipment, data collection, and refinement strategies

Crystalline sponge host crystals ([{$(ZnI_2)_3(tpt)_2 \cdot x(C_6H_{12})$}$_n$] and [{$(ZnI_2)_3(tpt)_2 \cdot x(C_6H_{14})$}$_n$]) were prepared following the reported procedures and supplied by Merck Crystal-Do. (Biradha *et al.*, 2002; Ramadhar *et al.*, 2015)

Single crystal X-ray diffraction experiments were carried out on Rigaku 007HF diffractometer using Cu-K_α radiation (λ = 1.54184 Å). Equipped with Varimax confocal mirrors, UG2 goniometer, and HyPix 6000HE detector. CrysAlis^{Pro} software was used for calculation of experiment strategy and data reduction (data integration, empirical and numerical absorption corrections, and scaling).

All structures were modelled with Olex2 v1.5, solved with SHELXT v2014/5, and refined with SHELXL v2016/6. (Dolomanov *et al.*, 2009; Sheldrick, 2015) Attempts were made to model all non-hydrogen atoms anisotropically. However, low occupancy guests / solvents and overlapping sites prevented stable refinement for some. In these cases, rather than applying severe thermal restraints, the molecule was modelled isotropically with restrained thermal parameters (U_{iso} = 0.08). Hydrogen atoms were fixed with a riding model.

For determination of guest molecule occupancies, non-hydrogen atoms were refined as isotropic with a molecule free-variable (FVAR) and restrained thermal parameters (U_{iso} = 0.08). The guest occupancies were then fixed before anisotropic refinement to prevent expansion of thermal ellipsoids and erroneous assignment of residual electron density within the porous framework. Restraints / constraints were required for most guest molecules and are detailed for each dataset in Section 2. AFIX 66 was applied to all guest aromatic rings for consistency. Solvent molecules were found in the difference map and refined as restrained rigid bodies (DFIX, DANG, SIMU, RIGU, and SADI). The considerable thermal motion of solvent within the pore results in some of the averaged structures possessing energetically unfavourable distortions.

During finalisation of crystallographic structures, some high-intensity reflections were omitted when they possessed an [Error/esd] > 10 as is commonly practiced.

Interaction analysis was conducted when model refinement had been finalised. To identify the interactions, the following programmes were utilised: Olex2, PLATON, and Mercury. (Dolomanov *et al.*, 2009; Spek, 2003; Groom *et al.*, 2016) The guest molecule numbering scheme described in Figure 1 of the main text was used in conjunction with the following framework numbering scheme.

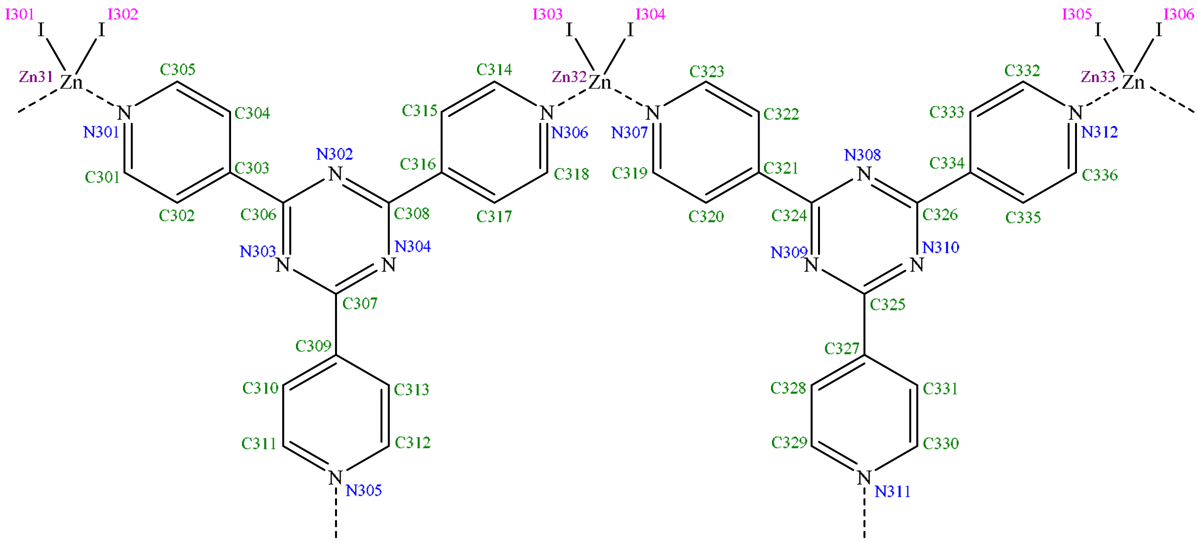

Figure S1 Host framework atom numbering scheme.

S2. Interaction criteria and identification

Reliable analysis and comparison of interactions within confined pores relies on a consistent set of parameters to define contacts. The cut-off criteria for the contacts analysed herein have been derived from accepted standards commonly used in small molecule crystallography, as defined in PLATON. (Spek, 2003) These have also been corroborated with frequency distributions of close contacts found within the Cambridge Structural Database. (Groom *et al.*, 2016) These criteria are summarised below in Table S1.

Table S1 Intermolecular interaction criteria.

Interaction	Distance / Å	Angle / °	Dihedral Angle Between Planes I and J / °	Angle Cg(I) → Cg(J) or Cg(I) → Me vector and normal to plane I / °
X ··· Y	< 3.6	> 60	-	-
X ··· I – Zn*	< 3.6	> 60	-	-
X ··· Cg	< 4.0	> 90	-	-
H ··· Cg	< 3.6	> 90	-	-
Cg ··· Cg	< 6.0	< 20	< 20	< 60

*Interactions only accounted for with the major component of ZnI_2 disorder. Where 'Planes I and J' refer to the planes defined by respective aromatic rings, and Cg(I) / Cg(J) refer to the centre of gravity for rings I and J.

All structures presented in this study possess multiple guest molecules in the asymmetric unit. These guests have the addition of the suffix 'A, B, C, etc.', with the crystallographically better-defined guests labelled first.

To investigate the influence of these structural variations, it is most appropriate to focus on 2 or 3 main interactions. Much like the groupings of the larger compound library, this allows for a more specific analysis as changes in motif and overall effects are incremental and therefore reduce multi-parameter correlations etc., making comparisons more straight forward.

These are deduced by a stepwise process, firstly by considering contact percentages and averages across the series, as shown for Group 1 in Table S2.

Table S2 Group 1 summary of interactions.

% Total Interactions				Average per Guest		
BBA-9-Me	BBA	BBA-3-Me		BBA-9-Me	BBA	BBA-3-Me
14.2	20.8	31.3	Ar ··· Ar	1.33	1.67	2.50
39.3	33.3	18.8	H ··· Ar	3.67	2.67	1.50
21.4	16.7	12.5	O ··· H	2.00	1.33	1.00
10.7	4.2	6.3	O ··· Ar	1.00	0.33	0.50
3.6	8.3	6.3	OH ··· N	0.33	0.67	0.50
7.1	16.7	6.3	CH ··· N	0.67	1.33	0.50
3.6	-	6.3	H ··· I	0.33	-	1.50

Ranking these interactions allows for initial identification of trend 'hotspots', highlighting areas for further analysis of the motifs and patterns which define the intermolecular interactions.

S3. Experimental conditions

Table S3 Guest exchange conditions.

Analyte	Incubation Temp. / º	Incubation Time / hrs	Analyte Solvent	Analyte Conc. / mg mL^{-1}	Analyte Volume / mL	Incubation Temp. / º
BBA-9-Me	50	24	DCM	10	1	50
BBA	50	24	DCM	2	5	50
BBA-3-Me	50	24	-	10	1	50
BBA-10-CN	50	24	DCM	10	1	50
PBA-12-OMe	50	24	DCM	Neat	1	50
BBA-8,12-OMe	50	48	DCM	10	1	50
BBA-8,10,12-OMe	50	24	DCM	10	1	50
PBA	50	24	-	Neat	1	50
PBA-3-F	25	24	DCM	2	5	25
PBA-2-F	50	24	-	Neat	1	50
PBA-2,6-I	50	24	-	Neat	1	50
PBA-2-Me	50	24	DCM	10	1	50
PBA-2-Ph	50	24	DCM	2	5	50

S4. Molecular similarity table

Table S4 Percentage similarity of guest molecules as determined by the Tanimoto coefficient

	**1**	**2**	**3**	**4**	**5**	**6**	**7**	**8**	**9**	**10**	**11**	**12**	**13**
BBA-9-Me (**1**)		81.69	77.26	71.72	35.07	45.90	44.33	40.87	33.74	35.63	33.10	45.15	36.24
BBA (**2**)			92.74	83.63	37.05	49.70	47.49	46.56	35.52	38.41	34.55	42.24	39.82
BBA-3-Me (**3**)				79.49	36.02	48.15	46.10	44.36	34.32	36.96	34.14	40.77	38.25
BBA-10-CN (**4**)					34.60	46.31	44.70	41.96	34.70	36.45	33.10	38.51	37.37
PBA-12-OMe (**5**)						67.44	65.12	67.08	51.09	57.67	51.47	64.09	58.11
BBA-8,12-OMe (**6**)							94.62	59.74	47.81	52.32	47.15	55.96	53.15
BBA-8,10,12-OMe (**7**)								56.52	46.75	50.18	45.65	53.17	52.87
PBA (**8**)									64.85	75.00	65.00	86.67	79.36
PBA-3-F (**9**)										71.77	50.72	60.35	58.06
PBA-2-F (**10**)											55.25	68.07	63.89
PBA-2,6-I (**11**)												61.18	58.18
PBA-2-Me (**12**)													71.17
PBA-2-Ph (**13**)													

S5. Crystallographic tables, notes, and models

Table S5 BBA-9-Me crystallographic data

CCDC Deposition Number	2254202
Empirical formula	$(Zn_3 I_6 N_{12} H_{24} C_{36}) (C_{16} H_{18} O)_{1.01} (C_6 H_{12})_{0.15}$
Formula weight	1824.46
Temperature/K	100(2)
Crystal system	monoclinic
Space group	C2/c
a/Å	35.5224(6)
b/Å	14.9127(2)
c/Å	30.9220(5)
α = γ /°	90
β/°	102.400(2)
Volume/Å^3	15998.3(4)
Z	8
ρ_{calc}g/cm^3	1.515
μ/mm^{-1}	19.515
F(000)	6904.0
Crystal size/mm^3	0.23 × 0.15 × 0.06
Radiation	Cu Kα (λ = 1.54184)
2Θ range for data collection/°	5.852 to 136.496
Index ranges	-41 ≤ h ≤ 42, -17 ≤ k ≤ 8, -36 ≤ l ≤ 37
Reflections collected	52459
Independent reflections	14531 [R_{int} = 0.0187, R_{sigma} = 0.0184]
Data/restraints/parameters	14531/316/1054
Goodness-of-fit on F^2	1.169
Final R indexes [I>=2σ (I)]	R_1 = 0.0479, wR_2 = 0.1419
Largest diff. peak/hole / e Å^{-3}	0.66/-0.78

Exchange site A possessed disorder of methyl and hydroxyl groups in two evenly occupied positions of ~27%. Exchange site B was located over a two-fold symmetry axis and was modelled in PART -1. Exchange site C was found to disordered with a molecule of cyclohexane, the low occupancy of the solvent molecule and overlapping nature led to isotropic refinement of the solvent.

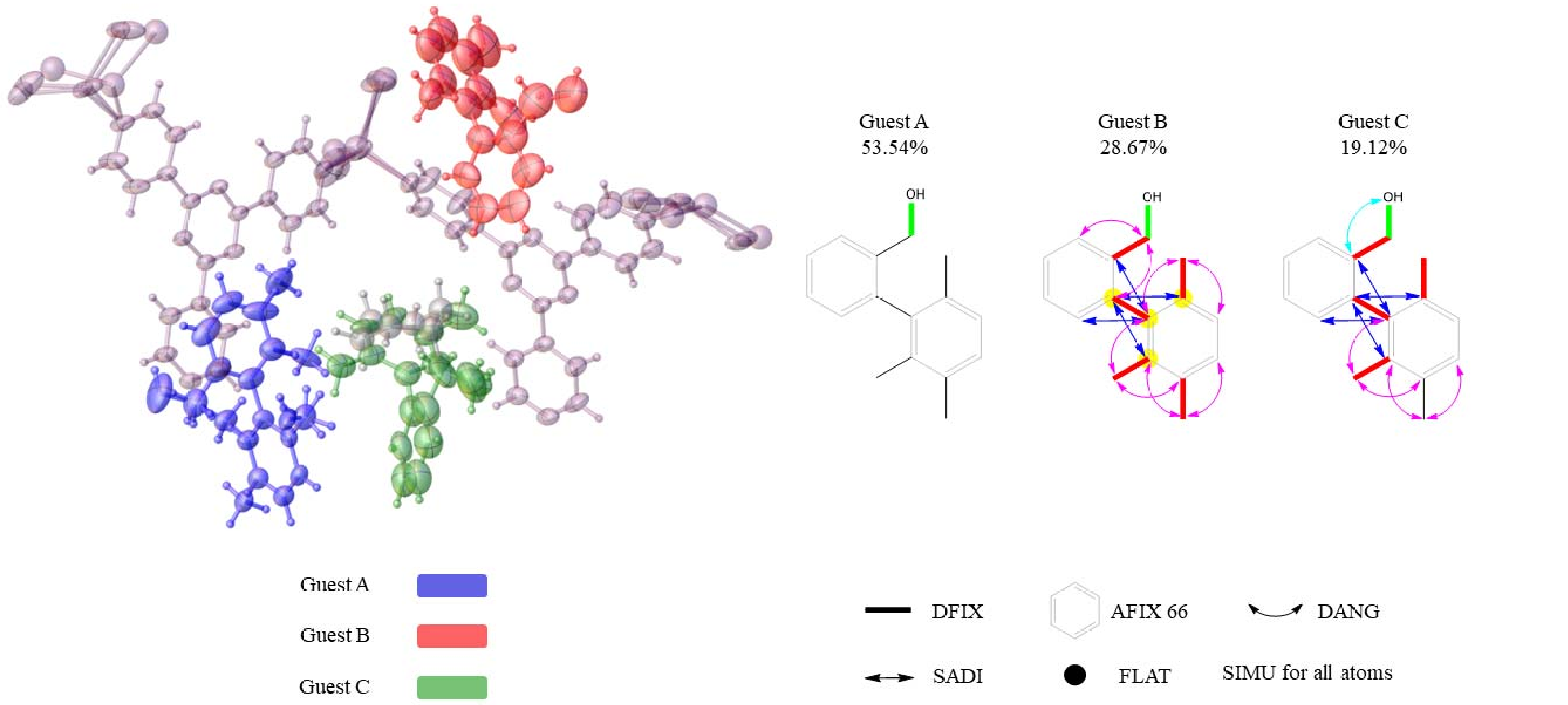

Figure S2 BBA-9-Me asymmetric unit ORTEP diagram shown with 50% probability (left) and restraints / constraints applied in refinement (right).

Table S6 BBA crystallographic table

CCDC Deposition Number	2254203
Empirical formula	$(Zn_3 I_6 N_{12} H_{24} C_{36}) (C_{15} H_{16} O)_{1.33}$
Formula weight	1864.16
Temperature/K	100(2)
Crystal system	monoclinic
Space group	C2/c
a/Å	35.6703(5)
b/Å	14.9029(2)
c/Å	30.5678(6)
α = γ/°	90
β/°	102.1207(15)
Volume/Å^3	15887.3(5)
Z	8
ρ_{calc}g/cm^3	1.559
μ/mm^{-1}	19.669
F(000)	7067.0
Crystal size/mm^3	0.34 × 0.097 × 0.061
Radiation	Cu Kα (λ = 1.54178)
2Θ range for data collection/°	5.068 to 136.486
Index ranges	-42 ≤ h ≤ 33, -17 ≤ k ≤ 17, -36 ≤ l ≤ 36
Reflections collected	89714
Independent reflections	14520 [R_{int} = 0.0361, R_{sigma} = 0.0237]
Data/restraints/parameters	14520/320/947
Goodness-of-fit on F^2	1.209
Final R indexes [I>=2σ (I)]	R_1 = 0.0891, wR_2 = 0.2099
Largest diff. peak/hole / e Å^{-3}	0.85/-0.87

Exchange site C was located over a two-fold symmetry axis and was modelled in PART -1.

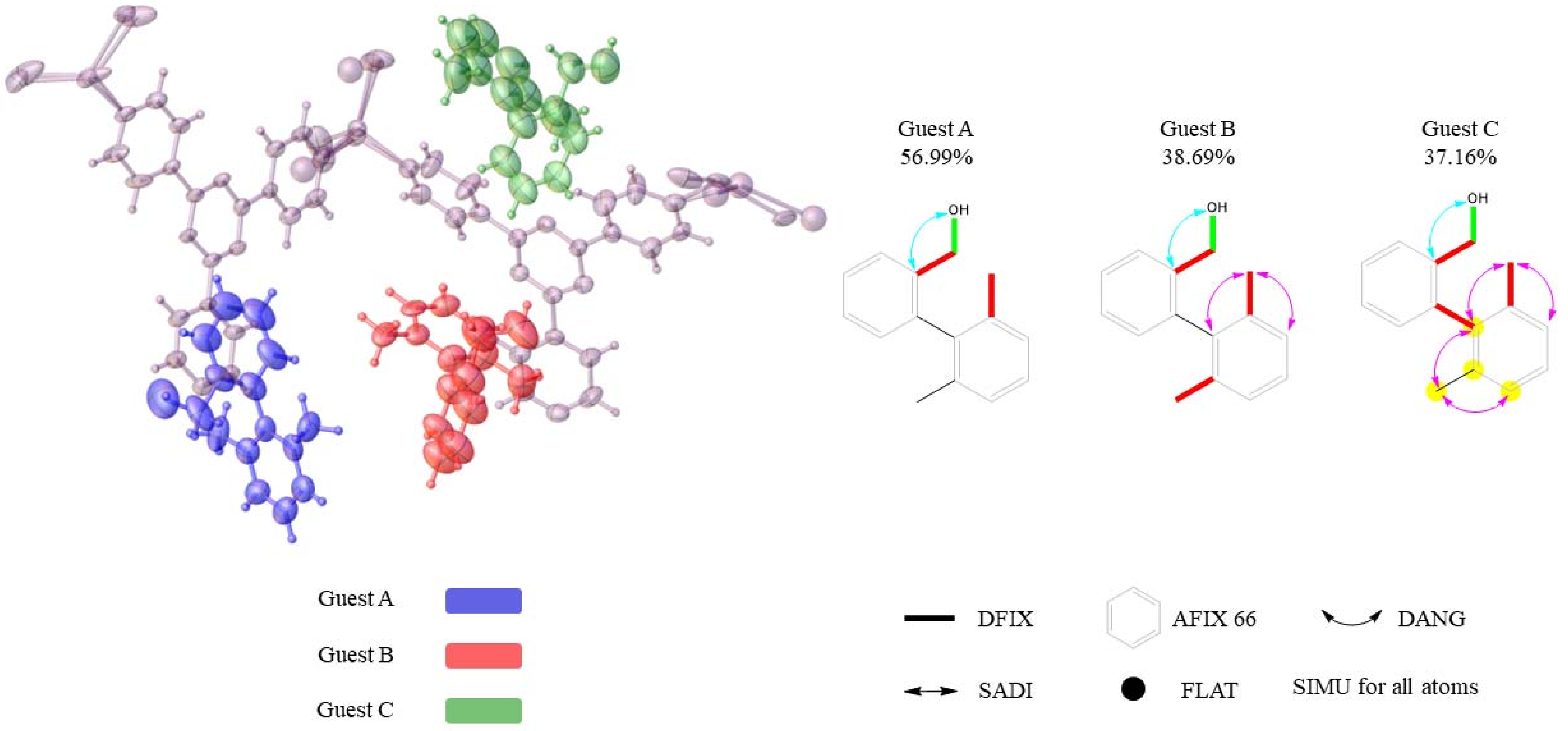

Figure S3 BBA asymmetric unit ORTEP diagram shown with 50% probability (left) and restraints / constraints applied in refinement (right).

Table S7 BBA-3-Me crystallographic table

CCDC Deposition Number	2254204
Empirical formula	$(Zn_3 I_6 N_{12} H_{24} C_{36}) (C_{16} H_{18} O)_{1.33} (C_6 H_{12})_{2.18}$
Formula weight	1826.27
Temperature/K	100(2)
Crystal system	triclinic
Space group	P-1
a/Å	14.8189(3)
b/Å	19.1026(3)
c/Å	31.8140(4)
α/°	101.5570(10)
β/°	92.340(2)
γ/°	110.826(2)
Volume/Å^3	8184.8(3)
Z	4
ρ_{calc}g/cm^3	1.482
μ/mm^{-1}	19.114
F(000)	3464.0
Crystal size/mm^3	0.21 × 0.19 × 0.07
Radiation	Cu Kα (λ = 1.54184)
2Θ range for data collection/°	5.086 to 136.704
Index ranges	-17 ≤ h ≤ 14, -23 ≤ k ≤ 23, -38 ≤ l ≤ 38
Reflections collected	54873
Independent reflections	54873 [R_{int} = 0.142 R_{sigma} = 0.0157]
Data/restraints/parameters	54873/937/1921
Goodness-of-fit on F^2	1.035
Final R indexes [I>=2σ (I)]	R_1 = 0.0644, wR_2 = 0.2018
Largest diff. peak/hole / e Å^{-3}	1.30/-0.91

This structure was twinned (BASF[0.4987(7)]) and the final refinement was completed on the HKLF5 merged file. Exchange site B possessed disorder of the benzyl hydroxyl arm in two positions with occupancies of ~30% and ~20%. Solvent accessible void was present in the final structure, this was analysed further with the SQUEEZE tool available in PLATON. 499 Å^3 contained 102 electrons, this is comparative to 2.13 molecules of cyclohexane or 1.20 molecules of BBA-3-Me.

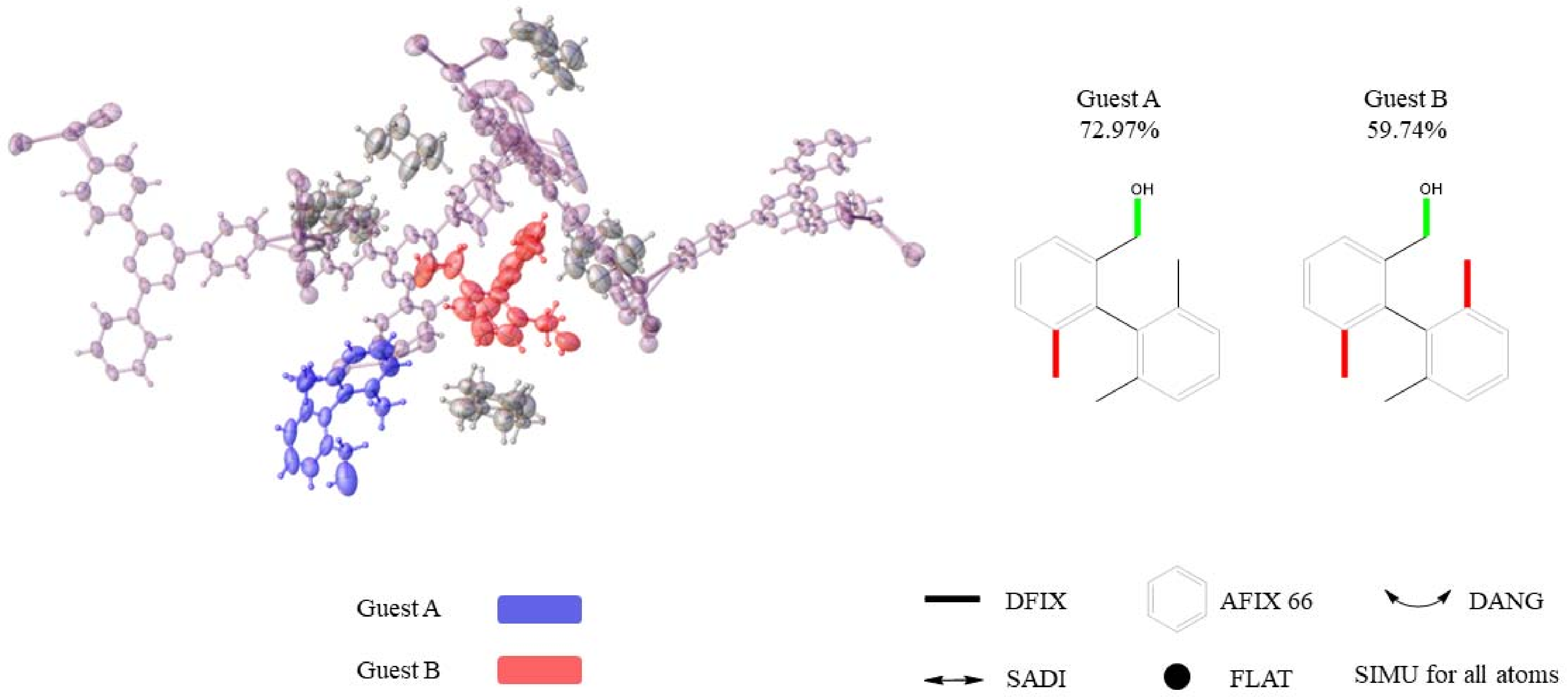

Figure S4 BBA-3-Me asymmetric unit ORTEP diagram shown with 50% probability (left) and restraints / constraints applied in refinement (right).

Table S8 BBA-10-CN crystallographic table

CCDC Number	2254205
Empirical formula	$(Zn_3 I_6 N_{12} H_{24} C_{36}) (C_{16} H_{15} N O)_{0.54} (C_6 H_{12})_{0.21}$
Formula weight	1728.59
Temperature/K	100(2)
Crystal system	monoclinic
Space group	C2/c
a/Å	35.620(2)
b/Å	14.9567(3)
c/Å	30.9945(16)
α = γ/°	90
β/°	102.899(6)
Volume/Å^3	16096.0(14)
Z	8
ρ_{calc}g/cm^3	1.427
μ/mm^{-1}	19.362
F(000)	6483.0
Crystal size/mm^3	0.18 × 0.15 × 0.06
Radiation	Cu Kα (λ = 1.54184)
2Θ range for data collection/°	5.09 to 136.494
Index ranges	-34 ≤ h ≤ 42, -14 ≤ k ≤ 18, -37 ≤ l ≤ 36
Reflections collected	49545
Independent reflections	14578 [R_{int} = 0.0356, R_{sigma} = 0.0275]
Data/restraints/parameters	14578/389/940
Goodness-of-fit on F^2	1.094
Final R indexes [I>=2σ (I)]	R_1 = 0.1280, wR_2 = 0.3302
Largest diff. peak/hole / e Å^{-3}	1.48/-1.33

Exchange site A was located over a two-fold symmetry axis and was modelled in PART -1. Exchange site B was disordered with a solvent molecule but due to the similar occupancies, both could be modelled anisotropically. Solvent accessible void was present in the final structure, this was analysed further with the SQUEEZE tool available in PLATON. 616 Å^3 contained 174 electrons, this is comparative to 3.63 molecules of cyclohexane or 0.72 molecules of BBA-10-CN.

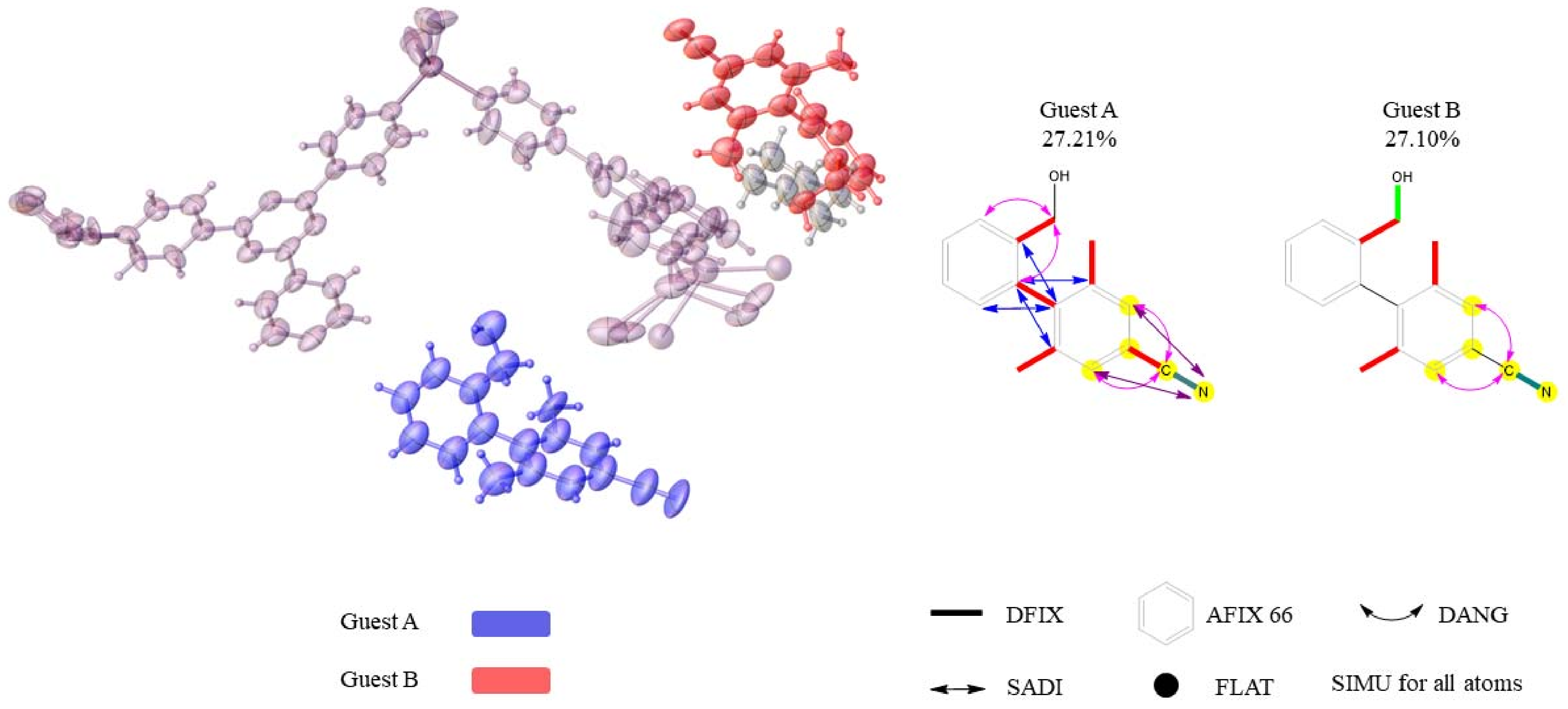

Figure S5 BBA-10-CN asymmetric unit ORTEP diagram shown with 50% probability (left) and restraints / constraints applied in refinement (right).

Table S9 PBA-12-OMe crystallographic table

CCDC Number	2254206
Empirical formula	$(Zn_3 I_6 N_{12} H_{24} C_{36}) (C_{14} H_{14} O_2)_{0.60} (C_6 H_{12})_{0.20}$
Formula weight	1727.08
Temperature/K	100(2)
Crystal system	monoclinic
Space group	C2/c
a/Å	35.3379(7)
b/Å	14.8649(2)
c/Å	31.6259(7)
$\alpha = \gamma$/°	90
β/°	102.979(2)
Volume/Å^3	16188.5(5)
Z	8
ρ_{calc}g/cm^3	1.417
μ/mm^{-1}	19.255
F(000)	6478.0
Crystal size/mm^3	0.21 × 0.15 × 0.07
Radiation	Cu Kα (λ = 1.54184)
2Θ range for data collection/°	5.736 to 136.494
Index ranges	$-42 \le h \le 42$, $-17 \le k \le 9$, $-38 \le l \le 38$
Reflections collected	48192
Independent reflections	14718 [R_{int} = 0.0223, R_{sigma} = 0.0222]
Data/restraints/parameters	14718/314/894
Goodness-of-fit on F^2	1.045
Final R indexes [I>=2σ (I)]	R_1 = 0.0637, wR_2 = 0.2014
Largest diff. peak/hole / e Å^{-3}	1.17/-0.86

Exchange site B was located near a two-fold axis and modelled in PART -1. Both solvent molecules were determined to have low occupancy (10%) and were therefore modelled isotropically. The low occupancy and high thermal motion also resulted in the second solvent molecule being modelled with an energetically unfavourable conformation. Solvent accessible void was present in the final structure, this was analysed further with the SQUEEZE tool available in PLATON. 512 Å^3 contained 128 electrons, this is comparative to 2.66 molecules of cyclohexane or 1.12 molecules of PBA-12-OMe.

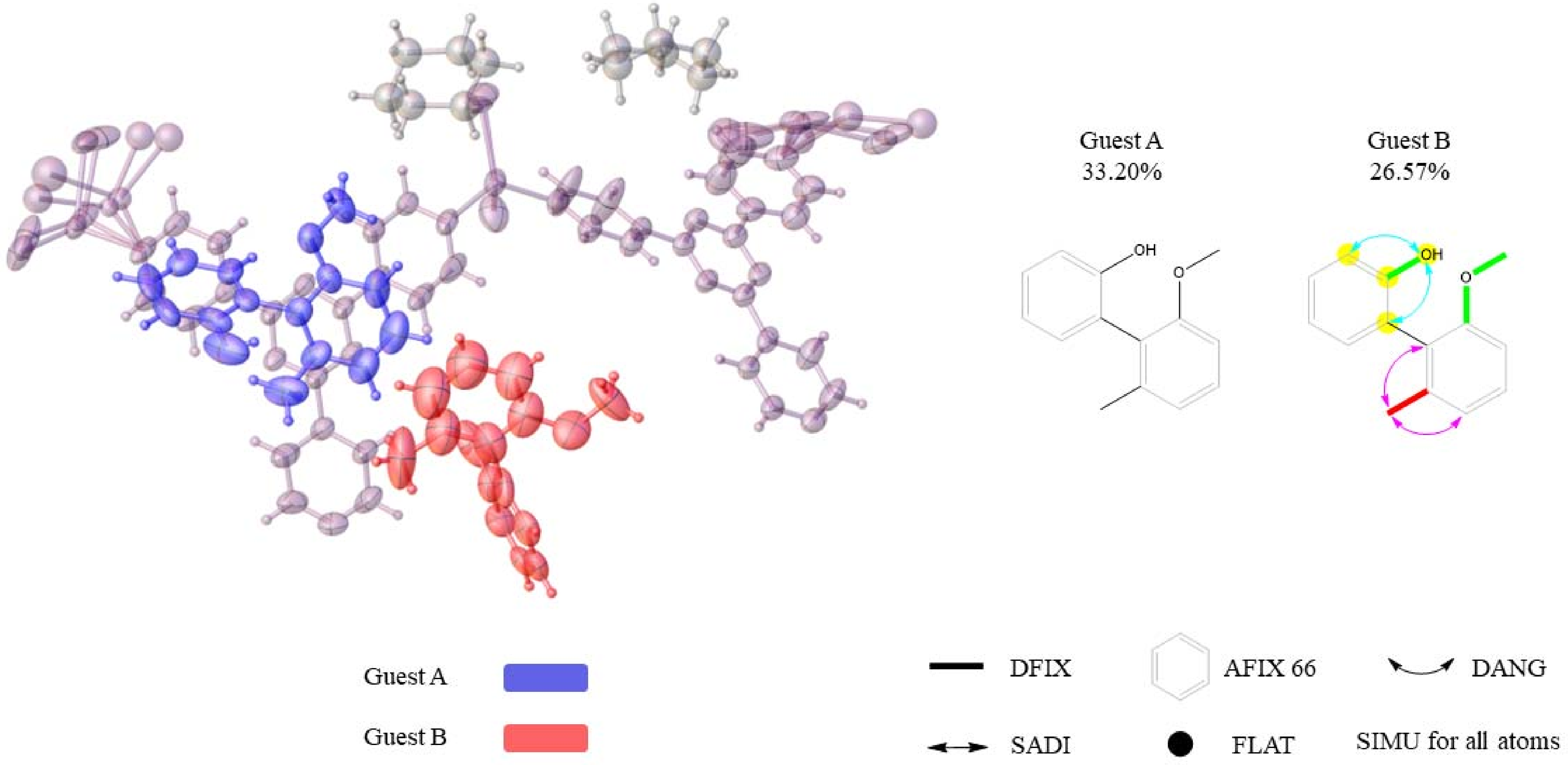

Figure S6 PBA-12-OMe asymmetric unit ORTEP diagram shown with 50% probability (left) and restraints / constraints applied in refinement (right).

Table S10 BBA-8,12-OMe crystallographic table

CCDC Number	2254207
Empirical formula	$(Zn_3 I_6 N_{12} H_{24} C_{36}) (C_{15} H_{16} O_3)_{0.95} (C_6 H_{12})_{2.52}$
Formula weight	1804.11
Temperature/K	100(2)
Crystal system	monoclinic
Space group	P2/n
a/Å	31.5077(5)
b/Å	14.99910(10)
c/Å	34.4289(6)
α = γ/°	90
β/°	102.0860(10)
Volume/Å^3	15910.0(4)
Z	8
ρ_{calc}g/cm^3	1.506
μ/mm^{-1}	19.619
F(000)	6833.0
Crystal size/mm^3	0.28 × 0.17 × 0.07
Radiation	Cu Kα (λ = 1.54184)
2Θ range for data collection/°	4.274 to 136.498
Index ranges	-37 ≤ h ≤ 37, -18 ≤ k ≤ 11, -41 ≤ l ≤ 41
Reflections collected	105990
Independent reflections	29012 [R_{int} = 0.0408, R_{sigma} = 0.0360]
Data/restraints/parameters	29012/1017/1909
Goodness-of-fit on F^2	1.055
Final R indexes [I>=2σ (I)]	R_1 = 0.0708, wR_2 = 0.2085
Largest diff. peak/hole / e Å^{-3}	0.99/-0.91

Exchange site B was located over a two-fold symmetry axis and was modelled in PART -1. Multiple solvent molecules were located over two-fold symmetry sites or were determined to have a low occupancy, these were modelled isotropically to prevent over-expansion of thermal ellipsoids. Solvent accessible void was present in the final structure, this was analysed further with the SQUEEZE tool available in PLATON. 306 Å^3 contained 59 electrons, this is equivalent to 1.23 molecules of cyclohexane or 0.45 molecules of BBA-8,12-OMe.

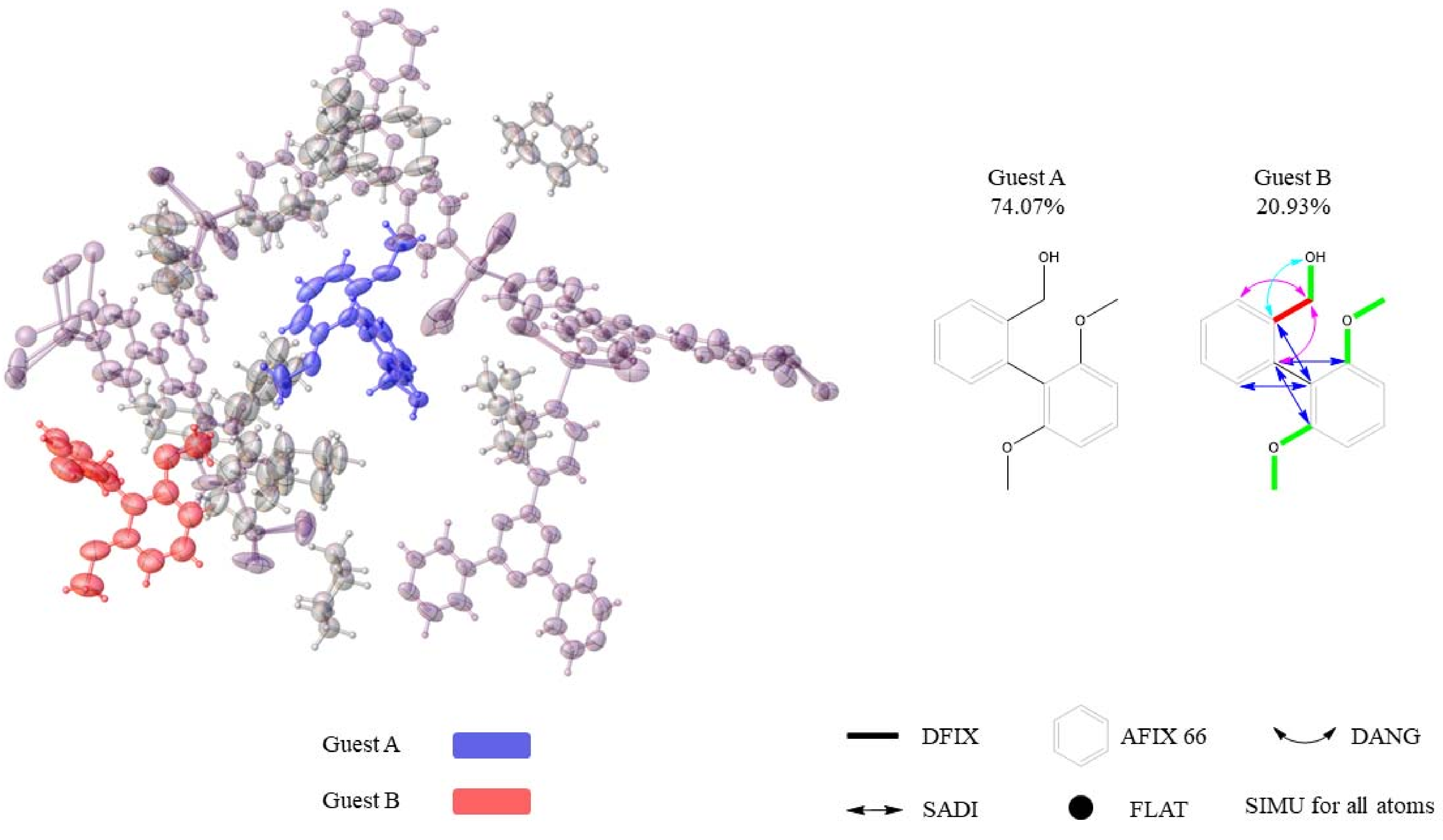

Figure S8 BBA-8,12-OMe asymmetric unit ORTEP diagram shown with 50% probability (left) and restraints / constraints applied in refinement (right).

Table S11 BBA-8,10,12-OMe

CCDC Number	2254208
Empirical formula	$(Zn_3\,I_6\,N_{12}\,H_{24}\,C_{36})\,(C_{16}\,H_{18}\,O_4)_{0.83}\,(C_6\,H_{12})_{0.83}$
Formula weight	1881.81
Temperature/K	100(2)
Crystal system	monoclinic
Space group	C2/c
a/Å	34.533(3)
b/Å	15.0103(9)
c/Å	30.9081(14)
α = γ/°	90
β/°	101.400(6)
Volume/Å^3	15705.3(17)
Z	8
ρ_{calc}g/cm^3	1.592
μ/mm^{-1}	19.921
F(000)	7153.0
Crystal size/mm^3	0.32 × 0.12 × 0.07
Radiation	Cu Kα (λ = 1.54184)
2Θ range for data collection/°	5.222 to 149.97
Index ranges	-42 ≤ h ≤ 43, -18 ≤ k ≤ 18, -38 ≤ l ≤ 33
Reflections collected	46392
Independent reflections	15173 [R_{int} = 0.0541, R_{sigma} = 0.0429]
Data/restraints/parameters	15173/521/1010
Goodness-of-fit on F^2	1.133
Final R indexes [I>=2σ (I)]	R_1 = 0.1172, wR_2 = 0.2682
Largest diff. peak/hole / e Å^{-3}	1.01/-1.52

Exchange site A was located over a two-fold symmetry axis and was modelled in PART -1. The second solvent molecule was disordered with exchange site B and located near a two-fold symmetry axis, this was also modelled in PART -1.

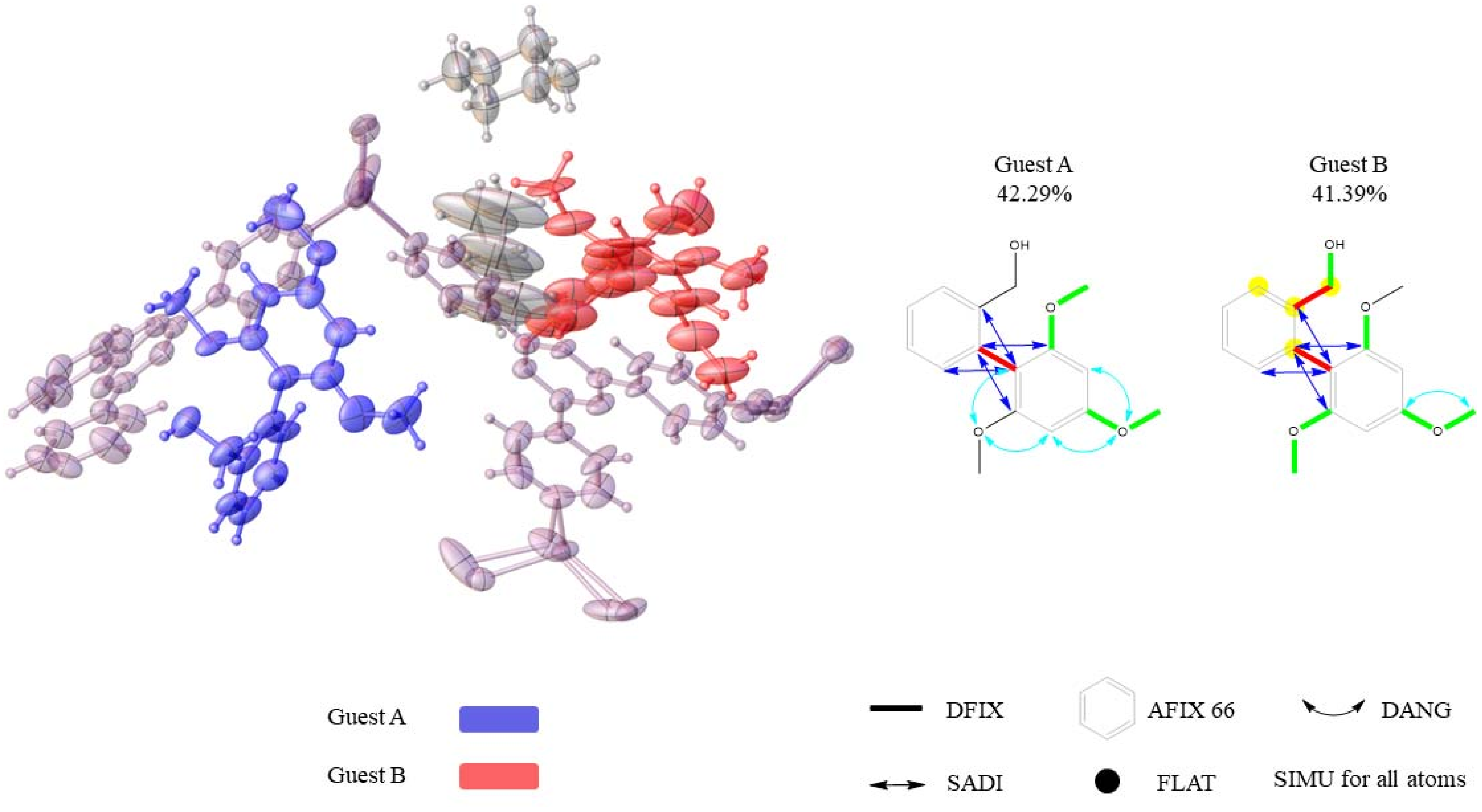

Figure S9 BBA-8,10,12-OMe asymmetric unit ORTEP diagram shown with 50% probability (left) and restraints / constraints applied in refinement (right).

Table S12 PBA crystallographic table

CCDC Number	2254209
Empirical formula	$(Zn_3 I_6 N_{12} H_{24} C_{36}) (C_{13} H_{12} O)_{1.61}$
Formula weight	1881.30
Temperature/K	100(2)
Crystal system	monoclinic
Space group	C2/c
a/Å	35.4079(4)
b/Å	14.9066(2)
c/Å	31.8633(3)
$\alpha = \gamma$/°	90
β/°	103.6640(10)
Volume/Å^3	16341.8(3)
Z	8
ρ_{calc}g/cm^3	1.529
μ/mm^{-1}	19.185
F(000)	7126.0
Crystal size/mm^3	0.21 × 0.16 × 0.07
Radiation	Cu Kα (λ = 1.54184)
2Θ range for data collection/°	5.708 to 136.478
Index ranges	$-42 \le h \le 42$, $-17 \le k \le 15$, $-38 \le l \le 37$
Reflections collected	138492
Independent reflections	14934 [R_{int} = 0.0404, R_{sigma} = 0.0216]
Data/restraints/parameters	14934/605/1256
Goodness-of-fit on F^2	1.063
Final R indexes [I>=2σ (I)]	R_1 = 0.0488, wR_2 = 0.1541
Largest diff. peak/hole / e Å^{-3}	1.84/-1.09

Exchange site E was located over a two-fold symmetry axis and was modelled in PART -1. Solvent accessible void was present in the final structure, this was analysed further with the SQUEEZE tool available in PLATON. 123 Å^3 contained 21 electrons, this is comparative to 0.44 molecules of cyclohexane or 0.21 molecules of PBA.

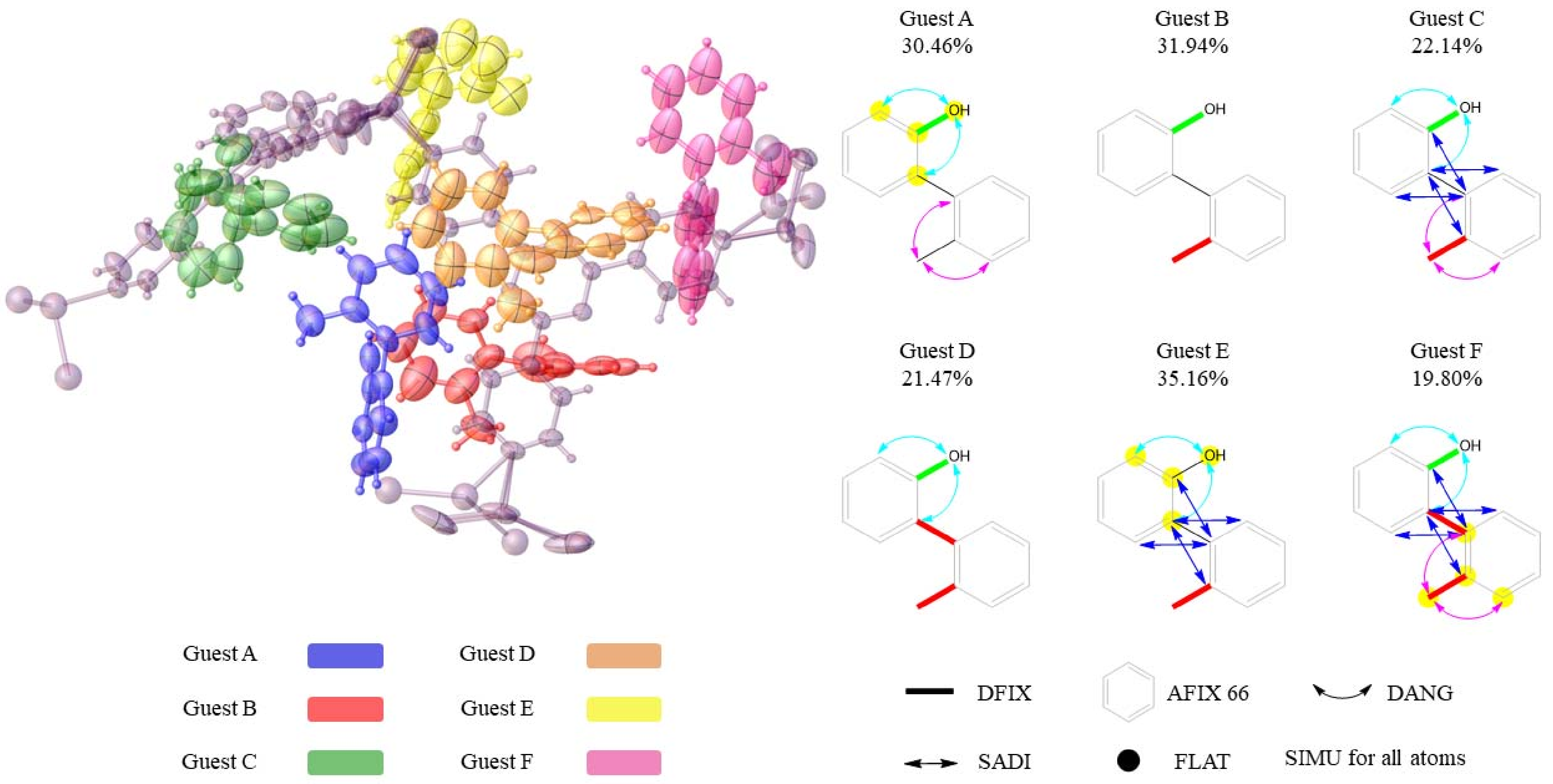

Figure S10PBA asymmetric unit ORTEP diagram shown with 50% probability (left) and restraints / constraints applied in refinement (right).

Table S13 PBA-3-F crystallographic table

CCDC Number	2254210
Empirical formula	$(Zn_3 I_6 N_{12} H_{24} C_{36}) (C_{13} H_{11} O F)_{0.63}$
Formula weight	1710.11
Temperature/K	100(2)
Crystal system	monoclinic
Space group	C2/c
a/Å	35.6889(7)
b/Å	14.8458(2)
c/Å	31.8064(6)
α = γ/°	90
β/°	103.685(2)
Volume/Å^3	16373.6(5)
Z	8
ρ_{calc}g/cm^3	1.387
μ/mm^{-1}	19.038
F(000)	6392.0
Crystal size/mm^3	0.18 × 0.11 × 0.05
Radiation	Cu Kα (λ = 1.54184)
2Θ range for data collection/°	5.096 to 136.494
Index ranges	-42 ≤ h ≤ 42, -17 ≤ k ≤ 9, -38 ≤ l ≤ 38
Reflections collected	54893
Independent reflections	14900 [R_{int} = 0.0269, R_{sigma} = 0.0263]
Data/restraints/parameters	14900/239/891
Goodness-of-fit on F^2	1.031
Final R indexes [I>=2σ (I)]	R_1 = 0.0577, wR_2 = 0.1849
Largest diff. peak/hole / e Å^{-3}	1.83/-1.05

Exchange sites A and C were overlapped, the low occupancy nature of exchange site C led to isotropic modelling to prevent over expansion of thermal ellipsoids for both guest molecules. Fluorine and oxygen atom locations were determined by the procedure described in S7.3. Solvent accessible void was present in the final structure, this was analysed further with the SQUEEZE tool available in PLATON. 775Å^3 contained 183 electrons, this is comparative to 3.81 molecules of cyclohexane or 1.73 molecules of PBA-3-F.

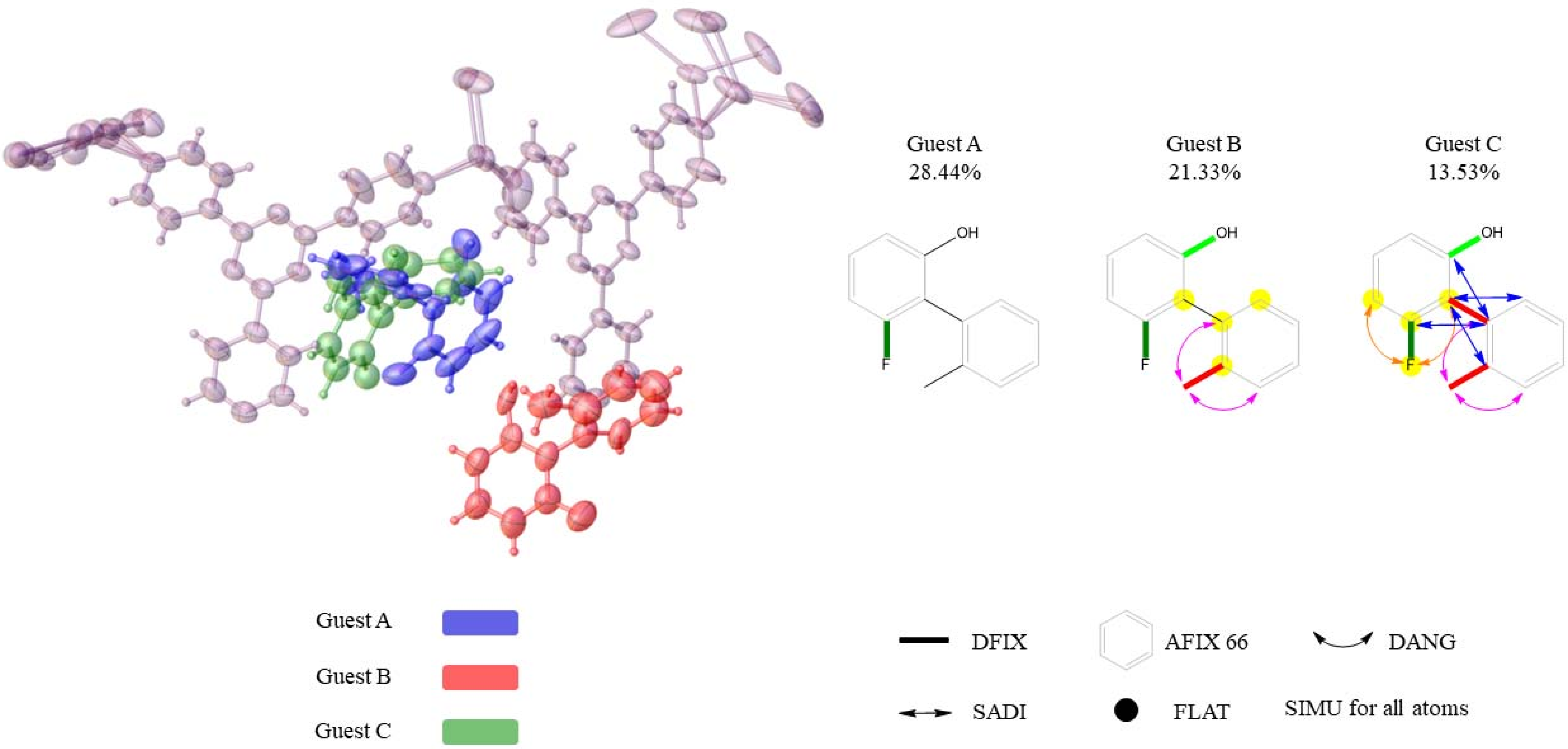

Figure S11 PBA-3-F asymmetric unit ORTEP diagram shown with 50% probability (left) and restraints / constraints applied in refinement (right).

Table S14 PBA-2-F crystallographic table

CCDC Number	2254211
Empirical formula	$(Zn_3 I_6 N_{12} H_{24} C_{36}) (C_{13} H_{11} O F)_{1.06}$
Formula weight	1771.43
Temperature/K	100(2)
Crystal system	monoclinic
Space group	C2/c
a/Å	35.7099(2)
b/Å	14.85945(10)
c/Å	31.59300(19)
α = γ/°	90
β/°	103.0109(6)
Volume/Å^3	16333.77(19)
Z	8
ρ_{calc}g/cm^3	1.441
μ/mm^{-1}	19.114
F(000)	6650.0
Crystal size/mm^3	0.33 × 0.15 × 0.04
Radiation	Cu Kα (λ = 1.54184)
2Θ range for data collection/°	5.742 to 136.5
Index ranges	-42 ≤ h ≤ 42, -17 ≤ k ≤ 16, -38 ≤ l ≤ 38
Reflections collected	307291
Independent reflections	14926 [R_{int} = 0.0451, R_{sigma} = 0.0132]
Data/restraints/parameters	14926/407/1070
Goodness-of-fit on F^2	1.057
Final R indexes [I>=2σ (I)]	R_1 = 0.0482, wR_2 = 0.1544
Largest diff. peak/hole / e Å^{-3}	1.59/-0.85

Solvent accessible void was present in the final structure, this was analysed further with the SQUEEZE tool available in PLATON. 115 Å^3 contained 19 electrons, this is comparative to 0.39 molecules of cyclohexane or 0.18 molecules of PBA-2-F.

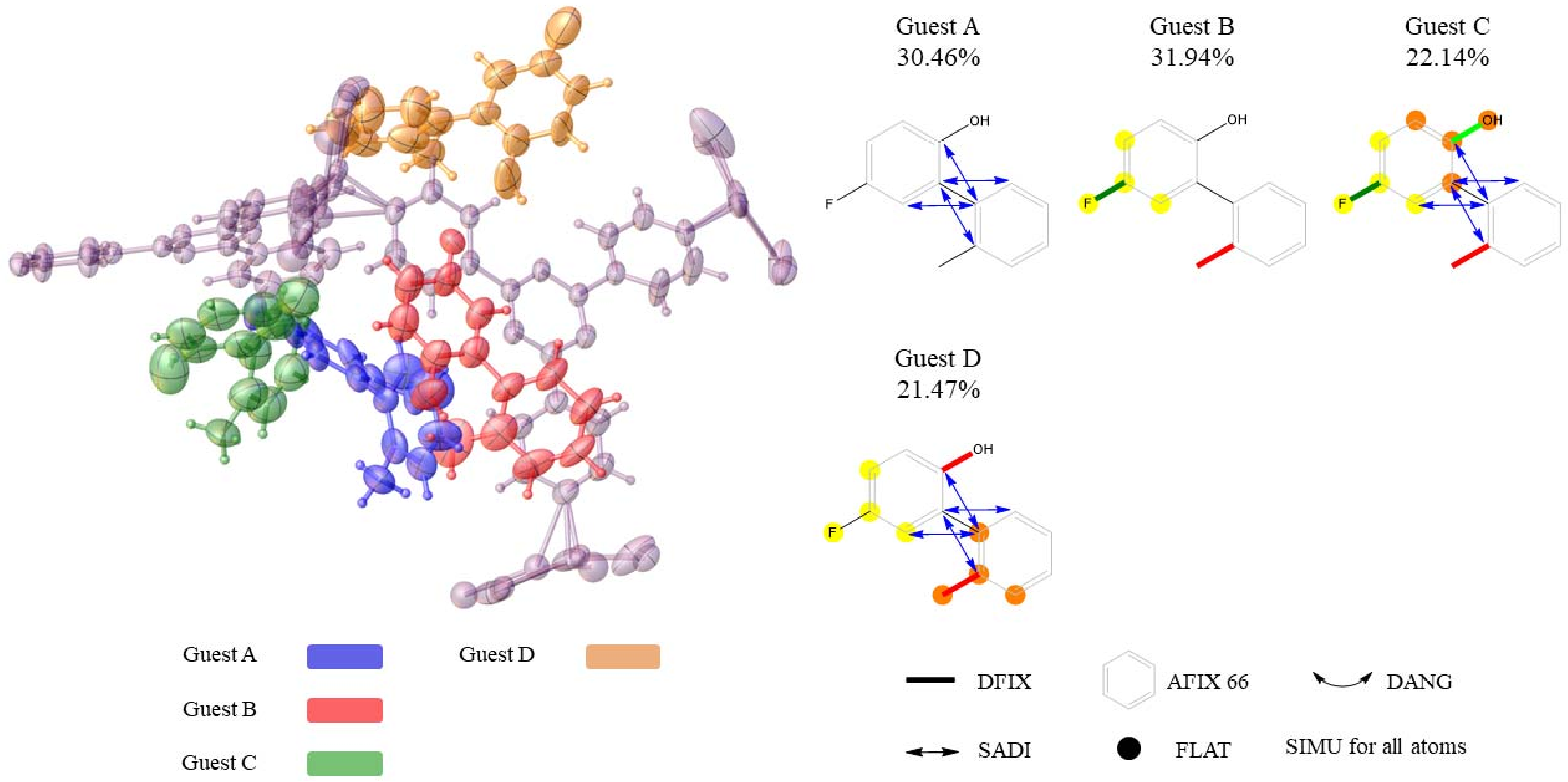

Figure S12PBA-2-F asymmetric unit ORTEP diagram shown with 50% probability (left) and restraints / constraints applied in refinement (right).

Table S15 PBA-2,6-I crystallographic table

CCDC Number	2254212
Empirical formula	$(Zn_3 I_6 N_{12} H_{24} C_{36}) (C_{13} H_{10} O I_2)_{1.50}$
Formula weight	954.52
Temperature/K	100(2)
Crystal system	triclinic
Space group	P-1
a/Å	14.7780(5)
b/Å	18.2345(5)
c/Å	30.7970(5)
α/°	98.227(2)
β/°	91.736(2)
γ/°	113.055(3)
Volume/Å^3	7523.4(4)
Z	8
ρ_{calc}g/cm^3	1.685
μ/mm^{-1}	25.534
F(000)	3536.0
Crystal size/mm^3	0.28 × 0.15 × 0.07
Radiation	Cu Kα (λ = 1.54184)
2Θ range for data collection/°	5.344 to 136.534
Index ranges	-17 ≤ h ≤ 13, -21 ≤ k ≤ 21, -37 ≤ l ≤ 37
Reflections collected	50232
Independent reflections	50232 [R_{int} = 0.112 R_{sigma} = 0.0174]
Data/restraints/parameters	50232/223/1455
Goodness-of-fit on F^2	1.357
Final R indexes [I>=2σ (I)]	R_1 = 0.0922, wR_2 = 0.2967
Largest diff. peak/hole / e Å^{-3}	5.03/-2.65

This structure was twinned (BASF[0.496]) and the final refinement was completed on the HKLF5 merged file. The considerable residual electron density was thought to be caused, in part, by the presence of iodine atoms in the guest molecule and multiple low occupancy exchange sites which could not be modelled. For example, the highest peak of electron density (5.0) within the pore would correspond to an iodine occupancy of 9.43%. The related oxygen and carbon atoms would be difficult to model at this level in even the highest quality CS structures. Solvent accessible void was analysed with the SQUEEZE tool available in PLATON. 992 Å^3 contained 214 electrons, this is comparative to 4.46 molecules of cyclohexane or 0.94 molecules of PBA-2,6-I.

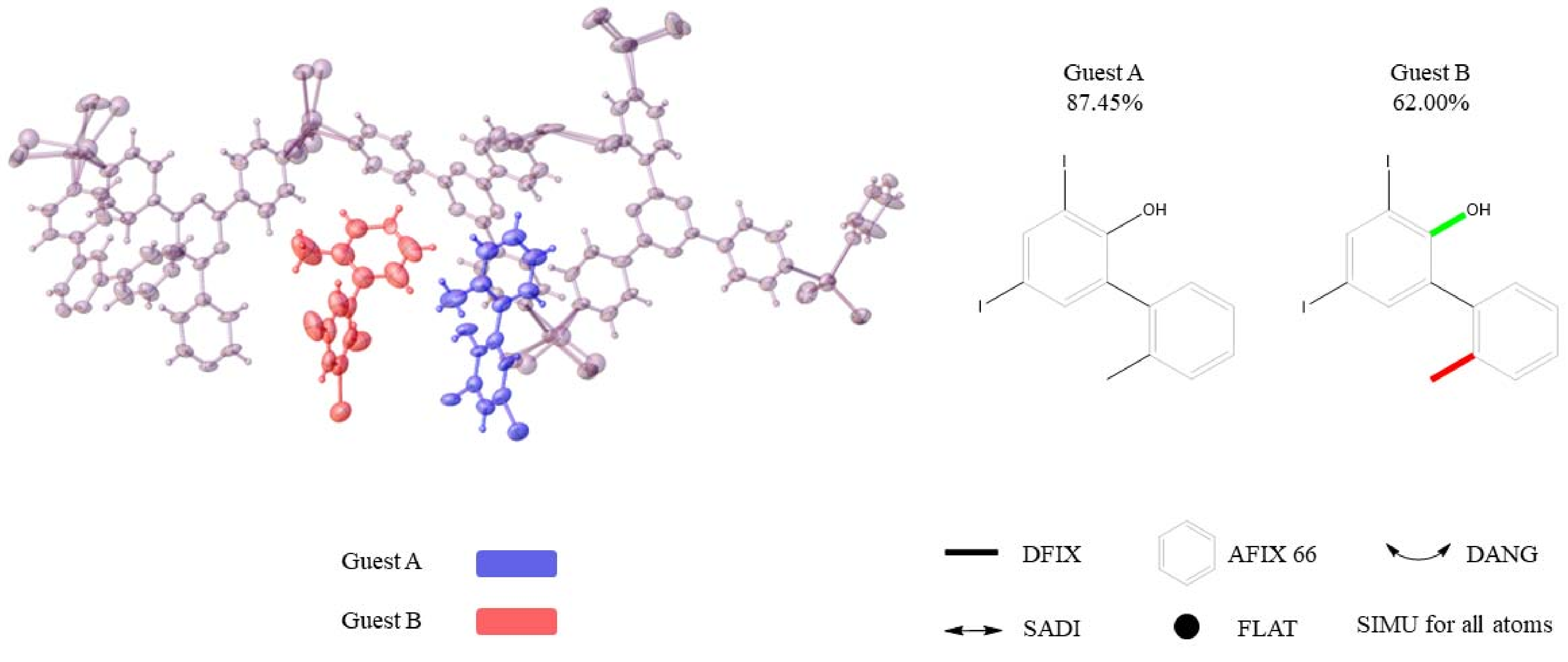

Figure S13PBA-2,6-I asymmetric unit ORTEP diagram shown with 50% probability (left) and restraints / constraints applied in refinement (right).

Table S16 PBA-2-Me crystallographic table

CCDC Number	2254213
Empirical formula	$(Zn_3 I_6 N_{12} H_{24} C_{36}) (C_{14} H_{14} O)_{0.99}$
Formula weight	1780.40
Temperature/K	100(2)
Crystal system	monoclinic
Space group	C2/c
a/Å	35.5509(6)
b/Å	14.9182(3)
c/Å	30.8851(5)
α = γ/°	90
β/°	102.449(2)
Volume/Å^3	15995.0(5)
Z	8
ρ_{calc}g/cm^3	1.479
μ/mm^{-1}	19.505
F(000)	6705.0
Crystal size/mm^3	0.23 × 0.12 × 0.05
Radiation	Cu Kα (λ = 1.54184)
2Θ range for data collection/°	5.092 to 136.496
Index ranges	-37 ≤ h ≤ 42, -17 ≤ k ≤ 14, -37 ≤ l ≤ 37
Reflections collected	46808
Independent reflections	14532 [R_{int} = 0.0311, R_{sigma} = 0.0239]
Data/restraints/parameters	14532/341/994
Goodness-of-fit on F^2	1.051
Final R indexes [I>=2σ (I)]	R_1 = 0.0581, wR_2 = 0.1881
Largest diff. peak/hole / e Å^{-3}	1.10/-0.79

Exchange site A possessed disorder of methyl and hydroxyl groups in two evenly occupied positions of ~25%. Exchange site C was located over a two-fold symmetry axis and was modelled in PART -1.

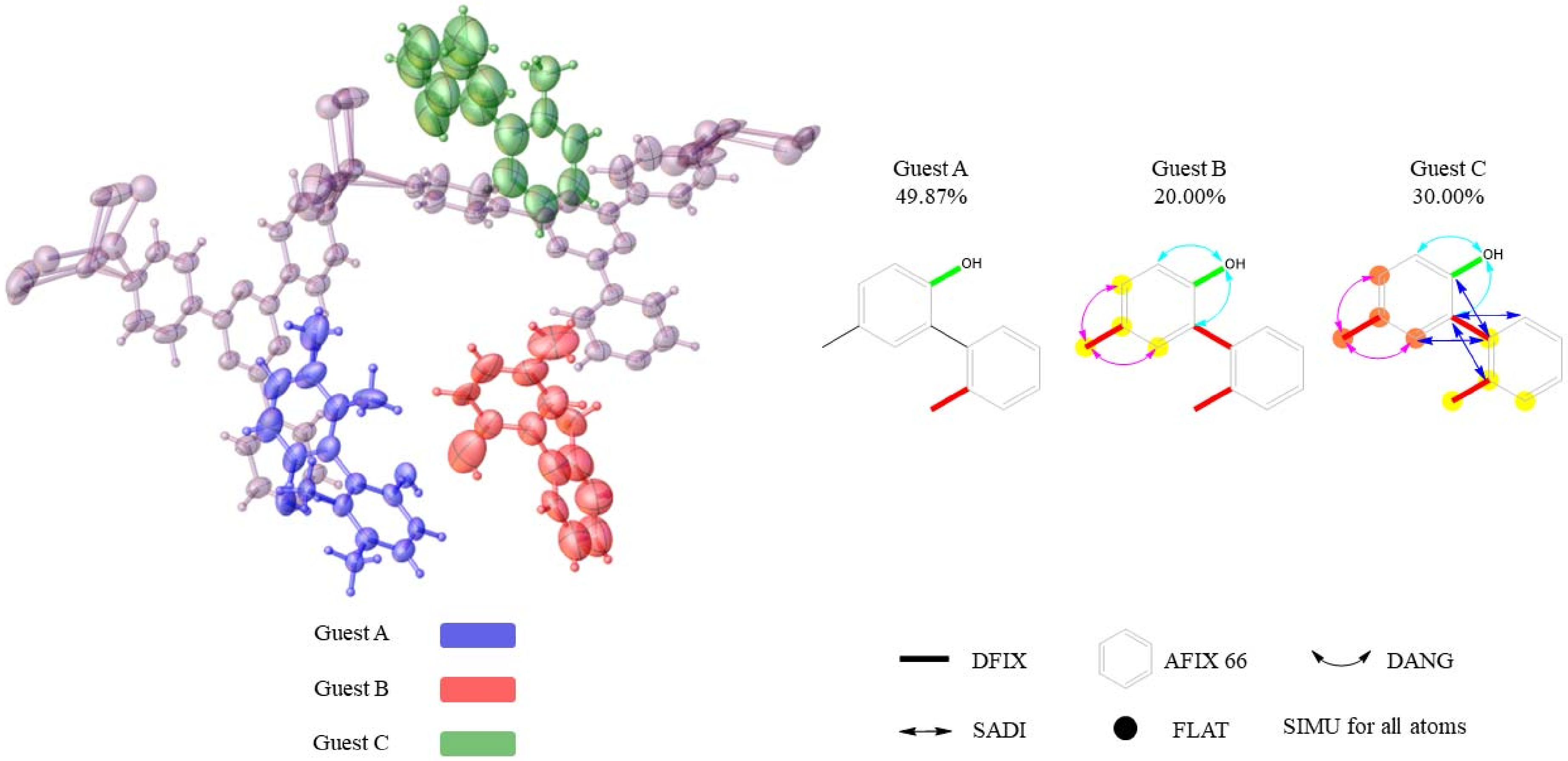

Figure S14PBA-2-Me asymmetric unit ORTEP diagram shown with 50% probability (left) and restraints / constraints applied in refinement (right).

Table S17 PBA-2-Ph crystallographic table

CCDC Number	2254214
Empirical formula	$(Zn_3 I_6 N_{12} H_{24} C_{36}) (C_{19} H_{16} O)_{0.53} (C_6H_{12})_{0.35}$
Formula weight	1750.56
Temperature/K	100(2)
Crystal system	monoclinic
Space group	C2/c
a/Å	34.9529(6)
b/Å	14.9266(2)
c/Å	30.7318(5)
α = γ/°	90
β/°	101.777(2)
Volume/Å^3	15696.1(4)
Z	8
ρ_{calc}g/cm^3	1.482
μ/mm^{-1}	19.861
F(000)	6580.0
Crystal size/mm^3	0.31 × 0.15 × 0.06
Radiation	Cu Kα (λ = 1.54178)
2Θ range for data collection/°	5.166 to 136.49
Index ranges	-42 ≤ h ≤ 40, -14 ≤ k ≤ 17, -37 ≤ l ≤ 35
Reflections collected	49673
Independent reflections	14120 [R_{int} = 0.0280, R_{sigma} = 0.0266]
Data/restraints/parameters	14120/368/962
Goodness-of-fit on F^2	1.037
Final R indexes [I>=2σ (I)]	R_1 = 0.0587, wR_2 = 0.1757
Largest diff. peak/hole / e Å^{-3}	1.03/-0.86

Both exchange sites A and B were located over two-fold symmetry axes and were modelled in PART -1 and -2 respectively. The high thermal motion of the solvent molecule resulted in it being modelled with an energetically unfavourable conformation.

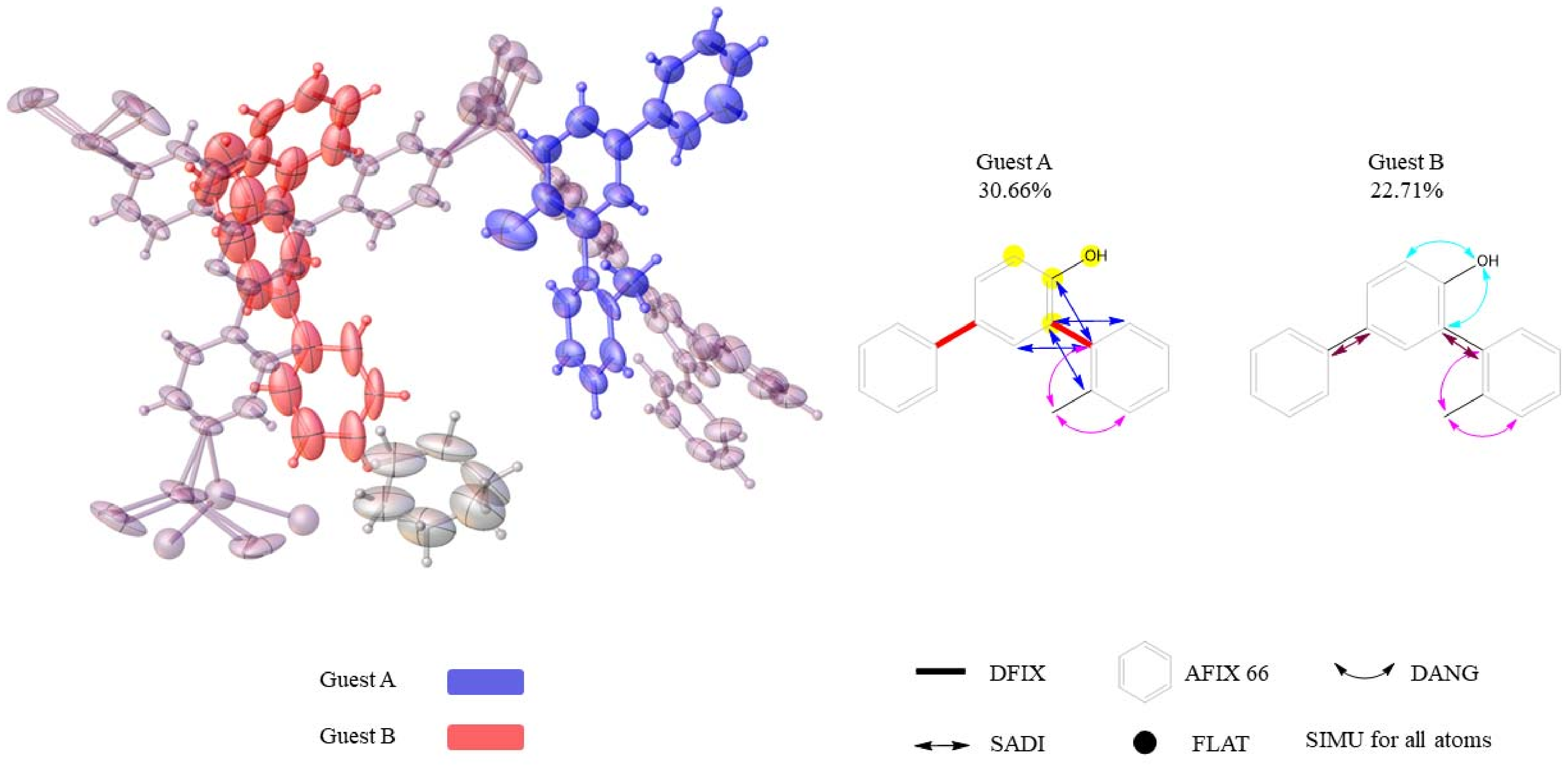

Figure S15 PBA-2-Ph asymmetric unit ORTEP diagram shown with 50% probability (left) and restraints / constraints applied in refinement (right).

S6. Full lists of intermolecular interactions

Table S18 BBA-9-Me atom interactions

Type 1	Type 2	Component 1	Component 2	Symmetry Operator 1	Symmetry Operator 2	Length / Å	Angle / °
Host - Guest	H ··· Ar	H13B	C319 - C323 Cen.	x,y,z	1/2-x,3/2-y,1-z	3.068(5)	136.8(11)
		H15B	C319 - C323 Cen.	x,y,z	1/2-x,3/2-y,1-z	3.510(5)	169.5(12)
		C1B - C6B Cen.	H304	x,y,z	x,y,z	3.162(7)	138.0(6)
		C1B - C6B Cen.	H305	x,y,z	x,y,z	3.766(7)	113.2(5)
		C1B - C6B Cen.	H304	x,y,z	1-x,+y,1/2-z	3.239(7)	126.5(5)
		C1B - C6B Cen.	H305	x,y,z	1-x,+y,1/2-z	3.392(7)	121.0(5)
		H2C	C327 - C331 Cen.	x,y,z	1/2-x,1/2+y,1/2-z	3.245(6)	133.8(6)
	O ··· H	O2A	H329	x,y,z	x,y,z	2.38(3)	138.8(15)
		O1B	H330	x,y,z	1/2+x,-1/2+y,+z	2.80(2)	138(3)
		O1B	H331	x,y,z	1/2+x,-1/2+y,+z	3.12(3)	144(3)
	O ··· Ar	O1A	C301 - C305 Cen.	x,y,z	-1/2+x,1/2+y,+z	3.44(3)	110.3(19)
		O1A	C327 - C331 Cen.	x,y,z	x,y,z	3.88(2)	94.3(14)
		O2A	C332 - C335 Cen.	x,y,z	1/2-x1/2+y,1/2-z	3.44(2)	92.3(11)
	C-H ··· N	H1CA	N305	x,y,z	1-x,+y,1/2-z	3.201(5)	148(4)
		H3C	N310	x,y,z	1/2-x,-1/2+y,1/2-z	2.958(5)	148.8(9)
		H16I	N309	x,y,z	1/2-x,-1/2+y,1/2-z	3.032(5)	134.8(17)
	H ··· I	H18A	I305	x,y,z	1/2-x,-1/2+y,1/2-z	3.584(3)	101.1(18)
Guest - Guest	H ··· Ar	H6A	C1C - C6C Cen.	x,y,z	1/2-x,-1/2+y,1/2-z	3.109(5)	149.1(8)
		H18A	C1A - C6A Cen.	x,y,z	1/2-x,3/2-y,1-z	2.73(6)	132(3)
		H13D	C1C - C6C Cen.	x,y,z	1-x,-1+y,1/2-z	3.277(4)	113(2)
		H15D	C1C - C6C Cen.	x,y,z	1-x,-1+y,1/2-z	3.104(5)	173.7(19)
	O ··· H	H17A	O1C	x,y,z	+x,2-y,1/2+z	3.80(6)	60(3)
		H15D	O1C	x,y,z	1-x,-1+y,1/2-z	2.60(6)	88(3)
		H15F	O1C	x,y,z	1-x,-1+y,1/2-z	3.03(6)	119(4)

Table S19 BBA-9-Me ring interactions

Type 1	Type 2	Component 1	Component 2	Symmetry Operator 1	Symmetry Operator 2	Cg – Cg / Å	α / °	Slippage / Å
Host - Guest	Ar ··· Ar	C332 - C335 Cen.	C7C - C12C Cen.	x,y,z	1/2-x,1/2+y,1/2-z	4.519(11)	16.5(8)	2.166
		C324 - C326 Cen.	C1A - C6A Cen.	x,y,z	x,y,z	5.795(4)	9.9(4)	4.814
		C332 - C335 Cen.	C1A - C6A Cen.	x,y,z	x,y,z	5.064(5)	2.3(4)	3.940
Guest - Guest	Ar ··· Ar	C1A - C6A Cen.	C1A - C6A Cen.	x,y,z	1/2-x,3/2-y,1-z	4.678(5)	0.0(5)	2.908

N.B. The following atoms / rings are shared by two overlapping molecules: C1A – C6A Cen., C7A – C12A Cen., H1A, H6A, H10A, and H11A. Any interactions involving these atoms or rings have been counted twice in determination of % contribution and averages.

Table S20 BBA atom interactions

Type 1	Type 2	Component 1	Component 2	Symmetry Operator 1	Symmetry Operator 2	Length / Å	Angle / °
Host - Guest	H ··· Ar	H15B	C333 - C336 Cen.	x,y,z	1/2-x,1/2+y,1/2-z	3.447(5)	113.1(13)
		H2B	C327 - C331 Cen.	x,y,z	1/2-x,1/2+y,1/2-z	3.451(5)	144.8(15)
		H15D	C324 - C326 Cen.	x,y,z	1/2-x,1/2+y,1/2-z	3.201(5)	132(2)
		C1C - C6C Cen.	H304	x,y,z	x,y,z	2.955(5)	130.9(9)
		C1C - C6C Cen.	H305	x,y,z	x,y,z	3.194(5)	121.3(8)
		C7C - C12C Cen.	H314	x,y,z	x,y,z	3.589(4)	116.7(11)
	O ··· H	O1B	H312	x,y,z	+x,2-y,-1/2+z	2.54(2)	157(4)
		O1C	H330	x,y,z	1/2+x,-1/2+y,+z	2.71(4)	127.3(12)
	O ··· Ar	O1A	C301 - C305 Cen.	x,y,z	-1/2+x,1/2+y,+z	3.68(3)	106.5(18)
	O-H ··· N	H1AA	N301	x,y,z	1/2+x,-1/2+y,z	3.074(10)	120(2)
		H1AA	N311	x,y,z	x,y,z	2.834(11)	161.4(17)
	C-H ··· N	H13A	N307	x,y,z	1/2-x,3/2-y,1-z	3.368(10)	116(2)
		H15B	N312	x,y,z	1/2-x,1/2+y,1/2-z	3.502(10)	121.9(15)
		H3B	N310	x,y,z	1/2-x,-1/2+y,1/2-z	2.81(5)	146.1(18)
		H13I	N302	x,y,z	x,y,z	2.61(6)	152(3)
Guest - Guest	H ··· Ar	H2A	C1A - C6A Cen.	x,y,z	1/2-x,3/2-y,1-z	3.645(5)	97.6(9)
		H13F	C7C - C12C Cen.	x,y,z	1-x,1+y,1/2-z	3.58(4)	115(2)
	O ··· H	O1A	H1B	x,y,z	1/2-x,-1/2+y,1/2-z	2.87(7)	128(2)
		O1A	H2B	x,y,z	1/2-x,-1/2+y,1/2-z	2.66(7)	141(2)

Table S21 BBA ring interactions

Type 1	Type 2	Component 1	Component 2	Symmetry Operator 1	Symmetry Operator 2	Cg – Cg / Å	α / °	Slippage / Å
Host - Guest	Ar ··· Ar	C324 - C326 Cen.	C1A - C6A Cen.	x,y,z	x,y,z	5.936(14)	10.0	4.952
		C327 - C331 Cen.	C1A - C6A Cen.	x,y,z	x,y,z	5.168(15)	2.0	4.068
		C332 - C336 Cen.	C7B - C12B Cen.	x,y,z	1/2-x,1/2+y,1/2-z	4.048(12)	12.0	1.871
Guest - Guest	Ar ··· Ar	C1A - C6A Cen.	C1A - C6A Cen.	x,y,z	1/2-x,3/2-y,1-z	4.514(19)	0	2.870
		C7A - C12A Cen.	C7A - C12A Cen.	x,y,z	1/2-x,5/2-y,1-z	4.945(15)	0.0	3.612

Table S22 BBA-3-Me atom interactions

Type 1	Type 2	Component 1	Component 2	Symmetry Operator 1	Symmetry Operator 2	Length / Å	Angle / °
Host - Guest	H ··· Ar	H14E	C324 - C326 Cen.	x,y,z	x,y,z	2.963(7)	130.0(16)
	O ··· H	O2B	H312	x,y,z	1-x,1-y,-z	2.95(5)	134.4(12)
	O ··· Ar	O1A	C319 - C323 Cen.	x,y,z	1-x,1-y,-z	3.094(17)	132.9(12)
	C-H ··· N	H1BA	N308	x,y,z	x,y,z	3.170(7)	82(3)
		H14C	N308	x,y,z	x,y,z	2.607(8)	128.5(15)
	H ··· I	H9B	I306	x,y,z	x,y,z	3.128(4)	138.98(8)
		H13D	I304	x,y,z	1-x,1-y,-z	3.170(15)	141.6(8)
		H14F	I304	x,y,z	1-x,1-y,-z	3.270(16)	130.5(8)
Guest - Guest	H ··· Ar	H1B	C1A - C6A Cen.	x,y,z	1-x,1-y,-z	3.707(6)	119.1(9)
		H6B	C1A - C6A Cen.	x,y,z	1-x,1-y,-z	3.293(5)	137.3(9)
	O ··· H	O1B	H1AA	x,y,z	1-x,1-y,-z	2.21(4)	139(5)

Table S23 BBA-3-Me ring interactions

Type 1	Type 2	Component 1	Component 2	Symmetry Operator 1	Symmetry Operator 2	Cg – Cg / Å	α / °	Slippage / Å
Host - Guest	Ar ··· Ar	C319 - C323 Cen.	C7A - C12A Cen.	x,y,z	x,y,z	5.991(6)	13.1(5)	5.184
		C324 - C326 Cen.	C7A - C12A Cen.	x,y,z	x,y,z	5.772(5)	14.4(4)	4.659
		C327 - C331 Cen.	C7A - C12A Cen.	x,y,z	x,y,z	4.963(6)	0.2(5)	3.812
		C332 – C336 Cen.	C7B – C12B Cen.	x,y,z	x,y,z	3.973(7)	23.2(4)	2.291(13)
Guest - Guest	Ar ··· Ar	C1A - C6A Cen.	C1A - C6A Cen.	x,y,z	1-x,1-y,-z	5.097(5)	0.0(4)	3.741
		C7A - C12A Cen.	C7A - C12A Cen.	x,y,z	1-x,1-y,-z	4.929(6)	0.0(5)	3.454(4)

N.B. The following atoms / rings are shared by two overlapping molecules: C1B – C6B Cen., C7B – C12B Cen., H1A, H2A, H6A, H9B, H10B, and H11B. Any interactions involving these atoms or rings have been counted twice in determination of % contribution and averages.

Table S24 BBA-10-CN atom interactions

Type 1	Type 2	Component 1	Component 2	Symmetry Operator 1	Symmetry Operator 2	Length / Å	Angle / °
Host - Guest	H ··· Ar	H1AA	C301 - C305 Cen.	x,y,z	1-x,+y,3/2-z	3.121(19)	146(4)
		C1B - C6B Cen.	H310	x,y,z	1/2-x,3/2-y,1-z	3.639(2)	139.6(14)
	O ··· H	O1B	H329	x,y,z	1/2+x,1/2+y,z	2.63(4)	160.2(17)
	X ··· Ar	N1B	C319 - C323 Cen.	x,y,z	-1/2+x,-1/2+y,+z	3.77(6)	109(4)
	O-H ··· N	H1AA	N301	x,y,z	1-x,+y,3/2-z	3.356(16)	129(4)
	C-H ··· N	N1A	H331	x,y,z	1/2+x,-1/2+y,z	2.59(6)	172(3)
		N1A	H335	x,y,z	1/2+x,-1/2+y,z	2.55(6)	111(3)
		H15B	N301	x,y,z	x,y,z	3.532(15)	107(3)
		N1B	H320	x,y,z	1/2+x,1/2+y,z	3.10(5)	86.1(17)
	H ··· I	H1BA	I301	x,y,z	x,y,z	3.016(4)	131(3)
Guest - Guest	C-H ··· N	H13C	N1B	x,y,z	1/2-x,1/2-y,1-z	3.02(5)	111(3)
		H2A	N1B	x,y,z	1-x,-1+y,3/2-z	2.25(6)	163.3(12)
		H3B	N1B	x,y,z	1/2-x,1/2-y,1-z	2.86(6)	123.7(12)

Table S25 BBA-10-CN ring interactions

Type 1	Type 2	Component 1	Component 2	Symmetry Operator 1	Symmetry Operator 2	Cg – Cg / Å	α / °	Slippage / Å
Host - Guest	Ar ··· Ar	C309 - C312 Cen.	C1B - C6B Cen.	x,y,z	1/2-x,3/2-y,1-z	5.60(2)	18.0	4.355
		C327 - C331 Cen.	C7B - C12B Cen.	x,y,z	-1/2+x,-1/2+y,z	5.272(17)	3.0	4.221
Guest - Guest	Ar ··· Ar	C7B - C12B Cen.	C7B - C12B Cen.	x,y,z	1/2-x,1/2-y,1-z	4.45(2)	0.0	2.938
		C1B - C6B Cen.	C1B - C6B Cen.	x,y,z	1/2-x,3/2-y,1-z	4.91(2)	0.0	3.671

Table S26 PBA-12-OMe atom interactions

Type 1	Type 2	Component 1	Component 2	Symmetry Operator 1	Symmetry Operator 2	Length / Å	Angle / °
Host-Guest	H ⋯ Ar	H1BA	C301 - C305 Cen.	x,y,z	1/2-x,-1/2+y,1/2-z	3.023(5)	131.4(14)
		H6B	C301 - C305 Cen.	x,y,z	1/2-x,-1/2+y,1/2-z	3.059(6)	130.25(16)
		C1B - C6B Cen.	H330	x,y,z	1-x,-y,1-z	2.916(5)	120.5(6)
		C1B - C6B Cen.	H331	x,y,z	1-x,-y,1-z	3.298(8)	105.9(5)
	O ⋯ H	O1A	H331	x,y,z	-1/2+x,1/2+y,+z	2.75(3)	128.0(12)
		O2A	H335	x,y,z	1/2+x,-1/2+y,+z	3.056(15)	124.5(8)
		O2A	H336	x,y,z	1/2+x,-1/2+y,+z	2.540(18)	142.9(9)
		O1B	H305	x,y,z	1/2-x,-1/2+y,1/2-z	2.76(2)	139.0(13)
		O1B	H312	x,y,z	x,y,z	3.358(19)	103.1(9)
		O1B	H313	x,y,z	x,y,z	2.55(2)	125.4(11)
		O2B	H330	x,y,z	1-z,-y,1-z	2.57(2)	117.4(13)
	OH ⋯ N	H1BA	N301	x,y,z	1/2-x,-1/2+y,1/2-z	3.049(8)	105.8(14)
	CH ⋯ N	H13A	N303	x,y,z	x,y,z	2.94(4)	137(2)
		H13A	N304	x,y,z	x,y,z	2.63(4)	137.7(18)
	H ⋯ I	H14D	I302	x,y,z	1/2-x,-1/2+y,1/2-z	2.942(16)	133.8(19)
Guest - Guest	H ⋯ Ar	H3B	C7A - C12A Cen.	x,y,z	1/2-x,1/2-y,1-z	2.790(15)	146.05(9)
		H9B	C7B - C12B Cen.	x,y,z	1/2-x,1/2-y,1-z	2.288(4)	103.48(13)
	O ⋯ H	O1A	H3B	x,y,z	1/2-x,1/2-y,1-z	2.84(3)	108.9(11)
		O1A	H14E	x,y,z	1/2-x,1/2-y,1-z	2.548(18)	114.9(19)
		O2A	H2B	x,y,z	1/2-x,1/2-y,1-z	3.362(16)	100.9(8)
		O2B	H1AA	x,y,z	1/2-x,1/2-y,1-z	2.47(4)	118.9(17)
		O2B	H13C	x,y,z	1/2-x,1/2-y,1-z	3.51(3)	75.7(10)

Table S27 PBA-12-OMe ring interactions

Type 1	Type 2	Component 1	Component 2	Symmetry Operator 1	Symmetry Operator 2	Cg – Cg / Å	α / °	Slippage / Å
Host - Guest	Ar ⋯ Ar	C306 - C308 Cen.	C7A - C12A Cen.	x,y,z	x,y,z	4.366(7)	17.7(5)	2.740
		C333 - C336 Cen.	C1A - C6A Cen.	x,y,z	1/2+x,-1/2+y,+z	3.864(6)	14.6(5)	1.340
Guest - Guest	Ar ⋯ Ar	C7B - C12B Cen.	C7A - C12A Cen.	x,y,z	1/2-x,1/2-y,1-z	4.337(13)	18	1.682

Table S28 BBA-8,12-OMe atom interactions

Type 1	Type 2	Component 1	Component 2	Symmetry Operator 1	Symmetry Operator 2	Length / Å	Angle / °
Host - Guest	O ⋯ H	O1A	H340	x,y,z	1/2-x,+y,1/2-z	2.740(16)	95.6(9)
		O1A	H341	x,y,z	1/2-x,+y,1/2-z	2.450(16)	138.4(9)
		O2A	H333	x,y,z	x,y,z	2.820(12)	126.3(6)
		O3A	H350	x,y,z	1/2-x,+y,1/2-z	2.720(14)	108.8(5)
		O3A	H351	x,y,z	1/2-x,+y,1/2-z	2.922(9)	140.6(5)
		O1B	H366	x,y,z	1/2+x,1-y,-1/2+z	3.08(5)	146.7(14)
		O1B	H301	x,y,z	3/2-x,+y,1/2-z	2.56(5)	133.1(14)
		O1B	H302	x,y,z	3/2-x,+y,1/2-z	2.88(6)	117.7(11)
		O2B	H310	x,y,z	3/2-x,+y,1/2-z	3.18(4)	107.3(9)
		O2B	H311	x,y,z	3/2-x,+y,1/2-z	2.59(4)	132.0(12)
		O3B	H302	x,y,z	x,y,z	2.69(6)	95(2)
		O3B	H310	x,y,z	x,y,z	2.74(4)	134.0(19)
	C-H ⋯ N	H14A	N308	x,y,z	x,y,z	2.852(6)	135.4(10)
	H ⋯ I	H3B	I303	x,y,z	3/2-x,y,1/2-z	3.069(4)	137.93(6)
Guest - Guest	H ⋯ Ar	H14B	C7A - C12A Cen.	x,y,z	1/2-x,+y,1/2-z	3.00(5)	147.2(11)
	O ⋯ H	O2A	H13B	x,y,z	1/2-x,+y,1/2-z	2.530(19)	119.2(5)
	O ⋯ Ar	O1A	C1A - C6A Cen.	x,y,z	1/2-x,+y,1/2-z	3.376(8)	118.7(9)

Table S29 BBA-8,12-OMe ring interactions

Type 1	Type 2	Component 1	Component 2	Symmetry Operator 1	Symmetry Operator 2	Cg – Cg / Å	α / °	Slippage / Å
Guest - Guest	Ar ⋯ Ar	C1A - C6A Cen.	C1A - C6A Cen.	x,y,z	1/2-x,+y,1/2-z	5.756(5)	6.9(4)	4.360
		C7A - C12A Cen.	C7A - C12A Cen.	x,y,z	1/2-x,+y,1/2-z	5.205(6)	15.3(4)	3.457

Table S30 BBA-8,10,12-OMe atom interactions

Type 1	Type 2	Component 1	Component 2	Symmetry Operator 1	Symmetry Operator 2	Length / Å	Angle / °
Host - Guest	H ··· Ar	H15A	C309 - C314 Cen.	x,y,z	3/2-x,1/2+y,3/2-z	2.934(9)	128(4)
	O ··· H	O1A	H311	x,y,z	-1/2+x,1/2+y,+z	2.60(4)	123.8(17)
		O1A	H332	x,y,z	x,y,z	2.54(4)	154.7(17)
		O2A	H322	x,y,z	x,y,z	2.83(3)	137.5(13)
		O2A	H333	x,y,z	x,y,z	2.58(2)	141.1(14)
		O3A	H332	x,y,z	1-x,+y,3/2-z	2.57(4)	132.6(17)
		O3A	H311	x,y,z	3/2-x,1/2+y,3/2-z	2.99(5)	104.3(17)
		O4A	H302	x,y,z	3/2-x,1/2+y,3/2-z	3.17(3)	106.5(14)
		O1B	H328	x,y,z	3/2-x,1/2-y,1-z	2.70(5)	154.0(13)
	C-H ··· N	H14A	N308	x,y,z	3/2-x,1/2+y,3/2-z	2.972(13)	137(2)
		H3B	N304	x,y,z	x,y,z	2.628(14)	168.6(2)
		H15F	N303	x,y,z	x,y,z	3.133(16)	144(5)
		H16E	N301	x,y,z	x,y,z	3.137(14)	167(3)
	H ··· I	H3A	I303	x,y,z	3/2-x,1/2+y,3/2-z	2.868(7)	139.67(10)
		H6A	I303	x,y,z	1/2-x.1/2+y,+z	3.279(7)	149.34(8)
		H16B	I301	x,y,z	3/2-x,1/2+y,3/2-z	3.194(4)	175(2)
		H1B	I306	x,y,z	3/2-x,1/2-y,1-z	3.133(6)	154.94(10)
		H6B	I302	x,y,z	-x,1-y,-1/2+z	3.086(5)	129.49(6)
Guest - Guest	H ··· Ar	H14C	C1B - C6B Cen.	x,y,z	-1/2+x,1/2+y,+z	3.155(6)	120(2)
	O ··· H	O1A	H15E	x,y,z	-1/2+x,1/2+y,+z	2.78(4)	151(6)
		O1B	H14B	x,y,z	2-x,y,1.5-z	3.01(6)	140(2)
		O3B	H16C	x,y,z	-1/2+x,1/2+y,+z	3.04(5)	160(3)

Table S31 BBA-8,10,12-OMe ring interactions

Type 1	Type 2	Component 1	Component 2	Symmetry Operator 1	Symmetry Operator 2	Cg – Cg / Å	α / °	Slippage / Å
Host - Guest	Ar ··· Ar	C301 - C305 Cen.	C7B - C12B Cen.	x,y,z	1/2-x,1/2+y,1/2-z	5.0199(4)	6.0	3.335
		C306 - C308 Cen.	C7B - C12B Cen.	x,y,z	1/2-x,1/2+y,1/2-z	3.8476(3)	8.0	0.999
		C314 - C318 Cen.	C7B - C12B Cen.	x,y,z	1/2-x,1/2+y,1/2-z	5.8665(5)	2.0	4.424

Table S32 PBA atom interactions

Type 1	Type 2	Component 1	Component 2	Symmetry Operator 1	Symmetry Operator 2	Length / Å	Angle / °
Host - Guest	H ··· Ar	H13F	C306 - C308 Cen.	x,y,z	1/2-x,1/2+y,1/2-z	2.71(3)	152.0(16)
		H13J	C306 - C308 Cen.	x,y,z	x,y,z	2.65(4)	144(2)
		C7E - C12E Cen.	H328	x,y,z	1-x,+y,1/2-z	3.00(3)	124.9(5)
	O ··· H	O1A	H304	x,y,z	x,y,z	3.170(17)	84.6(6)
		O1A	H305	x,y,z	x,y,z	3.584(14)	60.9(6)
		O1B	H335	x,y,z	1-x,+y,1/2-z	3.05(3)	101.8(16)
		O1C	H322	x,y,z	x,y,z	3.33(3)	71.7(12)
		O1C	H323	x,y,z	x,y,z	2.57(3)	101.8(15)
		O1D	H335	x,y,z	-1/2+x,-1/2+y,+z	2.92(4)	135(3)
		O1E	H328	x,y,z	1-x,y,1/-z	2.77(3)	124.2(16)
		O1E	H329	x,y,z	1-x,y,1/-z	2.92(3)	90.9(14)
		O1F	H304	x,y,z	1/2-x,-1/2+y,1/2-z	3.07(3)	113.0(10)
		O1F	H305	x,y,z	1/2-x,-1/2+y,1/2-z	2.96(2)	80.1(11)
	C-H ··· N	H3B	N304	x,y,z	1/2-x,1/2+y,1/2-z	2.81(2)	133.47(8)
		H12B	N311	x,y,z	1-x,+y,1/2-z	2.88(3)	163.89(9)
		H13F	N303	x,y,z	1/2-x,1/2+y,1/2-z	2.75(3)	164.6(15)
		H1C	N309	x,y,z	1-x,1-y,1-z	2.92(2)	106.6(19)
		H9C	N301	x,y,z	1-x,1-y,1-z	2.88(4)	149.43(8)
		H13J	N302	x,y,z	x,y,z	2.83(4)	139.8(18)
		H13J	N303	x,y,z	x,y,z	2.80(4)	160(3)
	H ··· I	H9E	I301	x,y,z	1/2+x,-1/2+y,z	3.28(3)	134.5(18)
		H12E	I301	x,y,z	1/2-x,-1/2+y,1/2-z	3.25(3)	135.6(19)

Guest - Guest	H ··· Ar	H11A	C1B - C6B Cen.	x,y,z	1/2-x,-1/2+y,1/2-z	3.05(4)	141.5(2)
		H10F	C1B - C6B Cen.	x,y,z	1/2-x,-1/2+y,1/2-z	3.43(4)	178.97(16)
	O ··· H	H13B	O1B	x,y,z	x,2-y,1/2+z	3.58(4)	88.7(14)
		H13C	O1B	x,y,z	x,2-y,1/2+z	3.25(4)	109.6(14)
		H2C	O1B	x,y,z	+x,2-y,1/2+z	2.94(2)	170(3)
		H3C	O1B	x,y,z	+x,2-y,1/2+z	2.69(4)	121(2)
		H2C	O1D	x,y,z	1/2-x,3/2-y,1-z	3.20(4)	119(2)
		H3C	O1D	x,y,z	1/2-x,3/2-y,1-z	2.94(2)	125(3)

Table S33 PBA ring interactions

Type 1	Type 2	Component 1	Component 2	Symmetry Operator 1	Symmetry Operator 2	Cg – Cg / Å	α / °	Slippage / Å
Host - Guest	Ar ··· Ar	C306 - C308 Cen.	C7B - C12B Cen.	x,y,z	1/2-x,-1/2+y,1/2-z	4.584(9)	9.5(7)	2.927
		C306 - C308 Cen.	C1E - C6E Cen.	x,y,z	x,y,z	4.508(12)	13.0	2.824
		C306 - C308 Cen.	C7F - C12F Cen.	x,y,z	1/2-x,1/2+y,1/-z	5.232(12)	20.0	4.493
		C314 - C318 Cen.	C1D - C6D Cen.	x,y,z	1/2+x,1/2+y,z	3.718(9)	4.1(7)	1.501
		C301 - C305 Cen.	C7A - C12A Cen.	x,y,z	x,y,z	5.988(6)	13.7(5)	4.934
		C319 - C323 Cen.	C7F - C12F Cen.	x,y,z	1/2-x,1/2+y,1/-z	4.522(12)	8.0	3.117
		C324 - C326 Cen.	C7B - C12B Cen.	x,y,z	1/2-x,-1/2+y,1/2-z	3.867(9)	8.4(7)	1.462
Guest - Guest	Ar ··· Ar	C7A - C12A Cen.	C7A - C12A Cen.	x,y,z	1/2-x,3/2-y,1-z	3.644(8)	0.0(7)	0.747
		C7F - C12F Cen.	C7F - C12F Cen.	x,y,z	1/2-x,1/2-y,-z	5.166(17)	0	3.561

Table S34 PBA-3-F atom interactions

Type 1	Type 2	Component 1	Component 2	Symmetry Operator 1	Symmetry Operator 2	Length / Å	Angle / °
Host - Guest	O ··· H	O1A	H301	x,y,z	1-x,+y1/2-z	2.52(2)	141.8(12)
		O1A	H302	x,y,z	1-x,+y1/2-z	2.98(2)	129.0(12)
		O1B	H328	x,y,z	x,y,z	2.82(2)	134.8(10)
		O1B	H329	x,y,z	x,y,z	2.62(2)	91.5(9)
	O ··· Ar	O1B	C332 - C336 Cen.	x,y,z	1/2-x,1/2+y,1/2-z	3.492(19)	119.4(11)
		O1C	C301 - C305 Cen.	x,y,z	1-x,+y,1/2-z	3.23(3)	137.9(16)
	C-H ··· N	H12A	N308	x,y,z	1/2-x,1/2+y,1/2-z	2.74(2)	134.36(8)
		H12B	N301	x,y,z	-1/2+x,1/2+y,+z	2.91(4)	166.93(10)
		H12C	N308	x,y,z	1/2-x,1/2+y,1/2-z	2.69(2)	134.36(8)
	H ··· I	H1BA	I304	x,y,z	1/2-x,3/2-y,1-z	3.02(4)	111.0(15)
		H2B	I303	x,y,z	+x,1+y,+z	3.29(3)	126.65(6)
Guest - Guest	H ··· Ar	H13F	C1A - C6A Cen.	x,y,z	+x,2-y,-1/2+z	3.17(6)	116(3)
	O ··· H	H2A	O1B	x,y,z	+x,2-y,-1/2+z	2.63(3)	133.8(6)
	X ··· H	F1B	H6B	x,y,z	1/2-x,5/2-y,1-z	2.34(4)	166.8(9)
		F1B	H10C	x,y,z	+x,2-y,-1/2+z	2.43(4)	174.2(7)

Table S35 PBA-3-F ring interactions

Type 1	Type 2	Component 1	Component 2	Symmetry Operator 1	Symmetry Operator 2	Cg – Cg / Å	α / °	Slippage / Å
Host - Guest	Ar ··· Ar	C301 - C305 Cen.	C7A - C12A Cen.	x,y,z	1-x,+y,1/2-z	3.828(7)	9.8(6)	1.826
		C309 - C313 Cen.	C1B - C6B Cen.	x,y,z	1-x,2-y,1-z	5.283(9)	16.0(8)	4.467
		C324 - C326 Cen.	C1A - C6A Cen.	x,y,z	1/2-x,-1/2+y,1/2-z	4.297(7)	18.5(6)	2.554
		C324 - C326 Cen.	C1C - C6C Cen.	x,y,z	1/2-x,-1/2+y,1/2-z	4.525(10)	13.4(9)	3.009
		C332 - C336 Cen.	C1C - C6C Cen.	x,y,z	1/2-x,-1/2+y,1/2-z	3.773(10)	18.3(9)	1.766

Table S36 PBA-2-F atom interactions

Type 1	Type 2	Component 1	Component 2	Symmetry Operator 1	Symmetry Operator 2	Length / Å	Angle / °
Host - Guest	H ··· Ar	C1A - C6A Cen.	H314	x,y,z	x,y,z	2.95(3)	122.5(4)
		H1DA	C309 - C311 Cen.	x,y,z	1-x,-y,1-z	3.06(2)	158.1(19)
	O ··· H	O1A	H304	x,y,z	x,y,z	2.490(17)	178.55(11)
		O1A	H315	x,y,z	x,y,z	2.741(17)	74.4(9)
		O1D	H313	x,y,z	x,y,z	2.66(3)	116.8(14)
	X ··· Ar	F1B	C314 - C318 Cen.	x,y,z	x,y,z	3.045(14)	119.8(8)
		F1D	C327 - C331 Cen.	x,y,z	1-x,y,3/2-z	3.62(3)	162.1(18)
	X ··· H	F1A	H330	x,y,z	3/2-x,1/2+x,3/2-z	2.90(2)	57.1(10)
		F1A	H331	x,y,z	3/2-x,1/2+x,3/2-z	2.470(17)	150.6(16)
		F1B	H317	x,y,z	1-x,1-y,1-z	2.810(14)	150.6(10)
		F1B	H318	x,y,z	1-x,1-y,1-z	2.560(16)	136.8(9)
	H ··· I	H1A	I304	x,y,z	1-x,-y,1-z	2.920(16)	138.7(9)
		H1BA	I305	x,y,z	x,y,z	3.371(12)	149.77(2)
		H10C	I302	x,y,z	3/2-x,1/2-y,1-z	3.31(3)	117.69(4)
		H13K	I304	x,y,z	1/2+x,1/2+z,y	3.01(5)	142(2)
Guest - Guest	H ··· Ar	H6C	C7A - C12A Cen.	x,y,z	3/2-x,3/2-y,1-z	2.66(3)	165.36(7)
	X ··· H	F1C	H1BA	x,y,z	x,y,z	2.95(3)	122(2)
		F1C	H6B	x,y,z	x,y,z	2.43(4)	131(2)
		F1C	H1D	x,y,z	1-x,1-y,1-z	2.69(5)	158(2)

Table S37 PBA-2-F ring interactions

Type 1	Type 2	Component 1	Component 2	Symmetry Operator 1	Symmetry Operator 2	Cg – Cg / Å	α / °	Slippage / Å
Host - Guest	Ar ··· Ar	C309 - C313 Cen.	C1D - C6D Cen.	x,y,z	1-x,-y,1-z	5.217(10)	18	3.466
		C319 - C323 Cen.	C7D - C12D Cen.	x,y,z	x,y,z	5.258(13)	14	4.265
Guest - Guest	Ar ··· Ar	C7D - C12D Cen.	C7D - C12D Cen.	x,y,z	1-x,y,3/2-z	4.603(15)	9	0.369

Table S38 PBA-2,6-I atom interactions

Type 1	Type 2	Component 1	Component 2	Symmetry Operator 1	Symmetry Operator 2	Length / Å	Angle / °
Host - Guest	O ··· H	O1A	H318	x,y,z	-x,1-y,1-z	2.72(2)	124.4(9)
		O1A	H323	x,y,z	-x,1-y,1-z	2.740(14)	146.9(10)
	X ··· Ar	I1B	C314 - C318 Cen.	x,y,z	-x,1-y,1-z	3.485(7)	132.4(8)
	X ··· H	I1A	H348	x,y,z	+x,1+y,+z	3.080(17)	106.9(5)
		I1A	H372	x,y,z	-x,2-y,1-z	3.206(12)	123.6(4)
		I2A	H336	x,y,z	-1+x,+y,+z	3.278(8)	139.3(4)
		I2A	H318	x,y,z	-x,1-y,1-z	3.159(9)	93.4(4)
		I1B	H346	x,y,z	x,y,z	3.010(18)	117.6(8)
		I1B	H347	x,y,z	x,y,z	3.180(18)	116.7(8)
		I2B	H328	x,y,z	-x,1-y,1-z	3.310(19)	86.8(7)
		I2B	H329	x,y,z	-x,1-y,1-z	3.250(17)	113.5(6)
Guest - Guest	O ··· H	O1A	H11B	x,y,z	-x,1-y,1-z	2.82(3)	66.5(7)
		O1B	H11A	x,y,z	-x,1-y,1-z	2.69(4)	147(2)

Table S39 PBA-2,6-I ring interactions

Type 1	Type 2	Component 1	Component 2	Symmetry Operator 1	Symmetry Operator 2	Cg – Cg / Å	α / °	Slippage / Å
Host - Guest	Ar ··· Ar	C342 - C344 Cen.	C7A - C12A Cen.	x,y,z	x,-1+y,z	4.826(11)	11.3(8)	3.236
		C350 - C354 Cen.	C7A - C12A Cen.	x,y,z	x,-1+y,z	3.780(10)	1.8(8)	1.718

Table S40 PBA-2-Me atom interactions

Type 1	Type 2	Component 1	Component 2	Symmetry Operator 1	Symmetry Operator 2	Length / Å	Angle / °
Host - Guest	H ··· Ar	H15B	C314 - C318 Cen.	x,y,z	1/2-x,1/2-y,1-z	3.04(5)	135.8(11)
	H ··· I	H14G	I301	x,y,z	1/2-x,-1/2+y,1/2-z	3.34(5)	107(3)
		H14H	I301	x,y,z	1/2-x,-1/2+y,1/2-z	3.25(5)	113(3)
		H9C	I305	x,y,z	x,y,z	3.22(4)	133.35(6)
Guest - Guest	H ··· Ar	H16C	C7A - C12A Cen.	x,y,z	1/2-x,1/2-y,1-z	2.68(4)	141(3)
	O ··· H	O1B	H10A	x,y,z	1/2-x,-1/2+y,1/2-z	2.95(3)	157(3)
		O1B	H13A	x,y,z	+x,-y,-1/2+z	2.91(3)	129(2)
		O1B	H15A	x,y,z	+x,-y,-1/2+z	2.90(4)	155(3)

Table S41 PBA-2-Me ring interactions

Type 1	Type 2	Component 1	Component 2	Symmetry Operator 1	Symmetry Operator 2	Cg – Cg / Å	α / °	Slippage / Å
Host - Guest	Ar ··· Ar	C309 - C313 Cen.	C7A - C12A Cen.	x,y,z	x,y,z	5.042(7)	4.1(6)	3.984
		C306 - C308 Cen.	C7A - C12A Cen.	x,y,z	x,y,z	5.780(6)	9.0(6)	4.863
		C301 - C306 Cen.	C1B - C6B Cen.	x,y,z	1/2-x,-1/2+y,1/2-z	4.494(6)	17.1(4)	2.100(14)
Guest - Guest	Ar ··· Ar	C1A - C6A Cen.	C1A - C6A Cen.	x,y,z	1/2-x,-1/2-y,1-z	5.156(6)	0.0(5)	3.952
		C7A - C12A Cen.	C7A - C12A Cen.	x,y,z	1/2-x,1/2-y,1-z	4.703(9)	0.0(8)	2.822

Table S42 PBA-2-Ph atom interactions

Type 1	Type 2	Component 1	Component 2	Symmetry Operator 1	Symmetry Operator 2	Length / Å	Angle / °
Host - Guest	H ··· Ar	C7A - C12A Cen.	H328	x,y,z	x,y,z	3.027(7)	131.0(6)
		C7A - C12A Cen.	H329	x,y,z	x,y,z	3.622(7)	110.2(6)
		C14A - C19A Cen.	H332	x,y,z	1-x,1-y,1-z	3.570(4)	118.4(4)
		C14A - C19A Cen.	H333	x,y,z	1-x,1-y,1-z	3.264(6)	129.9(4)
		C7A - C12A Cen.	H328	x,y,z	1-x,+y,3/2-z	2.935(7)	124.5(5)
		C7A - C12A Cen.	H329	x,y,z	1-x,+y,3/2-z	3.293(7)	113.1(6)
		H13F	C306 - C308 Cen.	x,y,z	x,y,z	2.747(5)	132(3)
		C14B - C19B Cen.	H304	x,y,z	x,y,z	3.331(4)	110.8(4)
		C14B - C19B Cen.	H305	x,y,z	x,y,z	2.914(6)	127.2(5)
	O ··· H	O1A	H329	x,y,z	1-x,+y,3/2-z	2.75(3)	119.0(16)
		O1A	H301	x,y,z	3/2-x,-1/2+y,3/2-z	3.19(3)	87.5(14)
		O1B	H336	x,y,z	1-x,2-y,1-z	2.730(12)	152(3)
	O-H ··· N	H1AA	N301	x,y,z	3/2-x,-1/2+y,3/2-z	3.361(7)	170(2)
	C-H ··· N	H13F	N302	x,y,z	x,y,z	2.820(5)	141(2)
	H ··· I	H1B	I306	x,y,z	3/2-x,3/2-y,1-z	3.134(8)	161.78(12)
		H15B	I306	x,y,z	3/2-x,3/2-y,1-z	3.278(8)	152.67(10)
Guest - Guest	O ··· H	H17A	O1B	x,y,z	-1/2+x,-1/2+y,+z	2.38(4)	128.3(9)
		H19B	O1B	x,y,z	3/2-x,3/2-y,1-z	3.37(5)	138.83(4)

Table S43 PBA-2-Ph ring interactions

Type 1	Type 2	Component 1	Component 2	Symmetry Operator 1	Symmetry Operator 2	Cg – Cg / Å	α / °	Slippage / Å
Host - Guest	Ar ⋯ Ar	C306 - C308 Cen.	C7B - C12B Cen.	x,y,z	x,y,z	4.453(11)	6.5(9)	2.821
		C314 - C318 Cen.	C7B - C12B Cen.	x,y,z	x,y,z	3.612(10)	2.9(9)	1.315
Guest - Guest	Ar ⋯ Ar	C1B - C6B Cen.	C1B - C6B Cen.	x,y,z	3/2-x,3/2-y,1-z	4.356(18)	0	1.146

S7. Group interaction summaries and additional analysis

Table S44 Group 1 summary of interactions

% Total Interactions				Average per Guest		
BBA-9-Me	BBA	BBA-3-Me		BBA-9-Me	BBA	BBA-3-Me
14.2	20.8	31.3	Ar ··· Ar	1.33	1.67	2.50
39.3	33.3	18.8	H ··· Ar	3.67	2.67	1.50
21.4	16.7	12.5	O ··· H	2.00	1.33	1.00
10.7	4.2	6.3	O ··· Ar	1.00	0.33	0.50
-	-	-	X ··· Ar	-	-	-
-	-	-	X ··· H	-	-	-
3.6	8.3	6.3	OH ··· N	0.33	0.67	0.50
7.1	16.7	6.3	CH ··· N	0.67	1.33	0.50
3.6	-	6.3	H ··· I	0.33	-	1.50

Table S45 Group 2 summary of interactions

% Total Interactions			Average per Guest	
BBA	BBA-10-CN		BBA	BBA-10-CN
20.8	23.5	Ar ··· Ar	1.67	2.00
33.3	11.8	H ··· Ar	2.67	1.00
16.7	5.9	O ··· H	1.33	0.50
4.2	-	O ··· Ar	0.33	-
-	5.9	X ··· Ar	-	0.50
-	-	X ··· H	-	-
8.3	5.9	OH ··· N	0.67	0.50
16.7	41.2	CH ··· N	1.33	3.50
-	5.9	H ··· I	-	0.50

Table S46 Group 3 summary of interactions

% Total Interactions				Average per Guest		
PBA-12-OMe	BBA-8,12-OMe	BBA-8,10,12-OMe		PBA-12-OMe	BBA-8,12-OMe	BBA-8,10,12-OMe
12.0	10.5	12.0	Ar ⋯ Ar	1.50	1.00	1.50
24.0	5.3	8.0	H ⋯ Ar	3.00	0.50	1.00
48.0	68.4	44.0	O ⋯ H	6.00	6.50	5.50
-	5.3	-	O ⋯ Ar	-	0.50	-
-	-	-	X ⋯ Ar	-	-	-
-	-	-	X ⋯ H	-	-	-
4.0	-	-	OH ⋯ N	0.50	-	-
8.0	5.3	16.0	CH ⋯ N	1.00	0.50	2.00
4.0	5.3	20.0	H ⋯ I	0.50	0.50	2.50

Table S47 Group 4 summary of interactions

% Total Interactions					Average per Guest			
PBA	PBA-3-F	PBA-2-F	PBA-2,6-I		PBA	PBA-3-F	PBA-2-F	PBA-2,6-I
23.1	25.0	13.6	12.5	Ar ··· Ar	1.50	1.67	0.75	1.00
12.8	5.0	13.6	-	H ··· Ar	0.83	0.33	0.75	-
41.0	25.0	13.6	25.0	O ··· H	2.67	1.67	0.75	2.00
-	10.0	-	-	O ··· Ar	-	0.67	-	-
-	-	9.1	6.3	X ··· Ar	-	-	0.50	0.50
-	10.0	31.8	50.0	X ··· H	-	0.67	1.75	4.00
-	-	-	-	OH ··· N	-	-	-	-
17.9	15.0	-	-	CH ··· N	1.17	1.00	-	-
5.1	10.0	18.2	-	H ··· I	0.33	0.67	1.00	-

S7.1. Tabulation of Group 3 O···H interactions

Table S48 Full tabulation of Group 3 O ··· H interactions

Guest	Type 1	Type 2	Atom 1	Atom 2	Length / Å	Angle / °
PBA-12-OMe	O ··· H (Host-Guest)	O ··· H (Hydroxyl)	O1A	H331	2.75(3)	128.0(12)
			O1B	H305	2.76(2)	139.0(13)
			O1B	H312	3.358(19)	103.1(9)
			O1B	H313	2.55(2)	125.4(11)
		O ··· H (Methoxy)	O2A	H335	3.056(15)	124.5(8)
			O2A	H336	2.540(18)	142.9(9)
			O2B	H330	2.57(2)	117.4(13)
	O ··· H (Guest-Guest)	O ··· H (Hydroxyl)	O1A	H3B	2.84(3)	146.05(9)
			O1A	H14E	2.548(18)	103.48(13)
		O ··· H (Methoxy)	O2A	H2B	3.362(16)	108.9(11)
			O2B	H1AA	2.47(4)	114.9(19)
			O2B	H13C	3.51(3)	100.9(8)
BBA-8,12-OMe	O ··· H (Host-Guest)	O ··· H (Hydroxyl)	O1A	H340	2.740(16)	95.6(9)
			O1A	H341	2.450(16)	138.4(9)
			O1B	H366	3.08(5)	146.7(14)
			O1B	H301	2.56(5)	133.1(14)
			O1B	H302	2.88(6)	117.7(11)
		O ··· H (Methoxy)	O2A	H333	2.820(12)	126.3(6)
			O3A	H350	2.720(14)	108.8(5)
			O3A	H351	2.922(9)	140.6(5)
			O2B	H310	3.18(4)	107.3(9)
			O2B	H311	2.59(4)	132.0(12)
			O3B	H302	2.69(6)	95(2)
			O3B	H310	2.74(4)	134.0(19)
	O ··· H (Guest-Guest)	O ··· H (Methoxy)	O2A	H13B	2.530(19)	119.2(5)

BBA-8,10,12-OMe	O ··· H (Host-Guest)	O ··· H (Hydroxyl)	O1A	H311	2.60(4)	123.8(17)
			O1A	H332	2.54(4)	154.7(17)
			O1B	H328	2.70(5)	154.0(13)
		O ··· H (Methoxy)	O2A	H322	2.83(3)	137.5(13)
			O2A	H333	2.58(2)	141.1(14)
			O3A	H332	2.57(4)	132.6(17)
			O3A	H311	2.99(5)	104.3(17)
			O4A	H302	3.17(3)	106.5(14)
	O ··· H (Guest-Guest)	O ··· H (Hydroxyl)	O1A	H15E	2.78(4)	151(6)
			O1B	H14B	3.01(6)	140(2)
		O ··· H (Methoxy)	O3B	H16C	3.04(5)	160(3)

S7.2. Measurements for oxygen deviation of Group 1

Table S49 Group 1 oxygen deviation away from C1 – C6 aromatic ring plane

	Exchange Site	Angle between MPLN(C1 – C6 Cen.) and MPLN(C5 – C14 – O1) /°
BBA-9-Me	A1	13.6(16)
	A2	86.4(15)
	B	22(2)
	C	30(3)
BBA	A	12(2)
	B	83(4)
	C	6(4)
BBA-3-Me	A	2.9(14)
	B1	28(4)
	B2	8(3)

S7.3. Intermolecular interactions as an aid for atom assignment

Distinction between atom elements is often a crucial component of analytical chemistry and associated with high-quality crystallographic analysis which can readily make this distinction. This demand has continued with CS analysis as highlighted by a 2015 paper by Fujita and co-workers which determined the oxidation positions for a family of cyclic sesquiterpenes. (Zigon *et al.*, 2015) While it is often possible to identify changes to molecular structure and connectivity with the CS method, it remains difficult to distinguish between identical substitution positions with similar electron counts. This idea is showcased by PBA-3-F in Group 4; the structure possesses symmetrical *ortho*-substitution of the C1 – C6 ring with hydroxyl and fluorine functionalities, which have an identical number of electrons. In traditional small molecule crystallography, this assignment would primarily be tested by monitoring crystallographic metrics such as R_1, wR_2, and goodness-of-fit (GooF). However, due to the low occupancy exchange of the guest molecules and presence of heavy atoms in the framework, the different variations in atom assignment make little difference to these metrics, as shown below in Table S50.

Table S50 Variation of traditional crystallographic metrics when modelling PBA-3-F

Modelling Variant	R_1 / %	wR_2 / %	GooF
A3 – B3 – C3	5.76	19.52	1.033
A5 – B3 – C3	5.76	19.51	1.033
A3 – B5 – C3	5.76	19.54	1.031
A3 – B3 – C5	5.76	19.50	1.036
A5 – B5 – C3	5.76	19.47	1.039
A5 – B3 – C5	5.76	19.49	1.034
A3 – B5 – C5	5.77	19.53	1.031
A5 – B5 – C5	5.77	19.54	1.030

Where A/ B/C represent the respective exchange sites within the asymmetric unit, and 3/5 represent the ring position modelled with fluorine.

The lack of difference in traditional statistics therefore necessitates a different approach for accurate atom assignment. For PBA-3-F, the knowledge of fluorine's poor hydrogen bonding ability (Dunitz & Taylor, 1997) was taken into account alongside knowledge of the interactions adopted within the pore. This was expected to result in the hydroxyl group adopting the most interactions of the two functionalities. Therefore, the atoms could be distinguished through assignments which maximised interactions for the hydroxyl groups. This also led to the fluorine groups adopting the interactions

with the shortest distances which further supported this method for distinction of element types. The utilisation of interaction analysis, in combination with knowledge of the elemental interaction tendencies, is recommended as a tool which can be implemented in all future CS analysis for aiding atom assignment.

S8. References

Biradha, K., Hongo, Y. & Fujita, M. (2002). *Angew. Chemie - Int. Ed.* **41**, 3395–3398.

Dolomanov, O. V., Bourhis, L. J., Gildea, R. J., Howard, J. A. K. & Puschmann, H. (2009). *J. Appl. Cryst.* **42**, 339–341.

Dunitz, J. D. & Taylor, R. (1997). *Chem. - A Eur. J.* **3**, 89–98.

Groom, C. R., Bruno, I. J., Lightfoot, M. P. & Ward, S. C. (2016). *Acta Crystallogr. Sect. B Struct. Sci. Cryst. Eng. Mater.* **72**, 171–179.

Ramadhar, T. R., Zheng, S. L., Chen, Y. S. & Clardy, J. (2015). *Chem. Commun.* **51**, 11252–11255.

Sheldrick, G. M. (2015). *Acta Crystallogr.* **C71**, 3–8.

Spek, A. L. (2003). *J. Appl. Crystallogr.* **36**, 7–13.

Zigon, N., Hoshino, M., Yoshioka, S., Inokuma, Y. & Fujita, M. (2015). *Angew. Chemie Int. Ed.* **54**, 9033–9037.
